# Long-time analytic approximation of large stochastic oscillators: Simulation, analysis and inference

**DOI:** 10.1371/journal.pcbi.1005676

**Published:** 2017-07-24

**Authors:** Giorgos Minas, David A. Rand

**Affiliations:** 1 Zeeman Institute for Systems Biology & Infectious Disease Epidemiology Research, University of Warwick, Coventry, United Kingdom; 2 Mathematics Institute, University of Warwick, Coventry, United Kingdom; University of Pittsburgh, UNITED STATES

## Abstract

In order to analyse large complex stochastic dynamical models such as those studied in systems biology there is currently a great need for both analytical tools and also algorithms for accurate and fast simulation and estimation. We present a new stochastic approximation of biological oscillators that addresses these needs. Our method, called phase-corrected LNA (pcLNA) overcomes the main limitations of the standard Linear Noise Approximation (LNA) to remain uniformly accurate for long times, still maintaining the speed and analytically tractability of the LNA. As part of this, we develop analytical expressions for key probability distributions and associated quantities, such as the Fisher Information Matrix and Kullback-Leibler divergence and we introduce a new approach to system-global sensitivity analysis. We also present algorithms for statistical inference and for long-term simulation of oscillating systems that are shown to be as accurate but much faster than leaping algorithms and algorithms for integration of diffusion equations. Stochastic versions of published models of the circadian clock and NF-*κ*B system are used to illustrate our results.

## Author summary

Many cellular and molecular systems such as the circadian clock and the cell cycle are oscillators that are modelled using nonlinear dynamical systems. Moreover, oscillatory systems are ubiquitous elsewhere in science. There is an extensive theory for perfectly noise-free dynamical systems and very effective algorithms for simulating their temporal behaviour. On the other hand, biological systems are inherently stochastic and the presence of stochastic noise can play a crucial role. Unfortunately, there are far fewer analytical tools and much less understanding for stochastic models especially when they are nonlinear and have lots of state variables and parameters. Moreover simulation is not so effective and can be very slow if the system is large. In this article we describe how to accurately approximate such systems in a way that facilitates fast simulation, parameter estimation and new approaches to analysis, such as calculating probability distributions that describe the system’s stochastic behaviour and describing how these distributions change when the parameters of the system are varied.

## Introduction

Dynamic cellular oscillating systems such as the cell cycle, circadian clock and other signaling and regulatory systems have complex structures, highly nonlinear dynamics and are subject to both intrinsic and extrinsic stochasticity. Moreover, current models of these systems have high-dimensional phase spaces and many parameters. Modelling and analysing them is therefore a challenge, particularly if one wants to both take account of stochasticity and develop an analytical approach enabling quantification of various aspects of the system in a more controlled way than is possible by simulation alone. The stochastic kinetics that arise due to random births, deaths and interactions of individual species give rise to Markov jump processes that, in principle, can be analyzed by means of master equations. However, these are rarely tractable and although an exact numerical simulation algorithm is available [1], for the large systems we are interested in, this is very slow.

It is therefore important to develop accurate approximation methods that enable a more analytical approach as well as offering faster simulation and better algorithms for data fitting and parameter estimation. A number of approximation methods aimed at accelerating simulation are currently available. This includes leaping algorithms [2, 3] and algorithms for integration of diffusion equations (or chemical Langevin equations (CLE)) [4] that provide faster simulation. However, these methods do not provide analytical tools for studying the dynamics of the system and they can also be extremely slow for data fitting and parameter estimation. One obvious candidate for overcoming these limitations is the Linear Noise Approximation (LNA). The LNA is based on a systematic approximation of the master equation by means of van Kampen’s Ω-expansion [5] which uses the system size parameter Ω that controls the number of molecules present in the system. The large system size validity of the LNA has been shown in [6], in the sense that the distribution of the Markov jump process at a fixed finite time converges, as the system size Ω tends to ∞, to the LNA probability distribution. The latter distribution is analytically tractable allowing for fast estimation and simulation algorithms. However, the LNA has significant limitations, particularly in approximating long-term behaviour of oscillatory systems.

We show below that for the oscillatory systems that we study, the LNA approximation of the distribution *P*_*t*_ = *P*(*Y*(*t*)|*Y*(0)), of the state *Y*(*t*) of the system at some time *t* becomes inaccurate when the time *t* is greater than a few periods of the oscillation. However, if we rather consider a similar system which in the Ω → ∞ limit instead of a limit cycle has an equilibrium point that is linearly stable, then the LNA approximation of *P*_*t*_ remains accurate for a much longer time-scale. For example, in Fig C in S3 Appendix we give an example where the LNA fails in a matter of a period or two for the oscillatory system, but for the corresponding equilibrium system it is very accurate for over a hundred times as long (and probably much longer). Similar behaviour is also observed in other systems and using different measures in [8].

The observation that non-degenerate limit cycles have such linearised stability in the directions transversal to the limit cycle suggests the way forward for oscillatory systems. Our approach exploits the fact that, because of this transversal linearised stability, the distributions *P*_*t*_ for a general class of systems with a stable attracting limit cycle in the Ω → ∞ limit are, like the above fixed point systems, similarly well-behaved on long time-scales provided one conditions *P*_*t*_ on appropriate transversal sections to the limit cycle.

We introduce a modified LNA, called the phase-corrected LNA, or pcLNA, that exploits the above observations to overcome the most important shortcomings of the LNA and we develop methods for analysis, simulation and inference of oscillatory systems that are accurate for much larger times. We build on previous work of Boland et al. [9] which uses the 2-dimensional Brusselator system as an exemplar to investigate the failure of the LNA in approximating long-term behaviour of oscillatory systems and presents a method for computing power spectra and comparing exact simulations with LNA predictions of the same phase rather than time. Using various low-dimensional oscillatory systems for illustration, a related analysis has been employed to study the temporal variability of oscillatory systems in the tangental direction of the Ω → ∞ limit cycle [10] and/or the amplitude variability in the transversal direction of the limit cycle [11–13]. Other papers derive related descriptions of the asymptotic phase of stochastic oscillators [14, 15].

We extend these results in a number of ways including the following: (i) we develop a theory that treats the general case and provide analytical arguments that justify our approximations and enable computation of trajectory distributions, (ii) we show that the approach is practicable for larger nonlinear systems, (iii) we present a new powerful system-global sensitivity theory for such systems using measures such as the Fisher Information Matrix and the Kullback-Leibler divergence that are analytically computed, (iv) we present a simulation algorithm and show it is as accurate but faster than leaping and integration of diffusion equation algorithms, and (v) we derive the Kalman filter associated with the pcLNA in order to provide a practical way to accurately approximate the likelihood function thus facilitating estimation of system parameters *θ* and predictive algorithms. The approach in [9] uses transversal sections which are normal to the limit cycle. We follow this but in the supplementary information (S1 Sects. 8.2 & 8.3) we show that for most considerations one can use any transversal to the limit cycle, including those defined in [14, 15].

To illustrate and validate our approach we apply it to a relatively large published stochastic model of the *Drosophila* circadian clock due to Gonze et al. [16] (see S2 Sect. 1). This model involves 10 state variables and 30 reactions and its structure is discussed in S2. The large system limit is given by the differential equation system of 10 kinetic equations that are listed in the supplementary information (S2 Sect. 1) along with the reaction scheme of the system. The stochastic version of the Brusselator system and a stochastic version of a well-studied model of the NF-*κ*B signalling system [17] are also used to illustrate our methods and the results can be found in S3 Appendix.

These systems are free-running oscillators in the sense that they correspond to a limit cycle of an autonomous differential equation in the the Ω → ∞ limit. However, our results also apply to the equally important classes of entrained forced oscillators and damped oscillations. We therefore consider two such systems in S2 and S3: the light-entrained *Drosophila* circadian clock model of [18] which is an example of a forced oscillator and the NF-*κ*B system model [17]. The latter has the extra feature that the analysis is not concerned with a limit cycle but of a transient solution that converges to the limit cycle as time increases. This solution is the biologically interesting one that describes how the system responds to being stimulated by TNF*α*. The supplementary information S1 includes technical derivations and S2 and S3 contain further illustrative figures that we refer to in this paper.

## Results

Stochastic models of cellular processes in signaling and regulatory systems are usually described in terms of reaction networks. A system of multiple different molecular subpopulations has state vector, *Y*(*t*) = (*Y*_1_(*t*), …, *Y*_*n*_(*t*))^*T*^ where *Y*_*i*_(*t*), *i* = 1, …, *n*, denotes the number of molecules of each species at time *t*. These molecules undergo a number of possible reactions (e.g. transcription, translation, degradation) where the reaction of index *j* changes *Y*(*t*) to *Y*(*t*) + *ν*_*j*_, *ν*_*j*_ ∈ ℝ^*n*^. The vectors *ν*_*j*_ are called stoichiometric vectors. Each reaction occurs randomly at a rate *w*_*j*_(*Y*(*t*)) (often called the intensity of the reaction), which is a function of *Y*(*t*) but also depends periodically on *t* when we are studying forced oscillators. Such systems can be exactly simulated using the Stochastic Simulation algorithm (SSA) [1].

It is common in studying stochastic systems to introduce a system size Ω which is a parameter that occurs in the intensities of the reactions *w*_*j*_(*Y*(*t*)) and controls molecular numbers (see discussion in S1 Sect. 2). While having a system size parameter is not necessary to apply our methods, it allows one to study the dependence of stochastic fluctuations upon system size and to calculate the deterministic equations that describe the evolution of the concentration vector *X*(*t*) = *Y*(*t*)/Ω in the limit of Ω → ∞ (see S1 Sect. 3). Although more general conditions can be used, a condition that will be sufficient for our purposes is that the rates *w*_*j*_(*Y*(*t*)) depend upon Ω as *w*_*j*_(*Y*) = Ω*u*_*j*_(*Y*/Ω) (cf. [5–7]).

In the limit Ω → ∞ the time dependence of *X*(*t*) is given by the solution *x*(*t*) of the differential equation

$$\begin{array}{c} \dot{x}=F\left(x\right),\;F\left(x\right)=\sum_{j}\nu_{j}u_{j}\left(x\left(t\right)\right), \end{array}$$ (1)

with the appropriate initial condition (see S1 Sect. 3). For free-running oscillators the differential equation Eq (1) is autonomous, whereas for forced oscillators *F* also depends periodically on *t*.

Throughout we will be interested in the case where the solution *x*(*t*) of interest is a stable limit cycle *γ* of minimal period *τ* > 0 given by *x* = *g*(*t*), 0 ≤ *t* ≤ *τ*. We shall also always assume the generic situation for stable limit cycles of autonomous systems in which one of the characteristic exponents of the limit cycle is equal to zero and the rest have negative real part ([19], [20 Sect 1.5] and S1 Sect. 1). For an entrained forced oscillator all the characteristic exponents of the limit cycle are assumed to have negative real parts.

**Fig 1:**
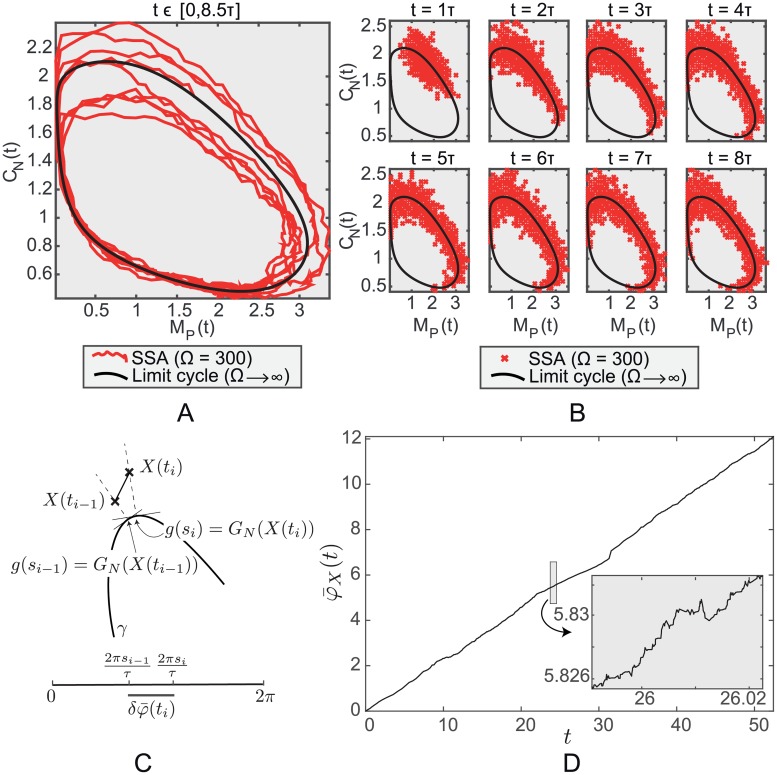
Exact stochastic simulation of the *Drosophila* circadian clock system. (A) A stochastic trajectory obtained by running the SSA over the time-interval *t* ∈ [0, 8.5*τ*] and (B) SSA samples (*R* = 3000) at times *t* = *τ*, 2*τ*, …, 8*τ*. The concentrations, *X*(*t*) = *Y*(*t*)/Ω, of two (out of 10) of the species are displayed (*per* mRNA *M*_*P*_ (x-axis) and nuclear PER-TIM complex *C*_*N*_ (y-axis)). For this model the system size Ω is Avogadro’s number in units of nM^−1^ multiplied by the cell volume in litres and the concentrations are nanomolar. The value of Ω used here is 300 in units of nM which assumes a cell volume of approximately 0.5 × 10^−12^ litres. The number of *M*_*P*_ molecules ranges from 0 to 1200 and the number of *C*_*N*_ molecules from 100 to 900. It is only through Ω that the cell volume appears in the equations. The black solid curve is the Ω → ∞, limit cycle solution. (C) Schematic diagram illustrating the mapping *G*_*N*_(⋅) and the relative phase *δφ*_*X*_(⋅). (D) A plot of the lifted phase function ${\bar{\varphi }}_{X}\left(t\right)$ for a trajectory of this system. Note the long-term linear increase combined with frequent reversals.

Fig 1(A) displays a stochastic trajectory of the concentrations *X*(*t*) = *Y*(*t*)/Ω of two of the species of the circadian clock system obtained using exact SSA simulation over a period of time *t* ∈ [0, 8.5*τ*] where *τ* ≈ 26.98 hours is the period of the limit cycle *γ*. Here the system size is Ω = 300, imposing moderate to high levels of stochasticity (see also Table C in S2 Appendix). Results for system sizes Ω = 200, 500 and 1000 are also reported in Fig B in S2 Appendix. Fig 1(B) shows realizations of the key probability distributions

$$\begin{array}{c} P\left(X\left(t\right)|X\left(t_{0}\right)=x_{0}\right),\;t>t_{0}, \end{array}$$ (2)

which describe the state of the system at some time *t* > *t*_0_. It is very rare that accurate analytical approximations for such probability distributions can be derived from the exact Markov-jump process when *t* is large. Furthermore, as we can see, the SSA samples of *P*(*X*(*t*)|*X*(*t*_0_) = *x*_0_) spread along the curved periodic solution, *x* = *g*(*t*), of the limiting (Ω → ∞) deterministic system, implying that for large *t* this distribution is far from being normal and has a complex structure.

However, we will show that there are important related distributions that can be well approximated even for large times *t*. For example, for each point *x* on the Ω → ∞ limit cycle *γ*, consider the (*n* − 1)-dimensional hyperplane $S_{x}$ normal to *γ* at *x* ∈ *γ* (i.e. orthogonal to the tangent vector *F*(*x*) at *x*). We will show that the distribution of the intersection points of stochastic trajectories *X*(*t*) with $S_{x}$ can be well-approximated by a multivariate normal (MVN) distribution that can be relatively easily calculated.

We need to define more precisely what we mean by intersection points. Consider the mapping *G*_*N*_ defined by *G*_*N*_(*X*) = *x* ∈ *γ* if *X* ∈ $S_{x}$ (see Fig 1(C)). Suppose that the limit cycle is given by *x* = *g*(*t*) and extend *g*(*t*) to all *t* ∈ ℝ by periodicity. Now consider a stochastic trajectory *X*(*t*_*i*_), *i* = 0, 1, 2, …. Suppose that *G*_*N*_(*X*(*t*_*i* − 1_)) = *g*(*s*_*i* − 1_), *i* > 0. If *G*_*N*_(*X*(*t*_*i*_)) = *g*(*s*) then it equals *g*(*s* + *qτ*) for all integers *q*. Choose *q* so that *s*_*i*_ = *s* + *qτ* satisfies *s*_*i* − 1_ − *τ*/2 ≤ *s*_*i*_ < *s*_*i* − 1_ + *τ*/2. Note that the difference between *s*_*i*_ and *s*_*i* − 1_ can be both positive and negative and the choice of *s*_*i*_ minimises |*s*_*i*_ − *s*_*i* − 1_|. We define the relative phase *δφ*_*X*_(*t*_*i*_) to be 2*π*(*s*_*i*_ − *s*_*i* − 1_)/*τ*. With this definition the lifted phase ${\bar{\varphi }}_{X}$ is defined to be the function

$$\begin{array}{c} {\bar{\varphi }}_{X}\left(t_{0}\right)=0\;\text{and}\;{\bar{\varphi }}_{X}\left(t_{i}\right)=\sum_{k=1}^{i}\delta \varphi_{X}\left(t_{i}\right)=2\pi \left(s_{i}-s_{0}\right)/\tau \;\text{if}\;i\geq 1. \end{array}$$

An example of ${\bar{\varphi }}_{X}\left(t\right)$ is shown in Fig 1(C). Now, as shown in that figure, if the system size is not too small, although there will be some reversals, the stochastic process *G_N_*(*X*(*t*)), *t* > 0, will move around *γ* in the direction of the deterministic flow given by Eq (1) so that ${\bar{\varphi }}_{X}\left(t\right)$ increases at a definite positive rate because phase advances exceed retreats. Our approximations (S1 Sect. 6) give that the long term rate is 2*π*/*τ* and that the variance of the fluctuations ${\bar{\varphi }}_{X}\left(t\right)-2\pi t/\tau$ grows linearly with a growth rate that is O(*t*/Ω).

Now consider stochastic trajectories *X*(*t*_*i*_) with *X*(*t*_0_) ∈ $S_{g(T_{0})}$ and consider how this trajectory passes through $S_{g(T_{1})}$. We can assume that *T*_0_ < *T*_1_ ≤ *T*_0_ + *τ* by the periodicity of *g*. The *r*th pass will occur between the first time *t*_*i*_ when

$$\begin{array}{c} {\bar{\varphi }}_{X}\left(t_{i}\right)\leq 2\pi \left(r-1\right)+2\pi \left(T_{1}-T_{0}\right)/\tau <{\bar{\varphi }}_{X}\left(t_{i+1}\right) \end{array}$$

and *t*_*i*+1_. Therefore, we define the *first intersection point of the rth pass* to be

$$\begin{array}{c} Q_{x_{1}}^{\left(r\right)}=Q_{x_{1}}^{\left(r,X\right)}=X\left(t_{i}\right) \end{array}$$

since *X* does not change between *t*_*i*_ and *t*_*i*+1_.

**Fig 2:**
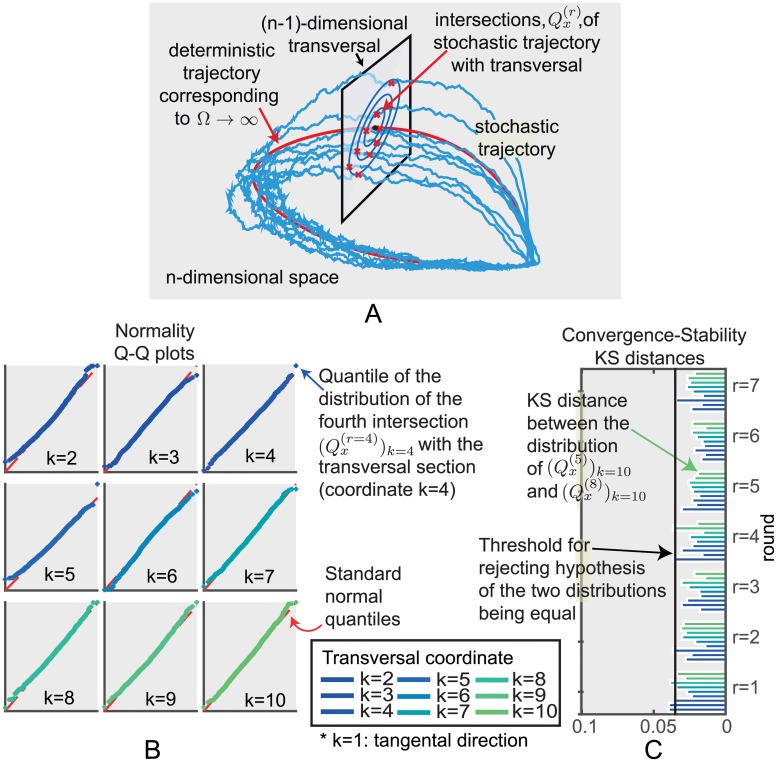
Intersections of the stochastic trajectories with a transversal section $S_{g(t_{0})}$. (A) Schematic representation of the intersections. (B) We consider an adapted coordinate system (*x*_1_, …, *x*_10_) (see S1 Sect. 1) so that (*x*_2_, …, *x*_10_) are orthogonal coordinates on $S_{g(t_{0})}$ and then consider the distribution *P*^*k*, *r*^ of the values of *x*_*k*_ for *k* = 2, …, 10 at the intersection points $Q_{g(t_{0})}^{\left(r\right)}$. Quantile-Quantile (Q-Q) plots of these distributions for the fourth pass, $Q_{g(t_{0})}^{\left(r=4\right)}$, show that the distributions are very close to being normal, (see Fig C in S2 Appendix for similar plots for *r* = 1, 2, …, 8). (C) KS distances between the above distributions for the first *r* = 1, 2, …, 7 rounds of the stochastic trajectory and the distribution in the 8th round (*r* = 8), *k* = 2, 3, …, 10.

These points of intersection $Q_{x}^{\left(r\right)}$ describe the stochasticity of the system around a particular phase *x* of the system. The above ideas work equally well with any transversal to *γ*. For example, the time at which the *i*-th variable is maximal in the deterministic system is given by the transversal submanifold Σ defined by ${\dot{x}}_{i}=0,{\ddot{x}}_{i}<0$ so the intersection of the stochastic trajectory with Σ can be regarded as giving the statistics of the maxima of the stochastic trajectory (see Fig 2(A)). Close to the limit cycle, Σ is well-approximated by its tangent space at the point of intersection with the limit cycle. Therefore, these transversal distributions are useful for analysing various aspects of the system.

Our first observation is that the empirical transversal distributions

$$\begin{array}{c} P_{x_{1}}^{\left(r\right)}=P\left(Q_{x_{1}}^{\left(r,X\right)}|X\left(t_{0}\right)=x_{0}\right) \end{array}$$

obtained by exact simulation are approximately multivariate normal (Fig 2(B)). Moreover, as *r* increases $P_{x_{1}}^{\left(r\right)}$ and $P_{x_{1}}^{\left(r+1\right)}$ are hardly distinguishable and appear to converge to a fixed, approximately normal transversal distribution as *r* increases (Fig 2(C)). Similar results hold if we use a different family of transversal sections to *γ* as explained in S1 Sect. 8.2 & 8.3.

A natural question that arises is whether one obtains a different distribution when instead of taking the first intersection point of the *r*th pass one takes a later intersection point of the same round. This is addressed in S1 Sect. 12 where we show that in exact simulations there appears to be no difference as would be expected from the LNA approximation.

### The Linear Noise Approximation (LNA)

The convergence of the transversal distributions to approximately normal distributions naturally raises the question of whether asymptotic approximation methods such as the LNA, which provide multivariate normally distributed approximation of the stochastic system, can be used to accurately approximate these transversal distributions.

The LNA as formulated by [6] is derived directly from the underlying Markov jump process and is valid for any time interval of finite fixed length. It is based on the ansatz

$$\begin{array}{c} X\left(t\right)=\frac{Y(t)}{\Omega }=x\left(t\right)+\frac{\xi (t)}{\sqrt{\Omega }} \end{array}$$ (3)

where *x*(*t*) is a solution of the limiting (Ω → ∞) deterministic system Eq (1) and $\xi \left(t\right)/\sqrt{\Omega }$ describes the stochastic variations. In our case we always take *x*(*t*) to be the periodic solution *g*(*t*). A key aspect of this ansatz is that *ξ*(*t*) satisfies a linear stochastic differential equation that is independent of Ω, with drift and diffusion matrices that are functions of the deterministic solution *g*(*t*). Details are given in S1 Sect. 4 & 5.

Given an initial time *t*_0_ and an initial condition *ξ*(*t*_0_) for *ξ*, the LNA determines the distribution, of *ξ*(*t*), *t* > *t*_0_, and hence $X\left(t\right)=g\left(t\right)+\xi \left(t\right)/\sqrt{\Omega }$ that we respectively denote by *P*_LNA_(*ξ*(*t*)|*t*_0_, *ξ*(*t*_0_)) and *P*_LNA_(*X*(*t*)|*t*_0_, *ξ*(*t*_0_)). If *ξ*(*t*_0_) is only known up to a multivariate normal (MVN) distribution *P*_0_ then we denote these distributions, respectively, by *P*_LNA_(*ξ*(*t*)|*t*_0_, *ξ*(*t*_0_)∼*P*_0_) and *P*_LNA_(*X*(*t*)|*t*_0_, *ξ*(*t*_0_)∼*P*_0_). Details of how to calculate these distributions are given in S1 Sect. 4. Each of the above distributions is MVN enabling analytical approaches, for example in analysing the stochastic sensitivities of the system.

If we fix *t* > *t*_0_ then as Ω → ∞ the true distribution of *ξ* converges to the distribution *P*_LNA_(*ξ*(*t*)|*t*_0_, *ξ*(*t*_0_)) (see e.g. [5]). However, one most certainly cannot reverse the limits i.e. for a fixed Ω one cannot expect the approximation to hold for large time *t* → ∞. As we now show, this is certainly the case for oscillators and we aim to overcome this limitation by developing methods that remain accurate for much larger times than the LNA.

**Fig 3:**
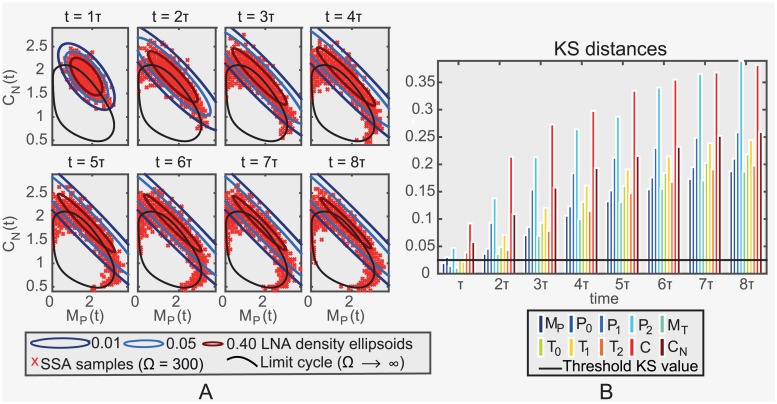
Comparison between LNA and exact simulations. (a) Samples (in nanomolar concentrations) obtained from the SSA simulation algorithm (red crosses) and 0.01, 0.05, 0.40 contours of the LNA probability density (colored ellipsoids) at fixed times, *t* = *τ*, 2*τ*, …, 8*τ* (*τ*: minimal period), for the circadian clock system (*M*_*P*_ *per* mRNA; *C*_*N*_ nuclear PER:TIM complex *P*_0_ & *T*_0_ PER & TIM protein; *P*_1_ & *T*_1_ phosphorylated PER & TIM protein; *P*_2_ & *T*_2_ twice phosphorylated PER & TIM protein; *C* cytoplasmic PER:TIM complex, units as in Fig 1). The limit cycle ODE solution is also displayed (black solid line). (b) KS distance between the empirical distribution of SSA samples and the LNA distribution of each species (different colors, see legend) at the fixed times. The threshold level is also displayed (black solid line). The system size is Ω = 300.

We first consider the distribution *P*(*X*(*t*)|*X*(0) = *x*_0_) and compare this for SSA simulated samples and the LNA at a sequence of times *t* = *τ*, 2*τ*, …, 8*τ* and for an arbitrary (fixed) initial state *x*_0_ ∈ *γ*. As we can see in Fig 3, the LNA fits the SSA simulations relatively well in the short run (*t* ≤ *τ*), but as time progresses the Kolmogorov-Smirnov (KS) distance between the two distributions for each state variable for the LNA and the SSA increases substantially beyond the threshold level (see Fig 3(B)). The LNA predictions spread along the tangental direction and therefore fail to accurately reflect the SSA samples that have instead spread along the curved limit cycle.

On the other hand, as we saw earlier, the transversal distributions $P_{x_{1}}^{\left(r\right)}=P\left(Q_{x_{1}}^{\left(r\right)}|X\left(t_{0}\right)=x_{0}\right)$ of the *Drosophila* circadian clock system are approximately normal (Fig 3(B)). We next derive an approximation of $P_{x_{1}}^{\left(r\right)}$ under the LNA and show that it accurately approximates $P_{x_{1}}^{\left(r\right)}$ for the *Drosophila* circadian clock, Brusselator and NF-*κ*B systems.

### Calculating transversal distributions

We now generalise slightly and consider the set of stochastic trajectories *X* where the initial conditions *X*(*t*_0_) have a MVN distribution *P*_0_ that is supported on the normal transversal section $S_{g(t_{0})}$ (denoted *X*(*t*_0_)∼*P*_0_). We consider how to approximate the distribution $P_{x_{1}}^{\left(r,P_{0}\right)}$ of the intersection points $Q_{g(t)}^{\left(r,X\right)}$ of these trajectories with the normal transversal section $S_{g(t_{1})}$, *t*_1_ > *t*_0_. As an approximation we take the conditional distribution

$$\begin{array}{c} P_{\text{LNA},t_{1}}^{\left(r\right)}=P_{\text{LNA}}(X\left(t_{1}^{\left(r\right)}\right)|X\left(t_{1}^{\left(r\right)}\right)\in S_{g(t_{1})},\xi \left(t_{0}\right)∼P_{0}),\;t_{1}^{\left(r\right)}=t_{1}+\left(r-1\right)\tau \end{array}$$ (4)

given by conditioning $P_{\text{LNA},t_{1}}^{\left(r,\text{free}\right)}=P_{\text{LNA}}(X\left(t_{1}^{\left(r\right)}\right)|\xi \left(t_{0}\right)∼P_{0})$ on $X\left(t_{1}^{\left(r\right)}\right)\in \;S_{g\left(t_{1}\right)}$. It gives a MVN distribution supported on $S_{g(t_{1})}$.

**Fig 4:**
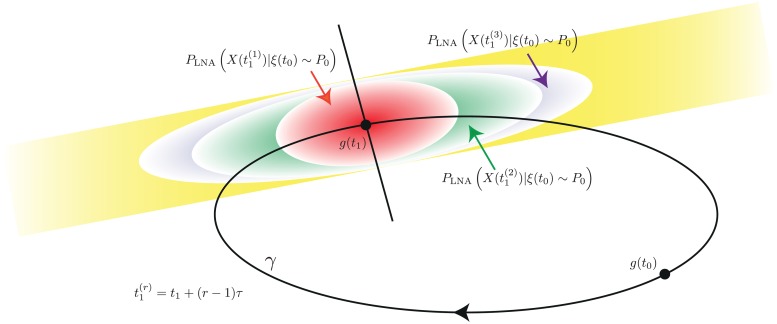
Schematic diagram of the distributions $P_{\text{LNA}}\left(X\left(t_{1}^{\left(r\right)}\right)|\xi \left(t_{0}\right)∼P_{0}\right)$ as r increases. The yellow distribution represents the limit as *r* → ∞. Although, for free oscillators the latter distribution diverges, the corresponding conditional distributions on the transversal converge.

In S1 we show that, although, for free-running oscillators, $P_{\text{LNA},t_{1}}^{\left(r,\text{free}\right)}$ diverges as *r* → ∞, the mean and covariance of the MVN transversal distribution $P_{\text{LNA},x_{1}}^{\left(r\right)}$ converge exponentially fast to those of a MVN distribution $P_{\text{LNA},x_{1}}^{\left(\infty \right)}$ (S1 Sect. 8, cf. Fig 4). The distribution $P_{\text{LNA},x_{1}}^{\left(\infty \right)}$ is a fixed point in the sense that if the distribution of *X*(*t*_1_ + *τ*) is given by the LNA using as initial condition $\xi \left(t_{1}\right)∼P_{\text{LNA},x_{1}}^{\left(\infty \right)}$ then conditioning on *X*(*t*_1_ + *τ*) ∈ $S_{g(t_{1})}$ gives

$$\begin{array}{c} \left(X\left(t_{1}+\tau \right)|X\left(t_{1}+\tau \right)\in S_{g(t_{1})}\right)∼P_{\text{LNA},x_{1}}^{\left(\infty \right)}. \end{array}$$

Using this fact enables us to calculate $P_{\text{LNA},x_{1}}^{\left(\infty \right)}$ directly because we show in S1 that its mean and covariance matrix satisfy a simple fixed point equation that is easily solved numerically (S1 Sect. 9.1).

The reader will note that in Eq (4) we approximate by conditioning on $X\left(t_{1}^{\left(r\right)}\right)\in S_{g(t_{1})}$ whereas we should have conditioned on *X*(*t*) ∈ $S_{g(t_{1})}$ for arbitrary *t* corresponding to the *r*th round. In S1 Sect. 7 we argue that the error in the mean and variance of the distribution due to taking $t=t_{1}^{\left(r\right)}$ is O(Ω^−1^).

**Fig 5:**
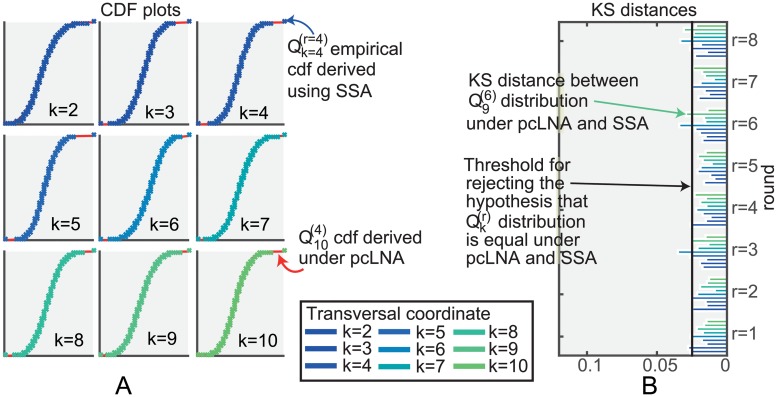
Comparison of pcLNA and exact transversal distributions for the *Drosophila* circadian clock (Ω = 300). (A) CDF plots of the distributions *P*^*k*, *r*^ as in Fig 2 for the pcLNA (red line) and the SSA (empirical CDF, crosses) for *k* = 2, 3, …, 10 and round *r* = 4 (see Fig D in S2 Appendix for *r* = 1, 2, …, 8). (B) KS distances between the corresponding distributions under pcLNA and SSA, *r* = 1, 2, …, 8, *k* = 2, 3, …, 10.

The question remains as to how well these distributions capture the exact simulation transversal distribution $P_{x_{1}}^{\left(r\right)}$. This is addressed in Fig 5 where it is shown that the fit is excellent even for Ω as low as 300. The fit is even better for higher system sizes (Fig E in S2 Appendix). In S3 we also show similar low Ω results for the Brusselator (Fig B in S3 Appendix) and the NF-*κ*B system (Fig E in S3 Appendix). The result is also true for the light-entrained *Drosophila* circadian clock system (see Fig J in S2 Appendix) and the transient oscillations of the NF-*κ*B system (see Fig H in S3 Appendix). Thus we note that although the LNA cannot be used directly to accurately compute *P*(*X*(*t*)|*X*(0)) for a fixed Ω and increasing *t*, using it to compute the transversal distributions provides accurate estimates of $P_{x_{1}}^{\left(r\right)}$ for much larger times *t*_1_ + *rτ*. Moreover, in S1 Sects. 8.2 & 8.3 we also explain why the convergence of the distribution on normal hyperplanes implies convergence on other transversal sections to *γ*.

In S1 Sect. 8.4. we explain that in contradistinction to free-running oscillators, for entrained forced oscillators, *P*_*r*_ = *P*_LNA_(*X*((*r* − 1)*τ* + *t*_1_)|*x*_0_, *ξ*_0_ ∼ *P*_0_) converges as *r* → ∞ so that, under the LNA, the phase fluctuation have a variance that is bounded independently of *r*. The corresponding conditional distribution is therefore a correspondingly good approximation to the transversal distribution $P_{x_{1}}^{\left(r,P_{0}\right)}$ (see Fig J in S2 Appendix). However, it does not mean that $P_{\text{LNA},t_{1}}^{\left(r,\text{free}\right)}$ is a good approximation to the corresponding distribution $P(X\left(t_{1}^{\left(r\right)}\right)|X\left(0\right))$ for an exact simulation. In fact, we show in Fig I in S2 Appendix that $P_{\text{LNA},t_{1}}^{\left(r,\text{free}\right)}$ is a poor approximation of the empirical distribution $P(X\left(t_{1}^{\left(r\right)}\right)|X\left(0\right))$ derived from exact simulations for the light-entrained *Drosophila* circadian clock (Ω = 300). The bounded variance of the phase fluctuations as *r* → ∞ for forced oscillators is the basic mechanism behind the population-level entrainment of stochastic oscillators introduced in [21].

### Stochastic fluctuations in periods and timing

We now consider the fluctuations *δt* in the time taken for the lifted phase of a stochastic trajectory to go from a given phase *φ*_1_ to a greater one *φ*_2_. If *φ*_2_ − *φ*_1_ = 2*rπ* then this corresponds to the time taken to perform *r* cycles.

**Fig 6:**
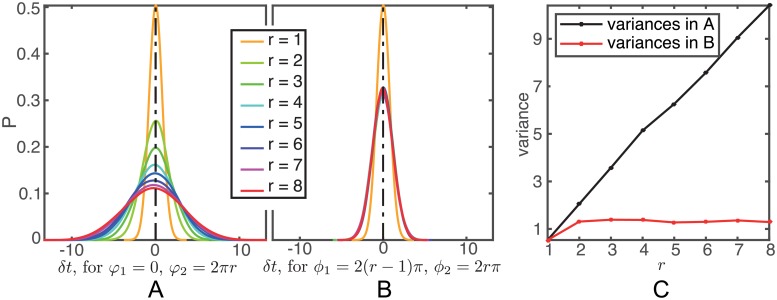
Exact empirical distribution of the fluctuations *δt* in *Drosophila* circadian clock system size Ω = 300. (A) The (smoothed) empirical density function of *δt* in the time taken for the lifted phase of a stochastic trajectory to go from *φ*_1_ = 0 to *φ*_2_ = 2*rπ*, *r* = 1, 2, …, 8. (B) The (smoothed) empirical density function of the fluctuations in the time taken for the lifted phase of a stochastic trajectory to go from *φ*_1_ = 2(*r* − 1)*π* to *φ*_2_ = 2*rπ*, *r* = 1, 2, …, 8. (C) The variance of the distributions in (A) and (B).

In Fig 6 we give an example using the *Drosophila* circadian clock model where we take *φ*_1_ to be 0 (with a fixed initial condition *x*_0_ ∈ *γ*) and *φ*_2_ = 2*πr* for *r* = 1, …, 8. The distributions of *δt* appear to be very close to normal and the variance appears to grow linearly with *r*. We also consider the case where *φ*_1_ = 2(*r* − 1)*π* and *φ*_2_ = 2*rπ* for *r* = 1, …, 8. Again the distributions are approximately normal but the variances are approximately constant (Fig 6(C)). Because for a given *r* the trajectory has done *r* − 1 cycles before reaching the lifted phase *φ*_1_ the distribution of the state at this phase is changing with *r*. We expect that this distribution is converging with increasing *r* and this result (Fig 6(C)) is in accordance with this.

In S1 Sect. 6 we approximate the statistics of *δt* using the LNA and show that as a random variable *δt* is approximately normal with mean that is O(Ω^−3/2^) and we also calculate its variance up to terms that are O(Ω^−3/2^) and the extent of its divergence from normality.

All points with a given lifted phase *φ*_1_ lie in a particular transversal $S_{g(t_{1})}$ with 0 ≤ *t*_1_ < *τ*. If *t*_2_ = *t*_1_ + (*φ*_2_ − *φ*_1_)*τ*/2*π*, then, the mean and variance of *δt* can be calculated in terms of *t*_1_ and *t*_2_. If the initial conditions *ξ*(*t*_1_) are MVN distributed on $S_{g(t_{1})}$ with mean 0 and covariance *V*_1_, this variance is $\left(1/\alpha^{2}\Omega \right)\;{\overset{ˇ}{V}}_{11}+\text{O}\left(\Omega^{-3/2}\right)$ where ${\overset{ˇ}{V}}_{11}=\overset{ˇ}{V}{\left(t_{1},t_{2}\right)}_{11}$,

$$\begin{array}{c} \overset{ˇ}{V}\left(t_{1},t_{2}\right)=C\left(t_{1},t_{2}\right)V_{1}C{\left(t_{1},t_{2}\right)}^{T}+V\left(t_{1},t_{2}\right). \end{array}$$

written in adapted coordinates at *g*(*t*_2_) (see S1 Sect. 1). All terms on the right hand side of this equation are defined in S1 Sect. 4. The above exact simulations of the *Drosophila* circadian clock agree with these theoretical predictions. It is easy to see (cf. S1 Sect. 8) that ${\overset{ˇ}{V}}_{11}$ grows roughly linearly with *t*_2_ − *t*_1_.

### pcLNA simulation algorithm

Given the ability to accurately approximate the transversal distributions and the results in [9] we realised it should be possible to use this to construct a rapid simulation algorithm. The linear increase of the variance of the deviations *δt*, or equivalently, the linear growth in the variance of the deviation of the lifted phase ${\bar{\varphi }}_{X}\left(t_{i}\right)$ from 2*πt*_*i*_/*τ*, indicates the reasons for the long-time failure of the standard LNA. It is unable to cope with the increasing phase deviations. This motivates the phase correction approach used in the simulation algorithm we now define.

The approach is to amend the LNA Ansatz *X*(*t*) = *g*(*t*) + Ω^−1/2^ *ξ*(*t*) to *X*(*t*) = *g*(*s*) + Ω^−1/2^ *κ*(*s*) where *g*(*s*) = *G_N_*(*X*(*t*)) and to use resetting of *t* to *s* to cope with the growth in the variance of ${\bar{\varphi }}_{X}\left(t_{i}\right)-2\pi t_{i}/\tau$ keeping the LNA fluctuation *κ*(*s*) normal to *γ*. While for free-running oscillators the variance of *ξ*(*t*) grows without bound as *t* increases, *κ*(*s*) has uniformly bounded variance.

The pcLNA simulation algorithm iteratively uses standard LNA steps of length Δ*τ* to move from a state *X*(*s*_*i* − 1_) to a new state *X*(*s*_*i* − 1_ + Δ*τ*) = *X*_*i*_, *i* = 1, 2, …. After each LNA step, the phase of the system is reset or “corrected” such that *g*(*s*_*i*_) = *G*_*N*_(*X*_*i*_) and the (global) fluctuations *ξ*(*s*_*i* − 1_ + Δ*τ*) = Ω^1/2^(*X*_*i*_ − *g*(*s*_*i* − 1_ + Δ*τ*)) are replaced by the normally transversal fluctuation *κ*(*s*_*i*_) = Ω^1/2^(*X*_*i*_ − *g*(*s*_*i*_)) which are MVN distributed and, as we showed in the previous section, approximate well the transversal fluctuations under the exact Markov Jump process.

**Fig 7:**
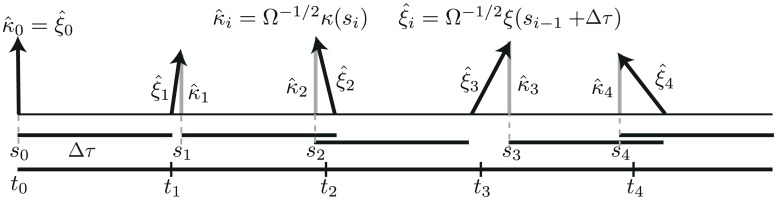
The main step in the pcLNA algorithm. The solid horizontal bars below the horizontal axis are all of length Δ*τ*, the basic time step of the algorithm. The black arrows show ${\hat{\xi }}_{i}=\Omega^{-1/2}\xi \left(s_{i-1}+\Delta \tau \right)$ and the grey arrows ${\hat{\kappa }}_{i}=\Omega^{-1/2}\kappa \left(s_{i}\right)$. Having calculated $\hat{\kappa }\left(s_{i-1}\right)$ one uses *κ*(*s*_*i* − 1_) as the initial state and updates it using the LNA and a time-step Δ*τ* to obtain *ξ* at *s*_*i* − 1_ + Δ*τ*. Then *ξ*(*s*_*i* − 1_ + Δ*τ*) is replaced by *κ*(*s*_*i*_) so that *g*(*s*_*i* − 1_ + Δ*τ*) + Ω^−1/2^ *ξ*(*s*_*i* − 1_ + Δ*τ*) = *g*(*s*_*i*_) + Ω^−1/2^ *κ*(*s*_*i*_) where *κ*(*s*_*i*_) is normal to the limit cycle. Therefore, *s*_*i*_ gives the phase of *κ*(*s*_*i*_) and the corresponding time is *t*_*i*_ = *t*_0_ + *iΔτ*.

The steps of the pcLNA simulation algorithm are described next in more detail (see also Fig 7).

1. Choose a time-step size Δ*τ* > 0.
2. Input **initial conditions** *κ*(*s*_0_) and *X*_0_ = *g*(*s*_0_) + Ω^−1/2^ *κ*(*s*_0_).
3. **For iteration** *i* = 1, 2, …
  - sample *ξ*(*s*_*i* − 1_ + Δ*τ*) from MVN(*C*_*i*_ *κ*(*s*_*i* − 1_), *V*_*i*_);
  - compute *X*_*i*_ = *g*(*s*_*i* − 1_ + Δ*τ*) + Ω^−1/2^ *ξ*(*s*_*i* − 1_ + Δ*τ*);
  - set *s*_*i*_ to be such that *G_N_*(*X*_*i*_) = *g*(*s*_*i*_) and *κ*_*i*_ = Ω^1/2^(*X*_*i*_ − *g*(*s*_*i*_)).

In the for loop *C*_*i*_ = *C*(*s*_*i* − 1_, *s*_*i* − 1_ + Δ*τ*) and *V*_*i*_ = *V*(*s*_*i* − 1_, *s*_*i* − 1_ + Δ*τ*) are the drift and diffusion matrices in the linear SDE describing the evolution of the noise process *ξ*(*t*) under the LNA (see S1 Sect. 4).

The simulated sample *X*_*i*_ corresponds to time *t*_*i*_ = *t*_0_ + *iΔτ*, *i* = 1, 2, …, where *t*_0_ is the initial time. The time *t*_*i*_ is not necessarily equal to the phase *s*_*i*_, defined by the relation *g*(*s*_*i*_) = *G*_*N*_(*X*_*i*_), which is stochastic and has variance linearly increasing with the time step Δ*τ*.

If one wants to record simulated trajectories at a finer time-scale than Δ*τ* then one can run the algorithm with Δ*τ* replaced by Δ*τ*/*M* for some integer *M* > 1 and only carry out the phase correction in step 3(c) every *M*th step and at all the other steps just proceeding as in the standard LNA (ignoring step 3(c)). This gives the same distribution as if the intermediate points had not been calculated because of the transitive nature of the LNA i.e. the distribution *P*_LNA_(*X*(*s* + *t*)|*X*(0)) is equal to the distribution *P*_LNA_(*X*(*t*)|*X*(*s*)∼*P*_LNA_(*X*(*s*)|*X*(0))). In the simulation results described below the time-step Δ*τ* = 6 hours and *M* = 3 so that there are *τ*/6 ≈ 4.5 corrections in every round of the limit cycle. The effect of less frequent correction is studied in S2 Sect. 5.

#### Comparisons to other simulation algorithms

We compared the pcLNA simulation algorithm in terms of both CPU time and precision of the approximation with some of the most important alternatives, the tau-leap method and integration of the chemical Langevin equation (CLE) method. Exact simulations produced by the SSA are also used as a reference for the comparison.

For the tau-leap simulation we use the algorithm described in [3], which is a refinement of the original tau-leap method first proposed in [2]. In [3] the authors suggest an optimal method to compute the largest possible time step such that the leap condition is still approximately satisfied. The leap condition ensures that the state change in any time step is small enough so that no rate function will experience a macroscopically significant change in its value. The error of this approximation is controlled by a parameter *ϵ*. For integrating the CLE described in [4], we use the classical Euler method (see [32]) with a fixed time step Δ*t*. The integration of the CLE can be done using methods that include higher-order terms in the integration and this has been shown to improve the speed of implementation in low-dimensional systems, albeit with a cost in the complexity of the algorithm (see [33, 34]). However, we are not aware of any implementations of these methods for high-dimensional systems such as those considered here, or of comparisons of such methods to the Euler method for such problems.

For both the tau-leap and the CLE approximation we explored different values of their parameters *ϵ* and Δ*t* to attain a good balance of precision and CPU time. Here we present the results for the largest values of both *ϵ* and Δ*t*, hence smallest CPU time, which attain similar performance with the pcLNA algorithm in terms of precision. If little improvement could be achieved in terms of precision by lowering either *ϵ* or Δ*t*, the larger values are preferred.

The algorithms are implemented for a fixed time-interval (8.5 times the period of the limit cycle of the system) and the comparison is made at 8 fixed time points using the KS distances between the empirical distribution of each algorithm and the empirical distribution derived using the SSA simulations. Note that for all approximation algorithms considered here, the probability of generating negative populations is non-zero and there are a number of methods for dealing with this. Our simple approach is described in S1 Sect. 13.

**Fig 8:**
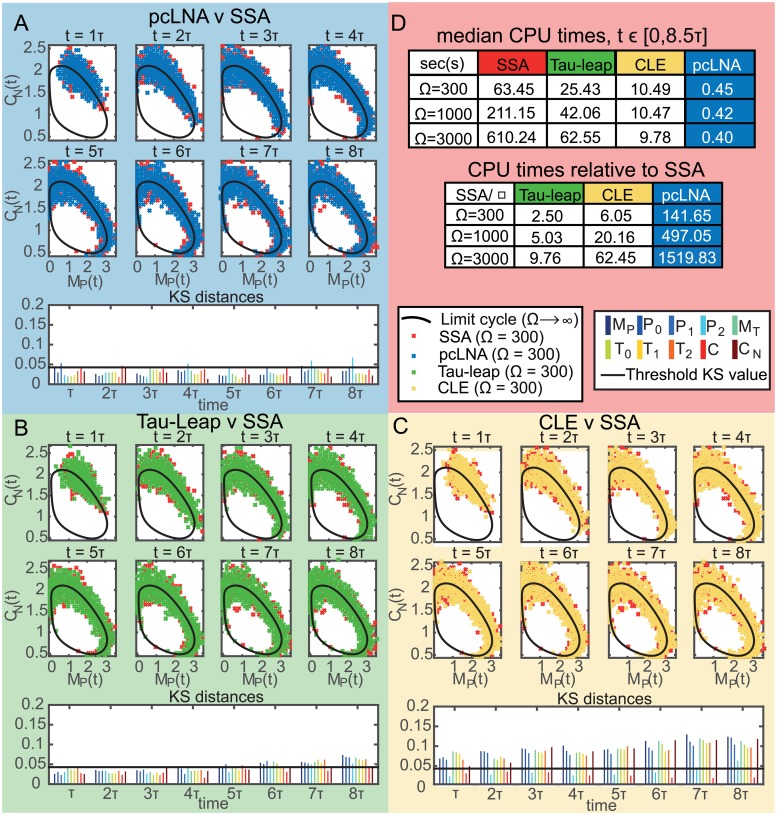
Comparison between pcLNA, tau-leap and CLE simulation algorithms for the *Drosophila* circadian clock. Panels (A), (B) and (C) contain the samples (in concentrations, units as in Fig 1) produced by respectively the pcLNA, tau-leap (*ϵ* = 0.002) and CLE (Δ*t* = 0.002) algorithms and the exact simulation (SSA) at time-points *t* = *τ*, 2*τ*, …, 8*τ*, for Ω = 300, along with the KS distances between the empirical distributions of each approximation and the SSA for each system variable (coloured bars; variable names as in Fig 3). Panel (D) provides the median CPU times for a single trajectory run in the time-interval [0, 8.5*τ*] under the different simulation algorithms along with the ratio of the median CPU time under SSA and each approximation algorithm.

Fig 8 displays the median CPU times for a single trajectory simulation in *t* ∈ [0, 8.5*τ*], under the competing approaches for different system sizes, Ω = 300, 1000, 3000, along with a comparison in terms of precision for Ω = 300 (see Figs G & H in S2 Appendix for Ω = 1000 & 3000). A sample of size *R* = 2000 is produced for each algorithm. We see that the precision of all approximation methods is fairly similar, with their empirical distributions almost indistinguishable to exact simulations. In terms of CPU times, we see that the SSA is much slower than the other algorithms particularly for large system sizes. The tau-leap offers some improvement to the CPU times but this is relatively small compared to the CLE approximation and especially the pcLNA algorithm. One reason for this relatively small improvement for the tau-leap algorithm is the stiffness of the considered system, a property that is however very common in biological systems and it is known to slow down the tau-leap method by requiring small values of the *ϵ* parameter to ensure that the leap condition is satisfied and hence the approximation is fairly precise. Note that for similar reasons, a small Δ*t* was necessary to achieve good precision with the CLE approximation.

As we can see in Fig 8, the pcLNA algorithm is about 24 times faster than the CLE approximation, tens to hundreds of times faster than the tau-leap and hundreds to thousands of times faster than the SSA. In our simulations, it took 0.4sec to derive this long-time trajectory, which means that in about 7 minutes one can generate more than 1000 trajectories of this large system over a long-time compared to about 2.7 hours with CLE approximation and much longer times for the other methods. Therefore, the pcLNA offers a substantial improvement in CPU times compared to standard approaches in simulating oscillatory systems without compromising the precision of the simulation substantially.

Perhaps more importantly, this pcLNA simulation algorithm has the advantage of being based on an analytical framework that allows calculation of some key distributions. Therefore, it enables more rigorous methods for assessing accuracy and robustness.

### Analysis and inference of oscillatory systems using pcLNA

The derivation of analytical expressions of the transversal distributions allows us to analyse various aspects of the stochastic behaviour of these systems that can possibly involve a large number of variables and parameters. Here we illustrate the use of pcLNA transversal distributions to perform such an analysis. We begin by describing the pcLNA joint distribution of multiple intersections to possibly different transversal sections on the limit cycle and then discuss Fisher information, sensitivity analysis and estimation by Kalman filtering.

#### Joint distribution on multiple transversals

**Fig 9:**
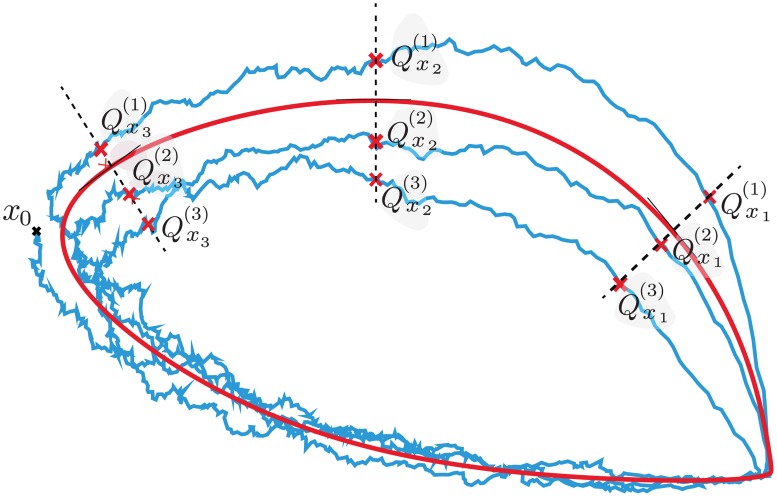
The sequence *Q* in two-dimensions. The stochastic trajectory *X*(*t*) (blue line), initiated from *x*_0_, intersects each of the transversal sections *x*_1_, *x*_2_ and *x*_3_ (dashed lines) of the limit cycle (red solid line) three times, following a path $Q_{x_{1}}^{\left(1\right)}\to Q_{x_{2}}^{\left(1\right)}\to Q_{x_{3}}^{\left(1\right)}\to Q_{x_{1}}^{\left(2\right)}\to Q_{x_{2}}^{\left(2\right)}\to Q_{x_{3}}^{\left(2\right)}\to Q_{x_{1}}^{\left(3\right)}\to Q_{x_{2}}^{\left(3\right)}\to Q_{x_{3}}^{\left(3\right)}$.

Consider *q* phase states of the limit cycle *x*_*i*_ = *g*(*t*_*i*_), *i* = 1, …, *q*, on *γ* where 0 ≤ *t*_1_ < *t*_2_ < … < *t*_*q*_ < *τ*. If *X*(*t*) is a stochastic trajectory, we consider how it meets the transversal sections at the *x*_*i*_ as *t* increases using the lifted phase function ${\bar{\varphi }}_{X}$. We can talk of the times when *G_N_*(*X*(*t*)) first takes the phase *x*_*i*_ during the *r*-th revolution of *G_N_*(*X*(*t*)) around *γ*. Using ${\bar{\varphi }}_{X}$ to define the points $Q_{g(t)}^{\left(r\right)}$ we have that it first meets $S_{x_{i}}$ in $Q_{x_{i}}^{\left(1\right)}$ for *i* = 1, …, *q*. If *i* < *q* then we let $Q_{x_{i+1}}^{\left(k\right)}$ denote the first point in $S_{x_{i+1}}$ that *X* meets after it leaves $Q_{x_{i}}^{\left(k\right)}$. If *i* = *q* then the next transversal it meets is $S_{x_{1}}$ and the intersection point is $Q_{x_{1}}^{\left(k+1\right)}$. In this way (see Fig 9) we derive a sequence of intersection points $\underline{Q}=Q_{x_{1}}^{\left(1\right)},\ldots ,Q_{x_{q}}^{\left(1\right)},Q_{x_{1}}^{\left(2\right)},\ldots ,Q_{x_{q}}^{\left(2\right)},\ldots ,Q_{x_{1}}^{\left(m\right)},\ldots ,Q_{x_{q}}^{\left(m\right)}$.

We shall be interested in the distribution

$$\begin{array}{c} P\left(\underline{Q}|X\left(t_{0}\right)\right)=P\left(Q_{x_{1}}^{\left(1\right)},\ldots ,Q_{x_{q}}^{\left(m\right)}|X\left(t_{0}\right)\right). \end{array}$$ (5)

Remarkably, in our approximation, this distribution is MVN with a covariance matrix whose inverse has a simple tridiagonal form in terms of the drift and diffusion matrices coming from the LNA (S1 Sects. 9.2 & 9.3).

The fact that the above transversal distributions are MVN allows us to analytically compute the Fisher Information matrix and associated quantities that can be used to perform a stochastic sensitivity analysis of oscillatory systems.

### Fisher information

Fisher Information quantifies the information that an observable random variable carries about an unknown parameter *θ*. If *P*(*X*, *θ*) is a probability distribution depending on parameters *θ*, the Fisher Information Matrix (FIM) *I* = *I*_*P*_ has entries

$$\begin{array}{c} I_{ij}=E[\frac{\partial \ell }{\partial \theta_{i}}\frac{\partial \ell }{\partial \theta_{j}}]=-E[\frac{\partial^{2}\ell \left(\theta ;X\right)}{\partial \theta_{i}\partial \theta_{j}}], \end{array}$$ (6)

where ℓ = log *P*, and *θ*_*i*_ and *θ*_*j*_ are the *i*th and *j*th components of the parameter *θ*. If *P* is MVN with mean and covariance *μ* = *μ*(*θ*) and Σ = Σ(*θ*) then

$$\begin{array}{c} I_{ij}={\frac{\partial \mu }{\partial \theta_{i}}}^{T}\Sigma^{-1}\frac{\partial \mu }{\partial \theta_{j}}+\frac{1}{2}\text{tr}(\Sigma^{-1}\frac{\partial \Sigma }{\partial \theta_{i}}\Sigma^{-1}\frac{\partial \Sigma }{\partial \theta_{j}}). \end{array}$$ (7)

The FIM measures the sensitivity of *P* to a change in parameters in the sense that

$$\begin{array}{c} D_{KL}\left(P\left(\cdot ,\theta +\delta \theta \right),P\left(\cdot ,\theta \right)\right)=\frac{1}{2}\delta \theta^{T}I_{P}\delta \theta +{\text{O}(\|\delta \theta \|}^{3}) \end{array}$$

where *D*_*KL*_ is the Kullback-Leibler divergence. The significance of the FIM for sensitivity and experimental design follows from its role in Eq (6) as an approximation to the Hessian of the log-likelihood function at a maximum. Assuming non-degeneracy, if θ* is a parameter value of maximum likelihood there is a *s* × *s* orthogonal matrix *V* such that, in the new parameters *θ*′ = *V* ⋅ (*θ* − θ*),

$$\begin{array}{c} \ell \left(\theta \right)\approx \ell \left(\theta^{*}\right)-\sum_{i}\sigma_{i}^{2}{\theta_{i}^{\prime }}^{2}. \end{array}$$

for *θ* near θ*. From these facts it follows that the $\sigma_{i}^{2}$ are the eigenvalues of the FIM and that the matrix *V* diagonalises it. If we assume that the *σ*_*i*_ are ordered so that $\sigma_{1}^{2}\geq \ldots \geq \sigma_{s}^{2}$ then it follows that near the maximum the likelihood is most sensitive when $\theta_{1}^{\prime }$ is varied and least sensitive when $\theta_{s}^{\prime }$ is. Moreover, *σ*_*i*_ is a measure of this sensitivity.

The theory of optimal experimental design is based on the idea of trying to make the *σ*_*i*_ decrease as slowly as possible so that the likelihood is as peaked as possible around the maximum, thus maximising the information content of the experimental sampling methods. Various criteria for experimental design have been proposed including D-optimality that maximises the determinant of the FIM and A-optimality that minimises the trace of the inverse of the FIM [6]. Diagonal elements of the inverse of FIM constitute a lower-bound for variance of any unbiased estimator of elements of *θ* (Cramer-Rao inequality). However, for the systems we consider here the *σ*_*i*_ typically decrease very fast and there are many of them. Thus, in general, criteria based on a single number are more likely to be of less use than consideration of the set of *σ*_*i*_ as a whole.

**Fig 10:**
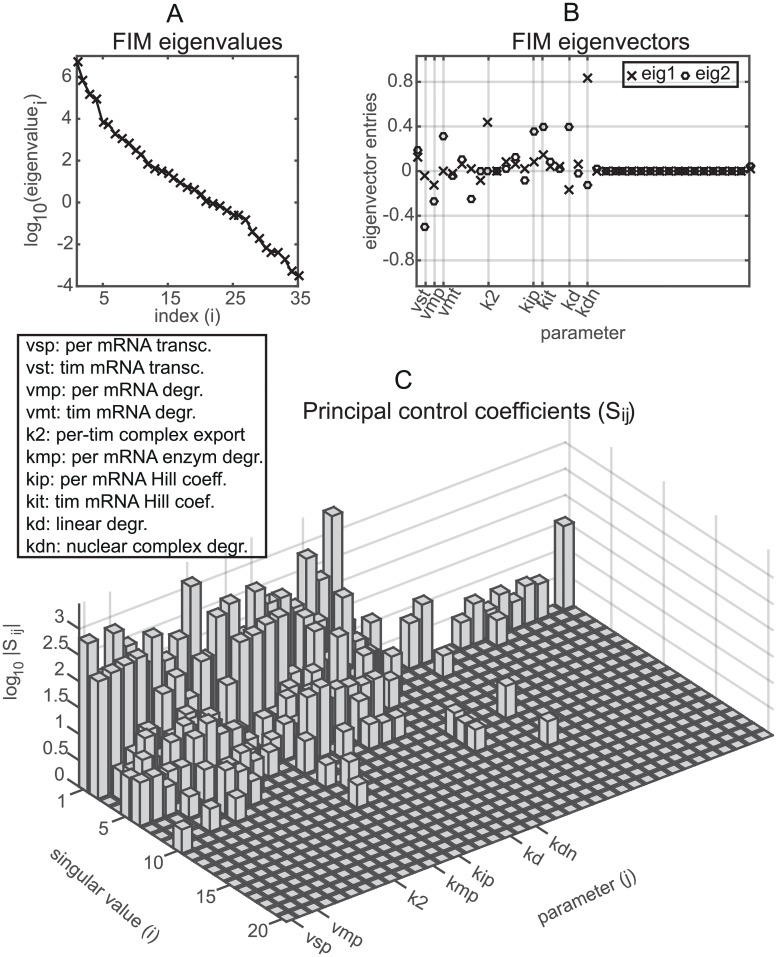
Fisher information and stochastic sensitivity analysis of the transversal distribution of the *Drosophila* circadian clock system at the times of the peaks of *per* mRNA and the nuclear complex of PER and TIM proteins. (A) The logarithm of the eigenvalues of the Fisher Information Matrix (FIM). (B) The entries/weights of the eigenvectors corresponding to the 2 largest eigenvalues of FIM (C) The largest principal control coefficients *S*_*ij*_. Small values are reduced to 0.

Calculation of the FIM for stochastic systems using the LNA has been carried out in [22] but only for small systems and short times where the LNA is accurate. It is notable that the pcLNA enables one to do such sensitivity analysis for large systems and large times. As an example, we analyse the stochastic behaviour of the *Drosophila* circadian clock based on the limit distribution $P(\underline{Q}|Q_{0})$ when

$$\begin{array}{c} \underline{Q}=Q_{x_{0}}^{\left(1\right)},Q_{x_{1}}^{\left(1\right)},Q_{x_{0}}^{\left(2\right)},Q_{x_{1}}^{\left(2\right)},\ldots ,Q_{x_{0}}^{\left(m\right)},Q_{x_{1}}^{\left(m\right)} \end{array}$$

where *x*_0_ = *g*(*t*_0_) and *x*_1_ = *g*(*t*_1_) are chosen so that *t*_0_ is the time of the peak of *per* mRNA *M*_*P*_, and *t*_1_ is the peak of the nuclear complex of PER and TIM proteins *C*_*N*_. We compute the Fisher Information of the distribution $P(\underline{Q}|Q_{0})$ using the closed form expression (S1 Sect. 9.3) for this distribution. As we can see in Fig 10(A) the eigenvalues of the Fisher Information matrix decay exponentially, with a sharp decline followed by a slower decrease. This reveals that the influential directions in the parameter space of the system are much less than its total dimension and that only a few parameters appear to be most influential. The eigenvectors associated with the two largest eigenvalues of the Fisher Information matrix (see Fig 10(B)) have large entries only for the parameters *k*_*dn*_ (PER-TIM complex nuclear degradation), *k*_*d*_ (*per* mRNA linear degradation), *k*_2_ (PER-TIM complex transportation to cytosol), *v*_*st*_ (*tim* mRNA transcription), *k*_*ip*_ (*per* mRNA Hill coefficient) and *k*_*it*_ (*tim* mRNA Hill coefficient).

The exponential decrease of the eigenvalues is typical of tightly coupled deterministic systems [25–31], but has to our knowledge not been demonstrated before for stochastic systems. It has important consequences. For example, it tells us that only a few parameters can be estimated efficiently from time-series data unless the system is perturbed in some way to get complementary data and that there will be identifiability problems that can be analysed using the FIM. It can also be used to design experiments by considering the FIM of a combination of models including one for the proposed new experiment, choosing the new experiment so as to optimally alleviate the decline of the eigenvalues.

### Sensitivity analysis for stochastic systems

The fact that we can calculate the Fisher Information allows a new approach to sensitivity analysis for stochastic systems. Anderson [23] and Srivastava et al. [24] also perform sensitivity analysis for small stochastic systems (up to 4 species and 8 reactions) in which they calculate the dependence of certain summary functions or statistics at one or more times to individual parameters. Our approach is different in that we use the fact that our distributions of interest are MVN and measure the change in the distribution of the system state at any given set of phases without recourse to any summary function and, moreover, this change is calculated for any combination of parameter variations. A major difference is our use of SVD below to find a basis of mean-covariance space using the principal components that enables us to decompose these changes into different orthogonal directions that pick out the important and unimportant directions. The approach can also be formulated for the wider class of exponential families i.e. distributions that admit a representation of the form

$$\begin{array}{c} P\left(x\right)=\exp\{C\left(x\right)+\sum_{i}\theta_{i}F_{i}\left(x\right)-\varphi \left(\theta \right)\} \end{array}$$

in terms of functions *C*, *F*_1_, …*F*_*m*_ of the state variable *x* and a function *φ* of the parameters *θ*.

We consider a family of probability distributions *P*(*X*, *θ*) which we assume are MVN with mean *μ*(*θ*) and covariance matrix Σ(*θ*) depending on the parameters *θ*. We show that there is a natural matrix of sensitivities *S*_*ij*_ associated with such a system. These are system-global in that they look at how all components of the systems change with parameters. They also have an intimate relationship with Fisher information. Note that these results are not restricted to the transversal distributions derived in previous sections but apply more generally to any MVN distribution with mean *μ* = *μ*(*θ*) and covariance matrix Σ = Σ(*θ*) parameterised by a *s*-dimensional vector *θ*, *s* ≥ 1.

As is well-known in Information Geometry, the set of multivariate normal distributions MVN^*n*^ on ℝ^*n*^ can be given the structure of a Riemannian manifold in which the Riemannian metric is given by the line element

$$\begin{array}{c} ds^{2}=d\mu^{T}\Sigma^{-1}\;d\mu +\left(1/2\right)\text{tr}\left\{{\left(\Sigma^{-1}d\Sigma \right)}^{2}\right\}. \end{array}$$

Points in MVN^*n*^ are denoted by Θ = (*μ*, Σ) where *μ* is the mean and Σ the covariance matrix. The corresponding inner product in the tangent space at Θ_0_ = (*μ*, Σ) is given by

$$\begin{array}{c} {\left\langle \delta \Theta_{1},\delta \Theta_{2}\right\rangle }_{\Theta_{0}}=\delta \mu_{1}\Sigma^{-1}\delta \mu_{2}+\frac{1}{2}\text{tr}(\Sigma^{-1}\delta \Sigma_{1}\Sigma^{-1}\delta \Sigma_{2}) \end{array}$$ (8)

where *δ*Θ_*j*_ = (*δμ*_*j*_, *δ*Σ_*j*_), *j* = 1, 2.

In calculating the FIM we have to determine the partial derivatives ∂*μ*/∂*θ*_*i*_ and ∂Σ/∂*θ*_*i*_. The derivative *M* of the mapping *θ* → (*μ*(*θ*), Σ(*θ*)) at a parameter value *θ*_0_ is given by

$$\begin{array}{c} M\cdot \delta \theta =(\sum_{i}\frac{\partial \mu }{\partial \theta_{i}}\delta \theta_{i},\sum_{i}\frac{\partial \Sigma }{\partial \theta_{i}}\delta \theta_{i}) \end{array}$$

where the derivatives are calculated at *θ*_0_.

We can regard *M* as a linear mapping between the parameter space ℝ^*s*^ and MVN^*n*^ with the inner product given in Eq (8). We can then prove (S1 Sect. 11) that we can find *s* orthonormal vectors *V*_*i*_ spanning the parameter space ℝ^*s*^, *s* orthonormal vectors *U*_*i*_ in the space MVN^*n*^ and numbers *σ*_1_ ≥ ⋯ ≥ *σ*_*s*_ ≥ 0 such that

$$\begin{array}{c} MV_{i}=\sigma_{i}U_{i},\;i=1,\ldots ,s. \end{array}$$ (9)

Note that the orthonormality of the *U*_*i*_ is with respect to the inner product 〈⋅, ⋅〉_Θ_0__. The eigenvalues of the FIM *F* are the squares of the *σ*_*i*_ because with respect to the standard inner product on *θ*-space and 〈⋅, ⋅〉_Θ_0__ on MVN^*n*^ the adjoint M* satisfies M* *M* = *F* (S1 Sect. 11).

If we let $U_{i}=\left(U_{i}^{\mu },U_{i}^{\Sigma }\right)$ denote the decomposition of *U*_*i*_ into *μ* and Σ components, then the following key property follows from Eq (9): if *δθ* is any change of parameters, the change in *μ* and Σ is given by

$$\begin{array}{cc} \delta \mu & =\sum_{i}U_{i}^{\mu }(\sum_{j}S_{ij}\delta \theta_{j})+{\text{O}(\|\delta \theta \|}^{2})\\ \delta \Sigma & =\sum_{i}U_{i}^{\Sigma }(\sum_{j}S_{ij}\delta \theta_{j})+{\text{O}(\|\delta \theta \|}^{2}) \end{array}$$ (10)

where *S*_*ij*_ = *σ*_*i*_ *V*_*ji*_.

One can define other sensitivities in a similar way but using a different orthogonal basis of MVN^*n*^, but the above *S*_*ij*_ satisfy an important optimality condition explained in S1 Sect. 11 which asserts that the basis *U*_*i*_ and the corresponding sensitivities *S*_*ij*_ are optimal for capturing as much sensitivity as possible in the low order principal components *U*_*i*_.

In view of this we call the *S*_*ij*_ the *principal control coefficients*. Note that the role of the *S*_*ij*_ as sensitivities is seen from the following relation which follows from Eq (10) (where *S* = (*S*_*ij*_)),

$$\begin{array}{c} ∥\delta \Theta ∥=∥S\cdot \delta \theta ∥+O(∥\delta \theta {∥}^{2}). \end{array}$$ (11)

These sensitivities are relatively easy to calculate using the information in S1 Sect. 11. In Fig 10(C) we show the *S*_*ij*_ for the transversal distribution of the *Drosophila* circadian clock at the times of the peak of *per* mRNA and the peak of the nuclear complex of PER and TIM proteins. As we can see, because *S*_*ij*_ = *σ*_*i*_ *V*_*ji*_ the coefficients rapidly decrease with the singular values *σ*_*i*_, while a few parameters, similar to those with large eigenvector entries, have high coefficients.

### Calculating likelihoods via a pcLNA Kalman Filter

The likelihood function of a set of time-series observations of a system can be used for parameter estimation, hypothesis testing and other forms of statistical inference. For example, one may wishes to use the likelihood function to estimate parameters of a biological system. Although there is no elegant formula for

$$\begin{array}{c} P\left(\underline{X}|X\left(t_{0}\right)\right)=P\left(X\left(t_{1}\right),\ldots ,X\left(t_{m}\right)|X\left(t_{0}\right)\right) \end{array}$$

similar to that for $P(\underline{Q}|X\left(t_{0}\right))$ above, we can efficiently calculate it. To do this we derive a Kalman Filter for the pcLNA that is a modification of the Kalman Filter associated with the LNA [35]. This can be used to compute the likelihood function $L(\theta ;\hat{\underline{X}})$ of the system parameters *θ* with respect to observations $\hat{\underline{X}}=(\hat{X}\left(t_{0}\right),\hat{X}\left(t_{1}\right),\ldots ,\hat{X}\left(t_{N}\right))$ recorded at *N* times *t*_0_, *t*_1_, …, *t*_*N*_ that are noisy linear functions of the species concentrations. This is slightly more general than just calculating $P(\underline{X}|X\left(t_{0}\right))$ because we allow for a measurement equation. The Kalman filter can also be used for forward prediction.

We assume the measurement equation,

$$\begin{array}{c} \hat{X}\left(t\right)=BX\left(t\right)+\epsilon , \end{array}$$ (12)

relating the observations $\hat{X}\left(t\right)$ to the state variables, *X*(*t*). Here *B* is a transformation matrix (often simply removing unobserved species or introducing unknown scalings) and *ϵ* = (*ϵ*_1_, …, *ϵ*_*n*_)∼*MVN*(0, Σ_*ϵ*_) the observational error. The pcLNA likelihood can be decomposed as the product

$$\begin{array}{c} L\left(\theta ;\hat{\underline{X}}\right)=P\left(\hat{X}\left(t_{0}\right);\theta \right)\;\prod_{i=1}^{n}P\left(\hat{X}\left(t_{i}\right)|\hat{X}\left(t_{i-1}\right);\theta \right). \end{array}$$

The pcLNA Kalman Filter algorithm, which we describe in more detail in S1 Sect. 15, uses a recursive algorithm for computing the terms in $L(\theta ;\hat{\underline{X}})$. The algorithm proceeds by iteration *i* = 1, 2, … and uses Bayes rule to derive the posterior distributions of $\left(X\left(t_{i-1}\right)|\hat{X}\left(t_{i-1}\right)\right)$ and a phase correction to obtain *g*(*s*_*i* − 1_) = *G_N_*(μ*(*t*_*i* − 1_)) and the corrected noise distribution of $(\kappa \left(s_{i-1}\right)|\hat{X}\left(t_{i-1}\right)).$ The LNA transition equation (S1 Eq. (4.2)) is then used to derive the distribution of $\left(\xi \left(t_{i}\right)|\hat{X}\left(t_{i-1}\right)\right)$ and the LNA ansantz Eq (3) to obtain $(X\left(t_{i}\right)|\hat{X}\left(t_{i-1}\right)).$ The measurement equation Eq (12) is finally used to obtain the (*i* + 1)th term of the likelihood function $P(\hat{X}\left(t_{i}\right)|\hat{X}\left(t_{i-1}\right))$ before proceeding to the next iteration. All the distributions obtained in this way are MVN with easily computable parameter values.

If the observations are recorded in short time intervals, the phase correction can be omitted in some steps, in which case the algorithm proceeds as in [35]. Computational methods such as those described in [32] and [35] can then be used to perform likelihood-based statistical inference.

## Methods

All computations have been carried out using MATLAB Release 2016b, The MathWorks, Inc., Natick, MA, USA. In particular, the empirical CDF plots, (q-q) plots, histograms, smooth probability density functions and KS distances are derived using the ecdf, qqplot, histogram, ksdensity, kstest functions of MATLAB and Statistics Toolbox. The computations for the SSA, tau-leap, integration of diffusion and pcLNA simulation algorithms, and the computation of Fisher Information and principal control coefficients for the sensitivity analysis were performed using the PeTTSy software which is discussed in S1 Sect. 14 and is freely available at http://www2.warwick.ac.uk/fac/sci/systemsbiology/research/software/. Further details concerning methods are given in S1 Sect. 16.

## Discussion

We present a comprehensive treatment of stochastic modelling for large stochastic oscillatory systems. Practical algorithms for fast long-term simulation and likelihood-based statistical inference are provided along with the essential tools for a more analytical study of such systems.

There is considerable scope for future work in various directions. We expect that these results can be extended to a broader class of systems including those that are chaotic in the Ω → ∞ limit. Our approach should provide the opportunity to develop new methodology for parameter estimation, likelihood-based inference and experimental design in such systems. Finally, there is currently much interest in information transfer and decision-making in signaling systems and our methods provide new tools with which to tackle problems in this area.

If system biologists are to reliably use complex stochastic models to provide robust understanding it is crucial that there are analytical tools to enable a rigorous assessment of the quality and selection of these models and their fit to current biological knowledge and data. Our aim in this paper is to contribute to that but the results should be of much broader interest.

## Supporting information

S1 Appendix Technical details. In this note we give further details about the mathematical underpinnings of the pcLNA methods discussed in the main paper.
(PDF)

S2 Appendix *Drosophila* circadian clock system. In this note we give details about the *Drosophila* circadian clock and use this system to illustrate further the accuracy of distributions and simulations discussed in the main paper.
(PDF)

S3 Appendix Brusselator and NF-*κ*B systems. In this note we give details about the deterministic and stochastic models of the Brusselator and NF-*κ*B systems and use them to illustrate further the results described in the main paper.
(PDF)
