# Supplementary material for: Long-time analytic approximation of large stochastic oscillators: Simulation, analysis and inference
Source: PLoS Comput Biol. 2017 Jul 24;13(7):e1005676. doi: 10.1371/journal.pcbi.1005676 (PMC5555717; doi:10.1371/journal.pcbi.1005676)
Supplement: S1 Appendix — In this note we give further details about the mathematical underpinnings of the pcLNA methods discussed in the main paper. (PDF) [file pcbi.1005676.s001.pdf]

Supplementary information S1 for the paper Long-time analytic approximation of large stochastic oscillators: simulation, analysis and inference

Giorgos Minas[1,2] & David A Rand[1,2]

[1] Zeeman Institute for Systems Biology & Infectious Disease Epidemiology Research & [2]Mathematics Institute,

University of Warwick, Coventry CV4 7AL, UK

July 14, 2017

Contents

1 Mathematical details **1**

2 The system size parameter Ω **3**

3 Derivation of the classical deterministic equation **3**

4 The Linear Noise Approximation (LNA) **4**

5 The LNA transversal distribution at a fixed time **5**

6 Distribution of phase fluctuations δt and $g(t) - G_N(X(t))$ **6**

7 The approximation of the LNA transversal distributions **8**

8 Proof of convergence for LNA transversal distributions **9**

8.1 Free-running oscillators. 9

8.2 Extending this result to arbitrary transversals. 11

8.3 Alternative geometrical proof that convergence on $\mathcal{S}_N$ implies convergence on other transversals $\mathcal{S}$. 11

8.4 Entrained forced oscillators. 11

9 Analytic expression for pcLNA transversal distributions **12**

9.1 Fixed point distribution. 13

9.2 The joint distribution of multiple transversals 13

9.3 Precision matrix for $P(\underline{Q}|X(t_0))$. 14

10 Other transversal sections **14**

11 Details behind the sensitivity analysis. **15**

11.1 Deducing equation (S11.4) from SVD. 17

12 Multiple crossings in each rotation **17**

13 Negative populations **21**

14 PeTTSy **21**

15 The pcLNA Kalman Filter **23**

16 Details related to various computations **24**

Abstract

We refer to the paper "Long-time analytic approximation of large stochastic oscillators: simulation, analysis and inference" by **I**. In this note we give further details about the mathematical underpinnings of the pcLNA methods discussed in **I**.

1 Mathematical details

When we refer to a function, mapping or vectorfield as being smooth we mean that it has C^2 dependence on the relevant variables. We are assuming throughout that the vector field F introduced in **I** is C^2 and that the limit cycle γ given by $g(t)$ is a stable elementary limit cycle in the sense we describe now. Here $x \in \mathbb{R}^n$.

If $g(t)$ is the periodic solution of interest (with period τ), consider the linear differential equation $dC/dt = J(t)\, C$ where $J(t)$ is the $n \times n$ Jacobian matrix whose ijth entry is $\partial F_i/\partial x_j$ evaluated at $g(t)$. Let $C(s,t)$ be the solution of this equation with initial condition $C(s,s) = I_n$ the identity $n \times n$ matrix. If $C(t) = C(s,t)$ then $C(t)$ has a representation of the form $C(t) = Z(t)e^{R(t-s)}$ where $Z(t+\tau) = Z(t)e^{R\tau}$ (Theorem 6.1 of Chapter IV [5]). If $\lambda_1, \ldots, \lambda_n$ are the eigenvalues of R then $e^{t\lambda_1}, \ldots, e^{t\lambda_1}$ are the eigenvalues of e^{Rt}. As such they are only determined modulo $2\pi i/\tau$. Modulo this indeterminacy, they are called the *characteristic exponents* of the limit cycle. The characteristic exponents are independent of the choice of the initial time s.

We assume that all limit cycles γ considered in this paper are *elementary and stable* in the following sense. In the case of a limit cycle γ of a free-running oscillator (i.e. when F does not depend directly upon t), we say that γ is *elementary* if $n-1$ of the characteristic exponents have non-zero real parts and the remaining characteristic exponent is zero (modulo $2\pi i/\tau$). Such limit cycles are often called *hyperbolic.* If in addition the $n-1$ non-zero characteristic exponents have negative real part then we say it is *stable and elementary.*

In the case of periodically forced oscillators the limiting differential equation is $\dot{x} = F(t, x)$ and, in this case γ is elementary if all the characteristic exponents have non-zero real parts and it is stable if all have negative real parts.

A number of desirable properties flow from these conditions. Firstly, for elementary and stable limit cycles there is a neighbourhood of γ in $\mathbb{R}^n$ such that any solution of $\dot{x} = F(t, x)$ with its initial condition in the neighbourhood converges exponentially fast to γ (Theorem 11.1. Chap. IX [5]). Secondly, suppose F depends smoothly on parameters θ so that $F = F_\theta$. Then if $g_{\theta_0}(t)$ is a elementary limit cycle for $F = F_{\theta_0}$, there is a unique family g_θ defined for θ near θ_0 which depends smoothly upon θ such that g_θ is an elementary limit cycle of $F = F_\theta$ (Theorem 2.3, Chap. XII [5]).

Almost all the results described in the main paper that are for normal transversals $N(x)$ hold for arbitrary transversals $\mathcal{S}_x$. By a transversal section through $x \in \gamma$ we mean a $(n-1)$-dimensional linear hyperplane $\mathcal{S}_x$ containing x and transversal to the tangent vector, $F(x)$, to γ at x. A particular example is the hyperplane normal to γ at x. A transversal system is a family $\mathcal{S}_{g(t)}$ of transversal sections which vary smoothly with t in the sense that the unit normal vector to $\mathcal{S}_{g(t)}$ varies smoothly with t. Throughout this document we consider this more general situation.

A transversal system defines a mapping G of a neighborhood of γ onto γ where if $X \in \mathcal{S}_x$ then $G(X) = x \in \gamma$. In cases where $X(s)$ lies in more than one transversal sections, $\mathcal{S}_{x(t')}$, $t' = t_1, t_2, \ldots$, then $G(X(s)) = x(t)$ with $t = \min_i |t_i - s|$ the closest time to s. We denote this mapping for the normal transversal system by G_N.

An adapted coordinate system $\mathcal{C}_{g(t)}$ at a point $g(t)$ on γ is one determined by a set of orthonormal basis vectors $e_1(t), \ldots, e_n(t)$ with $e_1(t)$ the unit normal vector to $\mathcal{S}_{g(t)}$ and the vectors $e_2(t), \ldots, e_n(t)$ forming an orthonormal basis of $\mathcal{S}_{g(t)}$. If these are defined for t in some interval in $\mathbb{R}$ then we always assume that the $e_i(t)$ have smooth (i.e. C^2) dependence upon t. It is important that the coordinates are defined by an orthonormal basis in the original coordinates because this effectively preserves the covariance matrix in the sense that a covariance matrix V in the adapted coordinates is UVU^T in the original coordinates with U a real orthogonal

matrix. In particular, the eigenvalues are preserved.

2 The system size parameter Ω

It is very common to consider stochastic systems involving a system size parameter Ω which is a parameter that occurs in the intensities of the reactions $w_j(Y(t))$ [8, 9]. The precise description of this parameter depends on the system and it governs the size of the fluctuations and therefore the size of the jumps. In population models it might be considered to be of the same order of magnitude as the total population size while in chemical systems a natural choice is to use molar concentrations and therefore regard Ω as Avogadro's number in the appropriate molar units (e.g. nM^{-1}) multiplied by the volume of the reacting solution in appropriate units (e.g. in litres (L)). In the *Drosophila* circadian clock model we consider it has units L/nM.

It can be interpreted as being the same order of magnitude as the mean size of the molecular populations. When the system and the systems size parameter satisfy the LNA Ansatz $Y(t)/\Omega = x(t) + \xi(t)/\sqrt{\Omega}$ (see Sect. S4) then $Y(t) = \mathrm{O}(\Omega)$ and the fluctuations are $\mathrm{O}(\sqrt{\Omega})$.

In our examples, the rates $w_j(Y(t))$ depend upon Ω as $w_j(Y) = \Omega u_j(Y/\Omega)$ as used in the proof by Kurtz for the convergence of the LNA as $\Omega \to \infty$ [8]. For example, Hill functions in our rate equations have the form $w_H = c\Omega N^h/(N^h + (k\Omega)^h)$ where N stands for the relevant population numbers and c and k are parameters. Therefore, if $n = N/\Omega$, $w_H = c\Omega n^h/(n^h + k^h)$. Similarly, degradation and binding reaction rates have the form $w_{\mathrm{deg}} = cN$ and $w_{\mathrm{bind}} = cN_1N_2/\Omega$ so that $w_{\mathrm{deg}} = c\Omega n$ and $w_{\mathrm{bind}} = c\Omega n_1 n_2$.

In a certain sense the system size parameter is just a mathematical convenience to enable the study of the dependence of stochastic fluctuations upon system size. Indeed, the methods we develop in this paper can and should be applied to systems that do not involve a system size parameter but then it will be necessary to ensure that the population sizes achieved in the given system are large enough.

3 Derivation of the classical deterministic equation

We provide a derivation of the macroscopic law of mass action that is similar to [1].

The state of the system at some time t is

$$Y(t) = Y(0) + \sum_j \nu_j Z_j \left(\int_0^t w_j(Y(s))ds \right)$$

with Z_j independent unit Poisson processes corresponding to the j-th reaction channel. The

latter equation can be written in terms of $X = Y/\Omega$ as

$$X(t) = X(0) + \sum_j \nu_j \Omega^{-1} Z_j \left(\int_0^t \Omega u_j(X(s)) ds \right),$$

since $w_j(Y) = \Omega u_j(X)$ (see Section S2). Using the law of large numbers, as $\Omega \to \infty$, we have that

$$x(t) = x(0) + \sum_j \nu_j \int_0^t u_j(x(s)) ds,$$

where here $x(t)$ is the limit of $X(t)$ for $\Omega \to \infty$. Consequently, $x(t)$ satisfies the ordinary differential equation (ODE)

$$\dot{x} = \sum_j \nu_j u_j(x). \tag{S3.1}$$

4 The Linear Noise Approximation (LNA)

The LNA is based on the Ansatz

$$X(t) = \frac{Y(t)}{\Omega} = x(t) + \frac{\xi(t)}{\sqrt{\Omega}} \tag{S4.1}$$

where $x(t)$ is a solution of the limiting $\Omega \to \infty$ system in Eq. (S3.1) which we assume is a stable limit cycle of minimal period $\tau > 0$ given by $x = g(t)$, $0 \le t \le \tau$.

Recall the definition of the $n \times n$ matrices $C(s,t)$ introduced in Section S1. In the LNA, the stochastic variable ξ in (S4.1) satisfies

$$\xi(t) = C(t_0, t)\xi(t_0) + \eta(t_0, t), \qquad t_0 < t, \tag{S4.2}$$

where $\eta(t_0, t) \sim \mathrm{MVN}(0, V(t_0, t))$ is multivariate normal with mean 0 and covariance matrix

$$V(t_0, t) = \int_{t_0}^t C(s,t)E(s)E(s)^T C(s,t)^T ds \tag{S4.3}$$

and $E(s)$ the matrix product of the stoichiometry matrix (i.e. the matrix whose columns are the vectors ν_j introduced in **I**) and the square root of the diagonal matrix with main diagonal the reaction rates $u_j(x(s))$. The noise $\xi(t)$ also satisfies the Stochastic Differential Equation,

$$d\xi(t) = J(g(t))\xi(t)dt + E(s)dW(t),$$

where $W(t)$ is a K-dimensional Brownian motion (K: the number of possible reactions). Equation (S4.3) follows from this by using Itô's change of variable formula and the Itô Isometry.

The proof of convergence as $\Omega \to \infty$ of the true distribution of ξ to a multivariate normal distribution with mean and covariance matrix given by (S4.2) and (S4.3) is contained in Chap. 8 of [8].

Henceforth, we write $\zeta \sim \mathrm{MVN}(m, S)$ to mean that a random variable ζ is multivariate normal with mean m and covariance matrix S.

5 The LNA transversal distribution at a fixed time

We now suppose that our initial condition $\xi(t_0)$ is drawn from a MVN distribution P_0 which is supported on the transversal section $S_{g(t_0)}$. This is denoted $\xi(t_0) \sim P_0$. For $t_0 < t_1$, we consider the distribution, $P_{\text{LNA}}(X(t_1)|X(t_1) \in \mathcal{S}_{g(t_1)}, \xi(t_0) \sim P_0)$, of $X(t_1)$ conditional on $X(t_1)$ lying on the transversal sections $\mathcal{S}_{g(t_1)}$ and $\xi(t_0) \sim P_0$.

We have adapted coordinate systems $(y_1, \mathbf{y}_2)$ at $x_1 = g(t_1)$ so that $\mathcal{S}_{x_1}$ is given by $y_1 = 0$. We write the matrices $C(t_0, t_1)$ and $V(t_0, t_1)$ in the latter coordinate system as

$$C(t_0, t_1) = \begin{bmatrix} C_{11} & C_{12} \\ \\ C_{21} & C_{22} \end{bmatrix} \qquad \text{and} \qquad V(t_0, t_1) = \begin{bmatrix} V_{11} & V_{12} \\ \\ V_{21} & V_{22} \end{bmatrix}$$

where $V_{12} = V_{21}^T$. By $X(t_0) \in \mathcal{S}_{x_0}$ we have that $\xi(t_0) = \kappa_0$. Then, under the LNA,

$$\xi(t_1) = (\xi_1(t_1), \xi_{\mathbf{2}}(t_1))^T = C(t_0, t_1) \cdot \xi(t_0) + \eta = (C_{1\cdot}\kappa_0, C_{2\cdot}\kappa_0)^T + \eta$$

where $C_{1\cdot} = [C_{11} \;\; C_{12}]$, $C_{2\cdot} = [C_{21} \;\; C_{22}]$ and $\eta \sim \mathrm{MVN}(0, V(t_0, t_1))$. Consequently, $\xi_{\mathbf{2}}(t_1)$ conditional on $\xi_1(t_1) = 0$ is MVN with mean $\check{C}(t_0, t_1)\kappa_0$ and covariance matrix $\check{V}(t_0, t)$, where $\check{C}(t_0, t_1) = C_{2\cdot} - V_{21}C_{1\cdot}/V_{11}$ and $\check{V}(t_0, t_1) = V_{22} - V_{21}V_{12}/V_{11}$. Thus if $\kappa_0 \sim \mathrm{MVN}(m_0, S_0)$ and $\kappa(t_1)$ denotes $\xi_{\mathbf{2}}(t_1)$ conditional on $\xi_1(t_1) = 0$, then $\kappa(t_1)$ is MVN with mean and covariance matrix respectively given by

$$m_1 = \mathrm{E}(\kappa(t_1)) = \check{C}(t_0, t_1) \cdot m_0, \quad S_1 = \mathrm{Var}(\kappa(t_1)) = \check{C}(t_0, t_1) S_0 \check{C}(t_0, t_1)^T + \check{V}(t_0, t_1) \quad \text{(S5.1)}$$

and $P_{\text{LNA}}(X(t_1)|X(t_1) \in \mathcal{S}_{x_1}, \xi(t_0) \sim MVN(m_0, S_0))$ is $MVN(\mu_1, \Sigma_1)$ with $\mu_1 = \Omega^{-1/2} m_1$ and $\Sigma_1 = \Omega^{-1} S_1$.

6 Distribution of phase fluctuations δt and $g(t) - G_N(X(t))$

In Sect. "Stochastic fluctuations in periods and timing" of **I**, we consider the distribution of the fluctuation δt in the time taken for the lifted phase of a stochastic trajectory to go from a given phase ϕ_1 to a greater one ϕ_2. We showed that for moves from $\phi_1 = 0$ and $\phi_2 = 2\pi q$ for $q = 1, \ldots, 8$ the distribution of the fluctuations is approximately normal with variance increasing with q and that for $\phi_1 = 2(q-1)\pi$ and $\phi_2 = 2q\pi$ for $q = 1, \ldots, 8$ the distributions are again approximately normal with approximately constant variance. We now consider an approximation of the distribution of the fluctuations under the LNA.

In this and the next section we will be considering how to approximate the distribution of first intersections with $\mathcal{S}_{g(t_1)}$ of stochastic trajectories $X(t)$ that start in $\mathcal{S}_{g(t_0)}$. To do this we will consider LNA trajectories with initial condition a given MVN distribution P_0 on $\mathcal{S}_{g(t_0)}$ and their first intersection at time $t = t_1 + \delta t$ with $\mathcal{S}_{g(t_1)}$. In this section we show that the distribution of the variation in time, δt, is approximately normal and calculate approximations of the mean and variance. Thus, we consider the distribution under the LNA of $\xi(t)$ such that $X(t) = g(t) + \Omega^{-1/2}\xi(t) \in \mathcal{S}_{g(t_1)}$ for $t_0 \leq t < t_1 + \tau/2$. We denote this distribution by $P_{\text{LNA}}(\xi(t)|X(t) \in \mathcal{S}_{g(t_1)})$.

To calculate the statistics of the variation in time, δt, for the distribution of the rth intersection with $\mathcal{S}_{g(t_1)}$ of stochastic trajectories $X(t)$ that start in $\mathcal{S}_{g(t_0)}$ one decomposes the trajectory into the part from $\mathcal{S}_{g(t_0)}$ to its first intersection with $\mathcal{S}_{g(t_1)}$ and then the $(r-1)$ passes from $\mathcal{S}_{g(t_1)}$ to itself and uses the fact that the total variation is the sum of the individual ones which are independent random variables. Since we show below that the individual variation are approximately normal it follows that the total is. Therefore in this section we now focus just on first intersections.

We assume that the initial conditions $\xi(t_0)$ have a MVN distribution P_0 on $\mathcal{S}_{g(t_0)}$ with mean 0 and covariance matrix V_0. We fix adapted coordinate systems at each $g(t)$. Then $\xi(t_1 + \delta t)$ has mean 0 and covariance matrix $\check{V}(t_0, t_1 + \delta t) = C(t_0, t_1 + \delta t)V_0 C(t_0, t_1 + \delta t)^T + V(t_0, t_1 + \delta t)$ where $V(t_0, t_1 + \delta t)$ is given by (S4.3).

If $\xi(t_1 + \delta t)$ is such that $X(t_1 + \delta t) \in \mathcal{S}_{g(t_1)}$ then $G(X(t_1 + \delta t)) = g(t_1)$ and therefore $\kappa(t_1 + \delta t) = \xi(t_1 + \delta t) + \sqrt{\Omega}(g(t_1 + \delta t) - g(t_1))$. If $\underline{n}(t)$ denotes the unit vector $F(g(t))/||F(g(t))||$, since $\kappa(t_1 + \delta t)$ is orthogonal to $\underline{n}(t_1)$, we have that

$$\underline{n}(t_1) \cdot \xi(t_1 + \delta t) + \sqrt{\Omega}\, \underline{n}(t_1) \cdot (g(t_1 + \delta t) - g(t_1)) = 0 \tag{S6.1}$$

But since $\dot{g}(t) = F(g(t))$ and the time derivative of $F(g(t))$ is $J(g(t)) \cdot F(g(t))$ it follows that,

up to terms that are $\mathrm{O}(|\delta t|^3)$,

$$g(t_1+\delta t)-g(t_1)=F(t_1)\delta t+(1/2)J(g(t_1))\cdot F(g(t_1))(\delta t)^2. \tag{S6.2}$$

Now choose adapted coordinates $(x_1,\ldots,x_n)$ at $g(t_1)$ so that $F(g(t_1))=(\alpha,0,\ldots,0)$ and $\underline{n}(t_1)=(1,0,\ldots,0)$. Then, $\underline{n}(t_1)\cdot(J(g(t_1))\cdot F(g(t_1)))=\alpha J_{11}$ where J_{11} is the first entry of $J(g(t_1))$. Thus using (S6.1),

$$\frac{\underline{n}(t_1)\cdot\xi(t_1+\delta t)}{\sqrt{\Omega}}+\alpha\delta t+\frac{1}{2}\alpha J_{11}\delta t^2=\mathrm{O}(|\delta t|^3).$$

Since all terms but one are independent of Ω and since $\xi(t_1+\delta t)$ has finite variance, it follows that the standard deviation of δt is $\mathrm{O}(\Omega^{-1/2})$. Thus the rhs η is a random variable depending stochastically only on δt which is independent of Ω and has a standard deviation and mean that is $\mathrm{O}(\Omega^{-3/2})$. Letting $\kappa=\alpha J_{11}/2$ and $\Xi=\left(\underline{n}(t_1)\cdot\xi(t_1+\delta t)\right)/\sqrt{\Omega}$, completing the square of the lhs gives $\delta t+\alpha/2\kappa=\pm\sqrt{1-4\kappa(\Xi-\eta)/\alpha^2}$ where η is the above $\mathrm{O}(|\delta t|^3)$ term. Using $\sqrt{1+x}=1+x/2-x^2/8+\mathrm{O}(|x|^3)$ and ignoring the negative root gives,

$$\delta t=-\frac{1}{\alpha}\Xi-\frac{J_{11}}{2\alpha^2}\Xi^2. \tag{S6.3}$$

Note that $\underline{n}(t_1)\cdot\xi(t_1+\delta t)$ differs from $\underline{n}(t_1)\cdot\xi(t_1)$ by an amount that is $\mathrm{O}(|\delta t|^2)$ (since the base point moves a distance that is $\mathrm{O}(|\delta t|)$ around the limit cycle) and the mean of $\xi(t_1+\delta t)$ given δt is $C(t_0,t_1+\delta t)\,E_{P_0}(\xi(t_0))$ which is 0 if the mean $E_{P_0}(\xi(t_0))$ of P_0 is 0. Thus, the mean of Ξ is 0 and, up to an error that is $\mathrm{O}(\Omega^{-3/2})$, the mean of the last term in (S6.3) is $(J_{11}/2\alpha^2)\mathrm{Var}(\Xi)$ which is $\mathrm{O}(\Omega^{-1})$ and the variance of this term is $\mathrm{O}(\Omega^{-2})$.

The variance of δt is,

$$\begin{aligned}\mathrm{Var}(\delta t)&=(1/\alpha^2)\mathrm{Var}(\Xi)+\mathrm{O}(\Omega^{-2})\\&=(1/\alpha^2\Omega)\,\mathrm{Var}(\underline{n}(t_1)\cdot\xi(t_1+\delta t))+\mathrm{O}(\Omega^{-2})\\&=(1/\alpha^2\Omega)\,\underline{n}(t_1)^T\check{V}(t_0,t_1+\delta t)\underline{n}(t_1)+\mathrm{O}(\Omega^{-2})\\&\overset{(1)}{=}(1/\alpha^2\Omega)\,\underline{n}(t_1)^T\check{V}(t_0,t_1)\underline{n}(t_1)+\mathrm{O}(\Omega^{-3/2})\\&=(1/\alpha^2\Omega)\,\check{V}_{11}+\mathrm{O}(\Omega^{-3/2})\end{aligned} \tag{S6.4}$$

where $\check{V}_{11}$ is the upper left entry of the covariance matrix $\check{V}(t_0,t_1)$ written in the given adapted coordinates. The equality marked $\overset{(1)}{=}$ follows from the fact that $\check{V}(t_0,t_1+\delta t)=\check{V}(t_0,t_1)+\mathrm{O}(|\delta t|)$.

Since Ξ in (S6.3) has a MVN distribution, it follows that δt is approximately normal but is

biased by the $(J_{11}/2\alpha^2)\Xi^2$ term which is $\mathrm{O}(\Omega^{-1})$ and hence δt has mean $\mathrm{O}(\Omega^{-1})$ and variance $(1/\alpha^2\Omega)\, V_{11} + \mathrm{O}(\Omega^{-3/2})$. For the *Drosophila* clock and system sizes $\Omega = 300$, 500 and 1000, the standard deviation of δt for completing one round of the limit cycle under the above LNA formula is respectively 0.7218, 0.5591 and 0.3954 whereas the empirical standard deviation of δt derived from the SSA is 0.7333, 0.5490 and 0.3862.

7 The approximation of the LNA transversal distributions

Recall the definition of $P_{\text{LNA}}(\xi(t)|X(t) \in \mathcal{S}_{g(t_1)})$ in Sect. S6. In this section we consider how well this is approximated by $P_{\text{LNA}}(\xi(t_1)|X(t_1) \in \mathcal{S}_{g(t_1)})$ i.e. by constraining time t to be the fixed time t_1. We show that the variance $\mathrm{Var}_{\delta t}(X(t_1+\delta t))$ of $X(t) = X(t_1+\delta t)$ conditional on $X(t) \in \mathcal{S}_{g(t_1)}$ equals the variance of $X(t_1)$ conditional on $X(t_1) \in \mathcal{S}_{g(t_1)}$ up to a term that is $\mathrm{O}(\Omega^{-1})$. The latter conditional variance is relatively easily calculated using the results in Sect. S5.

Throughout this section for a given $\xi(t)$, $X(t)$ denotes $g(t) + \Omega^{-1/2}\xi(t) \in \mathcal{S}_{g(t_1)}$. We first consider the variance of $X(t_1+\delta t)$ conditional on a fixed value of δt and $X(t_1+\delta t) \in \mathcal{S}_{g(t_1)}$. We omit the conditioning on $X(t_1+\delta t) \in \mathcal{S}_{g(t_1)}$ from the notation in the rest of this section.

The corresponding covariance matrix $\mathrm{Var}(X(t_1+\delta t)|\delta t)$ has a Taylor expansion

$$\mathrm{Var}(X(t_1+\delta t)|\delta t) = \mathrm{Var}(X(t_1)|\delta t = 0) + \delta t W + \mathrm{O}(\delta t^2)$$

where the symmetric matrix W is easily calculated.

Note that $E_{\delta t}\left[\delta t W\right] = \mathrm{O}(\Omega^{-1})$ since the mean of δt has this order (see Sect. S6). Consequently,

$$E_{\delta t}\left[\mathrm{Var}(X(t_1+\delta t)|\delta t)\right] = \mathrm{Var}(X(t_1)) + \mathrm{O}(\Omega^{-1}).$$

We now consider $\mathrm{Var}_{\delta t}(E(X(t_1+\delta t)|\delta t))$. The mean $E(X(t_1+\delta t)|\delta t) = g(t_1+\delta t)$ and therefore from (S6.2) and (S6.4), $\mathrm{Var}_{\delta t}(E(X(t_1+\delta t)|\delta t)) = O(\Omega^{-1})$. Hence we have the result because, by the Law of Total Variation,

$$\mathrm{Var}(X(t_1+\delta t)) = E(\mathrm{Var}(X(t_1+\delta t)|\delta t) + \mathrm{Var}(E(X(t_1+\delta t)|\delta t)) = \mathrm{Var}(X(t_1)) + \mathrm{O}(\Omega^{-1}).$$

8 Proof of convergence for LNA transversal distributions

8.1 Free-running oscillators.

We show that for any transversal $\mathcal{S} = \mathcal{S}_{g(t)}$ to γ there is a unique MVN distribution $P_{\mathcal{S}}^{(\mathrm{fp})}$ on $\mathcal{S}$ with the property that under the pcLNA, $P_{\text{LNA}}(Q_{x_i}^{(2)}|Q_{x_i}^{(1)} \sim P_{\mathcal{S}}^{(\mathrm{fp})}) = P_{\mathcal{S}}^{(\mathrm{fp})}$. To do this we study the limit of $P_{\text{LNA}}(Q_{x_i}^{(N)}|Q_{x_i}^{(1)})$ as $N \to \infty$ because this is such a fixed point.

We start by considering the case where $\mathcal{S}$ is the normal hypersection and fix adapted coordinates $(x_1, \mathbf{x}_2)$ in which the transversal section $\mathcal{S}$ is given by $x_1 = 0$.

By periodicity the covariance matrix of $P_{\text{LNA}}(\xi(t+n\tau)|X(0) = g(t)) = P_{\text{LNA}}(\xi(0)|X(t - n\tau) = g(t))$ is $V(t - N\tau, t)$ (see (S4.3)). We have,

$$\begin{aligned} V(t-N\tau,t) &= \int_{t-N\tau}^{t} C(s,t)E(s)E(s)^T C(s,t)^T\, ds \\ &= \sum_{k=0}^{N-1} C(t-k\tau,t)V(t,t+\tau)C(t-k\tau,t)^T \\ &= \sum_{k=0}^{N-1} C^k V (C^k)^T = \sum_{k=0}^{N-1} (C^k L)(C^k L)^T \end{aligned} \tag{S8.1}$$

where $C = C(t, t+\tau)$, $V = V(t, t+\tau) = \int_t^{t+\tau} C(s,t)E(s)E(s)^T C(s,t)^T\, ds$ and $V = LL^T$ is the Cholesky decomposition of the positive definite matrix V.

In what follows we utilise the vec operator and the Kronecker product $\otimes$. All the facts about these that are used here can be found in [7] Chapter 4. A basic equality is that $\text{vec}(AXB) = (B^T \otimes A)\text{vec}(X)$. Therefore, since $\text{vec}(C^k V (C^k)^T) = (C^k \otimes C^k)\text{vec}(V) = (C \otimes C)^k \text{vec}(V)$,

$$\text{vec}(V(t-N\tau,t)) = \sum_{k=0}^{N-1} (C \otimes C)^k \text{vec}(V). \tag{S8.2}$$

Our adapted coordinates $(x_1, \mathbf{x}_2) = (x_1, x_1, \ldots, x_n)$ at $g(t)$ are determined by the the unit vectors $e_i(t)$ (see Sect. S5) where $e_1(t) = F(g(t))/||F(g(t))||$ tangent to the limit cycle. Since $C\, e_1(t) = e_1(t)$ it follows that

$$C^k = \begin{bmatrix} 1 & \alpha^T \\ 0 & A \end{bmatrix}^k = \begin{bmatrix} 1 & b^{(k)} \\ 0 & A^k \end{bmatrix}$$

where A is an $(n-1) \times (n-1)$ matrix, α^T is a $n \times 1$ vector and $b^{(k)} = \alpha^T + \alpha^T A \cdots + \alpha^T A^{k-1}$.

Since γ is elementary and stable, 1 is a simple eigenvalue of C, and therefore all eigenvalues λ of A have $|\lambda| < 1$ and therefore $b^{(k)}$ converges to $b^{(\infty)} = \alpha^T (I - A)^{-1}$. Moreover, all eigenvalues $\lambda \neq 1$ of C^k (i.e. all eigenvalues of A^k) have modulus $|\lambda| < c\nu^k$ where $0 < \nu < 1$ and $c > 0$ are

constants independent of k.

It follows that $\mathbf{C}^{(k)} = (C \otimes C)^k = C^k \otimes C^k$ has the form

$$\mathbf{C}^{(k)} = \begin{bmatrix} 1 & \mathbf{b}^{(k)} \\ \underline{0} & \mathbf{A}^{(k)} \end{bmatrix}$$

where $\mathbf{b}^{(k)}$ is $1 \times (n^2 - 1)$ and $\mathbf{A}^{(k)}$ is $(n^2 - 1) \times (n^2 - 1)$. The eigenvalues of $C^k \otimes C^k$ are all of the form $\lambda\mu$ where λ and μ are eigenvalues of C^k. Thus 1 is a simple eigenvalue and all other eigenvalues λ have $|\lambda| < c\nu^k$ where $0 < \nu < 1$ and c are constants independent of k. Consequently, all eigenvalues of $\mathbf{A}^{(k)}$ have the latter property.

Moreover, if $b^{(k)} = (b_1^{(k)}, \ldots, b_{n-1}^{(k)})$ then the top row of $\mathbf{C}^{(k)}$ is obtained by concatenating the row vectors $1 \otimes [1\ b^{(k)}]$ and $b^{(k)} \otimes [1\ b^{(k)}]$ for $i = 1, \ldots, n-1$, and therefore the entries in this row converge exponentially fast to finite limits. Consequently, if we write $\mathrm{vec}(V)$ as $\alpha_1 E_1 + Z$ where $E_1 = (1, 0, \ldots, 0)^T$ and $Z = (0, \underline{z})^T$ is a vector perpendicular to E_1,

$$\mathbf{C}^{(k)}\mathrm{vec}(V) = \begin{bmatrix} \alpha_1 + \mathbf{b}^{(k)}\underline{z} \\ \mathbf{A}^{(k)}\underline{z} \end{bmatrix}$$

and the top entry converges exponentially fast to a limit $\alpha_1 + \mathbf{b}^{(\infty)}\underline{z} > 0$ i.e. $|\mathbf{b}^{(k)} - \mathbf{b}^{(\infty)}| \leq c\nu^k$ where $0 < \nu < 1$ and c are constants independent of k.

It follows immediately by (S8.2) that if we write $V(t - N\tau, t)$ in terms of the above coordinates $(x_1, \mathbf{x}_2) = (x_1, x_1, \ldots, x_n)$ as

$$V(t - N\tau, t) = \begin{bmatrix} V_{11} & V_{21}^T \\ V_{21} & V_{22} \end{bmatrix}$$

then, as $N \to \infty$, while V_{21} and V_{22} have finite limits, $V_{11} \to +\infty$. Using the Schur complement, the precision matrix of $P^{(N)} = P_{\text{LNA}}(\xi(0)|X(t - N\tau) = g(t))$ is

$$V(t - N\tau, t)^{-1} = \begin{bmatrix} (V_{11} - V_{21}^T V_{22}^{-1} V_{21})^{-1} & -V_{11}^{-1} V_{21}^T (V_{22} - V_{21} V_{11}^{-1} V_{21}^T)^{-1} \\ -(V_{22} - V_{21} V_{11}^{-1} V_{21}^T)^{-1} V_{21} V_{11}^{-1} & (V_{22} - V_{21} V_{11}^{-1} V_{21}^T)^{-1} \end{bmatrix}.$$

This has a well-defined limit as $N \to \infty$ where, since $V_{11} \to \infty$, all entries but $(V(t-N\tau, t)^{-1})_{22}$ converge to zero and $(V(t - N\tau, t)^{-1})_{22}$ has a finite non-zero limit equal to $(\lim_{n\to\infty} V_{22})^{-1}$. Thus, if $\check{P}^{(N)}$ is the distribution given by conditioning $P^{(N)}$ on $x_1 = 0$ then the precision matrix of $\check{P}^{(N)}$ is $(V_{22} - V_{21} V_{11}^{-1} V_{21}^T)^{-1}$. Consequently, the covariance matrix of $\check{P}^{(N)}$ is $V_{22} - V_{21} V_{11}^{-1} V_{21}^T$

which in the limit $N \to \infty$ equals $V_{22}^{(\infty)} = \lim_{n\to\infty} V_{22}$. We thus deduce that the conditional distribution converges as $n \to \infty$ to a MVN distribution P with this covariance matrix. Since P is derived via the limiting process as $N \to \infty$, it is clear that $P_{\text{LNA}}(Q_{x_i}^{(2)}|Q_{x_i}^{(1)} \sim P) = P$ so $P = P_{\mathcal{S}}^{(\text{fp})}$.

8.2 Extending this result to arbitrary transversals.

Any transversal hyperplane $\mathcal{S}$ is of the form $W\mathcal{S}_N$ where $\mathcal{S}_N$ is the normal hyperplane and W is a $n \times n$ orthogonal matrix with $W_{11} > 0$. This latter condition ensures that $\mathcal{S}$ is transversal to the circle. Thus if $\mathbf{z} = (z_1, \mathbf{z}_2) = W\mathbf{y}$ then in the $\mathbf{z}$ coordinate system $\mathcal{S}$ is given by $z_1 = 0$. In this $\mathbf{z}$ coordinate system, the precision matrix of $P = P_{\mathcal{S}}^{(\text{fp})}$ is given by $WV(t - n\tau, t)^{-1}W^T$. However, from (8.1)

$$WV(t-n\tau,t)^{-1}W^T \to \begin{bmatrix} W_{12}(V_{22}^{(\infty)})^{-1}W_{12}^T & W_{12}(V_{22}^{(\infty)})^{-1}W_{22}^T \\ \\ W_{22}(V_{22}^{(\infty)})^{-1}W_{12}^T & W_{22}(V_{22}^{(\infty)})^{-1}W_{22}^T \end{bmatrix}.$$

Therefore, in the limit, $\mathbf{z}_2|(z_1 = 0)$ has covariance matrix $W_{22}V_{22}^{(\infty)}W_{22}^T$.

8.3 Alternative geometrical proof that convergence on $\mathcal{S}_N$ implies convergence on other transversals $\mathcal{S}$.

Let $\mathcal{S}_N$ be given by $y_1 = 0$ in the $\mathbf{y}$ coordinate system. Consider the ellipsoid $\mathcal{E}$ given by $\mathbf{y}V_n^{-1}\mathbf{y} = \mathbf{y}_0V_n^{-1}\mathbf{y}_0$ where $\mathbf{y}_0 \in \mathcal{S}_N$. If $p \in \mathcal{S}_N$ consider $p + \delta e_1$ where $\delta \in \mathbb{R}$ and e_1 is the vector $(1, 0, \ldots, 0)$ in the $\mathbf{y}$ system. Clearly there is a unique δ such that $p + \delta e_1 \in \mathcal{S}$. Denote this point by $\phi(p)$. Then ϕ is linear. Consider $Q_n(p) = \phi^{-1}(p)^T V_n^{-1}\phi^{-1}(p)$. By the above result for $\mathcal{S}_N$, on $\mathcal{S}_N$, Q_n converges to a $Q_{\infty,N}$. In the region $|y_1| < r$, the ellipsoids given by $Q_n(p) = \varepsilon > 0$ converge to cylinders of the form $\{(y_1, \mathbf{y}_2) : Q_{\infty,N}(\mathbf{y}_2) = \varepsilon\}$. Therefore, $Q_n(p) \to Q_\infty(p)$ where $Q_\infty(p) = Q_{\infty,N}(\mathbf{y}_2)$ if p has coordinate $(y_1, \mathbf{y}_2)$.

Extension to $P(Q_{x_j}^{(N)}|Q_{x_i}^{(1)})$, $x_j = g(t_j), x_i = g(t_i)$, $t_j \geq t_i$. The result is easily derived using that $V(t_i, t_j + N\tau) = C(t_i, t_j)V(t_i, t_i + N\tau)C(t_i, t_j)^T + V(t_i, t_j)$ which implies existence of the limit of $V^{-1}(t_i, t_j + N\tau)$, as $N \to \infty$, if $\lim_{N\to\infty} V^{-1}(t_i, t_i + N\tau)$ exists.

8.4 Entrained forced oscillators.

The above proof of Section S8.1 has to be slightly modified as follows. In this case all eigenvalues of $C(s,t)$ have $|\lambda| < 1$ and therefore all eigenvalues λ of $C^k \otimes C^k$ have $|\lambda| < c\nu^k$ where $0 < \nu < 1$

and c are constants independent of k. Consequently, using (S8.2), all entries of $V(t - N\tau, t)$ converge to a finite limit as $N \to \infty$.

This shows that in contradistinction to free-running oscillators, for entrained forced oscillators, $P_{\mathrm{LNA}}(X(N\tau)|X(0))$ converges as $N \to \infty$. However, it does not mean that this is a good approximation to $P(X(N\tau)|X(0))$ for an exact simulation. As we show in the simulation study displayed in S2 Fig. 9, these two distributions appear to remain relatively close to each other as N increases. In these simulations, $P(X(N\tau)|X(0))$ tends to wrap itself around the limit cycle while $P_{\mathrm{LNA}}(X(N\tau)|X(0))$ remains a bounded MVN. On the other hand, the conditional distributions $P_{\mathrm{LNA}}(Q_{x_i}^{(N)}|Q_{x_i}^{(1)})$ do appear to remain accurate as $N \to \infty$.

9 Analytic expression for pcLNA transversal distributions

To calculate an analytic expression for

$$P_{\mathrm{LNA}}(\underline{Q}|X(t_0)) = P_{\mathrm{LNA}}(Q_{x_0}^{(1)}, \dots, Q_{x_q}^{(m)}|X(t_0)) \tag{S9.1}$$

we need to introduce some new matrices. At each of the points x_k we fix a coordinate system $\mathcal{C}_{x_k}$ defined by a set of orthonormal vectors $e_1(x_k), \dots, e_n(x_k)$ where $e_1(x_k)$ is normal to the hyperplane $\mathcal{S}_{x_k}$. We write coordinates in $\mathcal{C}_{x_k}$ in the form $(y_1, \boldsymbol{y}_2)$ where $y_1 \in \mathbb{R}$ and $\boldsymbol{y}_2 \in \mathbb{R}^{n-1}$ and $\boldsymbol{y}_2$ are coordinates on $\mathcal{S}_{x_k}$. The matrices $C(s,t)$ and $V(s,t)$ in this coordinate systems are

$$C(s,t) = \begin{bmatrix} C_{11} & C_{12} \\ \\ C_{21} & C_{22} \end{bmatrix} \quad \text{and} \quad V(s,t) = \begin{bmatrix} V_{11} & V_{12} \\ \\ V_{21} & V_{22} \end{bmatrix},$$

where $V_{12} = V_{21}^T$. Let $\check{C}(s,t) = C_{2\cdot} - V_{21}V_{11}^{-1}C_{1\cdot}$ and $\check{V}(s,t) = V_{22} - V_{21}V_{11}^{-1}V_{12}$. Note that $\check{V}(s,t)$ is the Schur complement of V_{22} in $V(s,t)$ so if $(y_1, \boldsymbol{y}_2)$ is MVN with covariance $V(s,t)$ then the distribution of $\boldsymbol{y}_2$ conditional on y_1 having a specific value has covariance $\check{V}(s,t)$.

Firstly, we consider $P_{\mathrm{LNA}}(Q_{x_j}^{(l)}|Q_{x_i}^{(k)})$ where either $k < l$ or $k = l$ and $i < j$. In Section S7 we showed that this is very well approximated by a MVN distribution with mean $\check{C}_j Q_{x_i}^{(k)}$ and covariance $\check{V}_j$ where

$$\check{C}_j = \check{C}(t_j, t_j - t_i + (l-k)\tau), \; \check{V}_j = \check{V}(t_j, t_j - t_i + (l-k)\tau).$$

We also proved in Section 8 that as $l - k \to \infty$, $P_{\mathrm{LNA}}(Q_{x_j}^{(l)}|Q_{x_i}^{(k)})$ converges to a limit P_{x_j} which is independent of $Q_{x_i}^{(k)}$ and does this exponentially fast in the sense that the mean and covariance

of $P_{\text{LNA}}(Q_{x_j}^{(l)}|Q_{x_i}^{(k)})$ converge exponentially fast to their limiting values.

9.1 Fixed point distribution.

The special case where the transitions are returns to the same transversal sections ($i = j$) is especially interesting. Clearly, $P_{\text{LNA}}(Q_{x_i}^{(k)}|Q_{x_i}^{(1)})$ converges to the same limit P_{x_i} as above and this distribution is a fixed point in the sense that the distribution $P_{\text{LNA}}(Q_{x_i}^{(k+1)}|Q_{x_i}^{(k)} \sim P_{x_i}) = P_{x_i}$. We therefore denote it $P_{x_i}^{(\text{fp})}$. Because of the aforementioned convergence the transversal distribution has the following important property

$$\lim_{k\to\infty} P_{\text{LNA}}(Q_{x_i}^{(k)}|X(t_0)) = P_{x_i}^{(\text{fp})}$$

for any initial condition $X(t_0)$. That is, the distribution of the system at any transversal section converges to a limiting multivariate normal distribution.

This distribution can be easily calculated numerically. To derive its mean and covariance matrix one solves the equations $m = \check{C}_i m$ and $S = \check{C}_i S \check{C}_i^{\,T} + \check{V}_i$, respectively. The latter can be solved by using the fact that $\text{vec}(\check{C}_i S \check{C}_i^T) = (\check{C}_i \otimes \check{C}_i)\text{vec}(S)$ which implies that

$$\text{vec}(S) = (I - \check{C}_i \otimes \check{C}_i)^{-1}\text{vec}(\check{V}_i). \tag{S9.2}$$

Here vec(S) is the vector obtained by stacking all the columns of S on top of each other and $\otimes$ is the Kronecker or tensor product (see e.g. Chapter 4 of [7]).

For the circadian clock the convergence is fast: the L^2-norm of the difference between the covariance matrices of the limiting distribution, $P_{x_0}^{(\text{fp})}$ and $P_{\text{LNA}}(Q_{x_0}^{(r)}|X(t_0))$ for $r = 1, 2, \ldots, 5$ is respectively $(1250, 70, 3.9, 0.2, 0.1) \cdot 10^{-3}$.

9.2 The joint distribution of multiple transversals

We now consider the distribution of the sequence of transitions between transversal sections of different phases $P_{\text{LNA}}(\underline{Q}|X(t_0))$. We relabel $Q_{x_0}^{(1)}, \ldots, Q_{x_q}^{(1)}, \ldots, Q_{x_0}^{(m)}, \ldots, Q_{x_q}^{(m)}$ as $Q_1, \ldots, Q_N$ where $N = m(q+1)$. To each $Q_{x_i}^{(k)}$ there is a corresponding time $t_i + (k-1)\tau$. We label these times in increasing order as $T_1, \ldots T_N$ so that T_n corresponds to Q_n. Let $T_0 = t_0$. With this notation if follows from the above that $P_{\text{LNA}}(Q_{i+1}|Q_i)$ is approximately MVN with mean $\check{C}_i Q_i$ and covariance $\check{V}_i$ where $\check{C}_i = \check{C}(T_i, T_{i+1})$ and $\check{V}_i = \check{V}(T_i, T_{i+1})$. Consequently, if Q_i has covariance matrix $\check{S}_i$ then Q_{i+1} has covariance matrix given by $S_{i+1} = \check{V}_i + \check{C}_i S_i \check{C}_i^T$. As

explained in the next section, it follows that $P_{\mathrm{LNA}}(\underline{Q}|Q_0)$ is MVN with mean

$$\mu = (\check{C}_0 Q_0, \ldots, \check{C}_{N-1} \ldots \check{C}_0 Q_0)$$

and covariance Σ where the precision matrix Σ^{-1} is a tridiagonal matrix with only non-zero entries, the main diagonal $\Sigma_{i,i} = \check{C}_i^T \check{V}_i^{-1} \check{C}_i + \check{V}_{i-1}^{-1}$ for $i = 1, 2, \ldots, N-1$ and $\Sigma_{N,N} = \check{V}_{N-1}^{-1}$, upper diagonal $-\check{C}_i^T \check{V}_i^{-1}$ and lower diagonal $-\check{V}_i^{-1} \check{C}_i$, $i = 1, \ldots, N-1$.

9.3 Precision matrix for $P(\underline{Q}|X(t_0))$.

It is clear from the above that the mean of $P_{\mathrm{LNA}}(Q_{m+1}|Q_m)$ is $\check{C}_m Q_m$ and that $\check{S}_{i+1} = \check{V}_i + \check{C}_i S_i \check{C}_i^T$ which imply

$$\begin{aligned}(Q_{m+1} - &\check{C}_m Q_m)^T V_{m+1}^{-1} (Q_{m+1} - \check{C}_m Q_m) \\ &= \begin{bmatrix} Q_m^T & Q_{m+1}^T \end{bmatrix} \begin{bmatrix} \check{C}_m^T V_{m+1}^{-1} \check{C}_m & -\check{C}_m^T V_{m+1}^{-1} \\ & \\ -V_{m+1}^{-1} \check{C}_m & V_{m+1}^{-1} \end{bmatrix} \begin{bmatrix} Q_m \\ \\ Q_{m+1} \end{bmatrix}\end{aligned}$$

and the formula for the precision matrix for $P(\underline{Q}|X(t_0))$ follows from this using $P_{\mathrm{LNA}}(\underline{Q}|X(t_0)) = P_{\mathrm{LNA}}(Q_1|X(t_0)) \prod_{m=1}^{N-1} P_{\mathrm{LNA}}(Q_{m+1}|Q_m)$.

10 Other transversal sections

For each state variable x_i two interesting transversals to γ are given by the submanifolds on which $\dot{x}_i(t) = 0$, i.e. where the maximum or minimum of $x_i(t)$ occurs at time t. These are both given by $F_i(x) = 0$ and their tangent spaces consist of those vectors v such that $\partial F_i / \partial x(g(t)) \cdot v = 0$. A continuous time trajectory intersects these transversal sections at times when the ith variable is at its maxima and minima. Thus $\underline{Q}$ gives the state of the system at their (large volume limit) maxima and minima.

Another very interesting transversal system is that given by the isochrons of γ. These are defined as follows. Let $\Phi(t, x)$ be the flow associated to F. For $x \in \gamma$ the stable manifold $W^s(x)$ of x is the set of points y such that $\Phi(t, y) - \Phi(t, x) \to 0$ as $t \to \infty$. If γ is an elementary and stable limit cycle as defined in Sect. S1, then, in a neighbourhood of γ, these stable manifolds are codimension one submanifolds that fill the neighbourhood. This follows from the Stable Manifold Theorem [6] and is explained in [4]. They are called isochrons because two points in the same isochron asymptotically have the same phase. They are invariant manifolds in the sense that $W^s(\Phi(t, x)) = \Phi(W^s(x), t)$ i.e. at time t the flow caries the stable manifold of x

exactly onto the stable manifold at $\Phi(t,x)$. In particular, if τ is the period then $\Phi(\tau,\cdot)$ maps $W^s(x)$ to $W^s(x)$ and therefore, using the notation above, if $x=g(t)$, the linear map given by $C(t,t+\tau)$ maps the $(n-1)$-dimensional tangent space V_x of $W^s(x)$ to itself. By using the fact that V_x is transverse to γ, or directly from the Invariant Manifold Theorem, it follows that V_x is spanned by the generalised eigenspace of $C(t,t+\tau)$ corresponding to the eigenvalues λ with $|\lambda|<1$. Consequently, a very interesting transversal system is that where $\mathcal{S}_{g(t)}=V_{g(t)}$ is the tangent space to the isochron through $g(t)$. By the invariance we have $C_{12}=0$ so that $\check{C}=C_{22}$.

A final interesting transversal system is based on the V matrices. For an arbitrary point on the limit cycle $x_t=g(t)$, the matrix $V=V(t,t+\tau)$ is symmetric positive definite and therefore its orthonormal eigenvectors $\tilde{e}_i$, $i=1,\dots,n$ span $\mathbb{R}^N$. The eigenvector corresponding to the largest eigenvalue of V is approximately equal to the unit tangent. We can define the transversal section as the hyperplane spanned by $\tilde{e}_2,\dots,\tilde{e}_n$. The advantage of this transversal system is that the largest variability direction is eliminated in the transversal distributions.

11 Details behind the sensitivity analysis.

If P is MVN with mean and covariance $\mu=\mu(\theta)$ and $\Sigma=\Sigma(\theta)$ then the entries of the FIM are given by

$$I_{ij}=\frac{\partial\mu}{\partial\theta_i}^T\Sigma^{-1}\frac{\partial\mu}{\partial\theta_j}+\frac{1}{2}\mathrm{tr}\left(\Sigma^{-1}\frac{\partial\Sigma}{\partial\theta_i}\Sigma^{-1}\frac{\partial\Sigma}{\partial\theta_j}\right). \tag{S11.1}$$

As above we consider a family of probability distributions $P(X,\theta)$ depending on parameters in θ. We assume these are MVN with mean $\mu(\theta)$ and covariance matrix $\Sigma(\theta)$. In calculating the FIM we have to determine the partial derivatives $\partial\mu/\partial\theta_i$ and $\partial\Sigma/\partial\theta_i$. The derivative M of the mapping $\theta\to(\mu(\theta),\Sigma(\theta))$ at a parameter value θ_0 is given by

$$M\cdot\delta\theta=\left(\sum_i\frac{\partial\mu}{\partial\theta_i}\delta\theta_i,\sum_i\frac{\partial\Sigma}{\partial\theta_i}\delta\theta_i\right)$$

where the derivatives are calculated at θ_0.

As is well-known in Information Geometry the set of multivariate normal distributions MVN^n on $\mathbb{R}^n$ can be given the structure of a Riemannian manifold in which the Riemannian metric is given the line element $ds^2=d\mu^T\Sigma^{-1}\,d\mu+(1/2)\mathrm{tr}\{(\Sigma^{-1}d\Sigma)^2\}$. Points in this are denoted by $\Theta=(\mu,\Sigma)$ where μ is the mean and Σ the covariance matrix. The inner product

in the tangent space at $\Theta_0 = (\mu(\theta_0), \Sigma(\theta_0))$ is given by

$$\langle \delta\Theta_1, \delta\Theta_2 \rangle_{\Theta_0} = \delta\mu_1 \Sigma^{-1} \delta\mu_2 + \frac{1}{2} \mathrm{tr}\left(\Sigma^{-1}\delta\Sigma_1\Sigma^{-1}\delta\Sigma_2\right) \tag{S11.2}$$

where $\Theta_0 = (\mu, \Sigma)$ and $\delta\Theta_j = (\delta\mu_j, \delta\Sigma_j)$, $j = 1, 2$.

The derivative M above at θ_0 is a linear map of the parameter space into MVN^n and therefore (taking the standard inner product $\langle \delta\theta_1, \delta\theta_2 \rangle = \delta\theta_1^T \cdot \delta\theta_2$ on the parameter space) has an adjoint M^* such that $\langle M\delta\theta_1, M\delta\theta_2 \rangle_{\mathrm{IG}} = \delta\theta_1^T M^* M \delta\theta_2$. In fact, the adjoint operator M^* to M is given by

$$M^* U = (\eta_1, \ldots, \eta_s)$$

where

$$\eta_j = \left\langle \frac{\partial\Theta}{\partial\theta_j}, U \right\rangle_{\Theta_0}.$$

By (S11.1) and (S11.2), $\delta\theta_1^T M^* M \delta\theta_2 = \langle M\delta\theta_1, M\delta\theta_2 \rangle_{\Theta_0} = \delta\theta_1^T F \delta\theta_2$. and therefore it follows that

$$M^* M = F. \tag{S11.3}$$

By applying Singular Value Decomposition appropriately (see below) we can find orthonormal vectors V_i spanning the parameter space $\mathbb{R}^s$, s orthonormal vectors U_i in the space MVN^n and numbers $\sigma_1 \geq \cdots \geq \sigma_s$ such that

$$MV_i = \sigma_i U_i, \qquad i = 1, \ldots, s. \tag{S11.4}$$

Note that the orthonormality of the U_i is with respect to the inner product $\langle \cdot, \cdot \rangle_{\Theta_0}$ It follows from (S11.4) that $M^* M V_i \cdot V_j = \sigma_i \sigma_j \delta_{ij}$ and therefore that $FV_i = M^* M V_i = \sigma_i^2 V_i$.

We regard a $s \times s$ matrix S'_{ij} as a sensitivity matrix if there is a corresponding basis for MVN^n $U'_i = (U_i^{\mu\prime}, U_i^{\Sigma\prime})$ such that for any change of parameters $\delta\theta$, the change in μ and Σ is given by

$$\delta\mu = \sum_i U_i^{\mu\prime} \left(\sum_j S'_{ij} \delta\theta_j \right) + \mathrm{O}(||\delta\theta||^2)$$

$$\delta\Sigma = \sum_i U_i^{\Sigma\prime} \left(\sum_j S'_{ij} \delta\theta_j \right) + \mathrm{O}(||\delta\theta||^2) \tag{S11.5}$$

Note that the role of the S'_{ij} as sensitivities is seen from the following relation which follows

from (S11.5),

$$\|\delta\Theta\| = \|S \cdot \delta\theta\| + O(\|\delta\theta\|^2). \tag{S11.6}$$

With this definition $S_{ij} = \sigma_i V_{ji}$ is a sensitivity matrix (with $U_i' = U_i$) which has the following optimality property: for all $k = 1, \dots, s$

$$\sum_{i \leq k} \sum_j S_{ij}^2 \geq \sum_{i \leq k} \sum_j S_{ij}'^2 \quad \text{and} \quad \sum_{i > k} \sum_j S_{ij}^2 \leq \sum_{i > k} \sum_j S_{ij}'^2. \tag{S11.7}$$

This tells us that the basis U_i and the corresponding sensitivities S_{ij} are optimal for capturing as much sensitivity as possible in the low order principal components. Note also that (i) the rows $S_i^{(r)}$ of S_{ij} are orthogonal, and (ii) $S_i^{(r)} \cdot V_j = \sigma_i \delta_{ij}$.

11.1 Deducing equation (S11.4) from SVD.

We can write the points of MVN^n in Cartesian coordinates as $(\mu, \mathrm{vec}(\Sigma))$ and a straightforward calculation shows that in this representation the inner product $\langle (\mu_1, \Sigma_1), (\mu_2, \Sigma_2) \rangle_{(\mu,\Sigma)}$ is given by

$$\mu_1^T \Sigma^{-1} \mu_2 + \frac{1}{2} vec(\Sigma_1)(I \otimes \Sigma^{-1})^T (I \otimes \Sigma^{-1}) vec(\Sigma_2)$$

which can be written as $(\mu_1, vec(\Sigma_1))\mathcal{F}(\mu_1, vec(\Sigma_2))$ where $\mathcal{F}$ the $(n + n^2) \times (n + n^2)$ square matrix

$$\mathcal{F} = \begin{bmatrix} \Sigma^{-1} & \mathbf{0} \\ \mathbf{0} & (I \otimes \Sigma^{-1})^T (I \otimes \Sigma^{-1}) \end{bmatrix}. \tag{S11.8}$$

Let $R^T R = \mathcal{F}$ be the Cholesky decomposition of $\mathcal{F}$. Then, if $y_i = R \cdot (\mu_i, \mathrm{vec}(\Sigma_i))^T$, $i = 1, 2$, the above inner product equals $y_1^T \cdot y_2$. Thus, we let WDV^T be the classical SVD of RM where D is a diagonal matrix with entries $\sigma_1 \geq \cdots \geq \sigma_s$. Then the columns V_i of V and the vectors $U_i = R^{-1} W_i$ (W_i the ith column of W) satisfy (S11.4). This also gives a practical algorithm to calculate the V_i, U_i and σ_i.

12 Multiple crossings in each rotation

The exact stochastic trajectories considered in this article are generated by the SSA that simulates from the Markov jump process with rates dependent on the state of the system as described in **I**. In this Markov jump process, at almost every state of the system, $X(t)$, with phase $G(X(t)) = g(s)$, $s > 0$, there is a possibility for the next point, $X(t+\delta)$, where δ the time of the next jump, to have phase $G(X(t + \delta)) = g(s')$ with $s' < s$. That is, the probability of backward (in terms of phase) moves is positive in almost every state, with only few exceptions

(e.g. absorbing states).

If we fix a phase and consider the crossings of the exact stochastic trajectory to the corresponding transversal section, the probability of more than one crossings in each rotation is again almost always positive. There are a few related measures that are worth considering here. First, the number of crossings in each rotation that we know it is finite as the Markov jump process can only perform a finite number of jumps in a finite time interval. An interesting question is how fast the probability of the number of crossings declines with increasing number of crossings. Secondly, how much time does the stochastic trajectory spend in this forward and backward moves and how does this compare with the period of the limit cycle. Thirdly, whether the distribution of the first crossings differ from the distribution of the second crossings and henceforth.

To address these questions we performed a simulation study using the *Drosophila* circadian clock system for system size $\Omega = 300$. We computed 1000 simulated trajectories by letting the SSA run for a time interval of 4.5 periods of the limit cycle solution. Note that this simulation study requires all jumps of the SSA to be recorded and therefore it requires a substantially greater computer memory space than the other simulation studies where each trajectory is thinned.

As we can see in Figure A, the probability of the number of crossings declines exponentially fast, with only $\approx 20\%$ of the trajectories giving more than 5 crossings. Furthermore, as we can see in Figure B, the distribution of the time between the first and the last crossing is also declining exponentially fast with only $\approx 5\%$ time intervals being larger than 0.014 which is $O(10^{-4})$ compared to the period of the limit cycle. Finally, as we can see in Figure C, the distribution of the points at the different crossings of the same transversal section does not differ substantially in any rotation or transversal coordinate. The deviations are expected to decline with smaller variability levels (larger Ω) as well as the number of crossings and the time spend at each phase.

This suggests that the transversal points derived using the thinned stochastic trajectories of the SSA are good representation of all crossings, at least for the variability levels considered in this article.

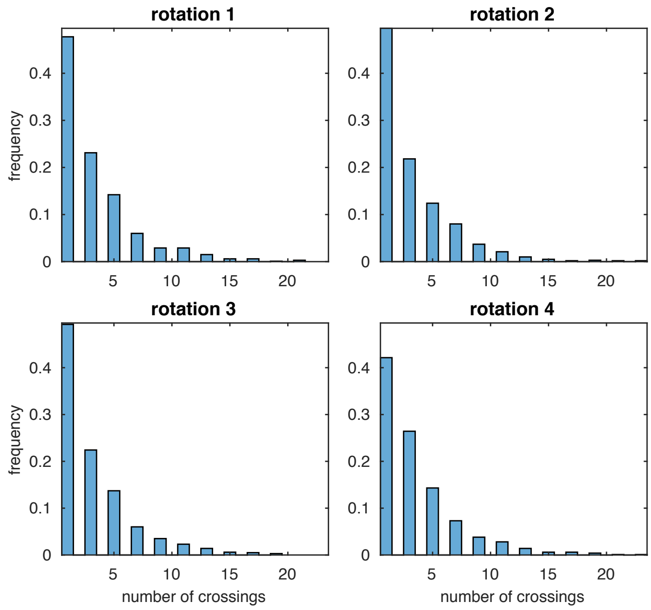

Figure A: Histograms of the empirical distribution of the number of crossings from a transversal section in each of the four rotations of the stochastic trajectories of the *Drosophila* circadian clock simulated using the SSA at system size $\Omega = 300$.

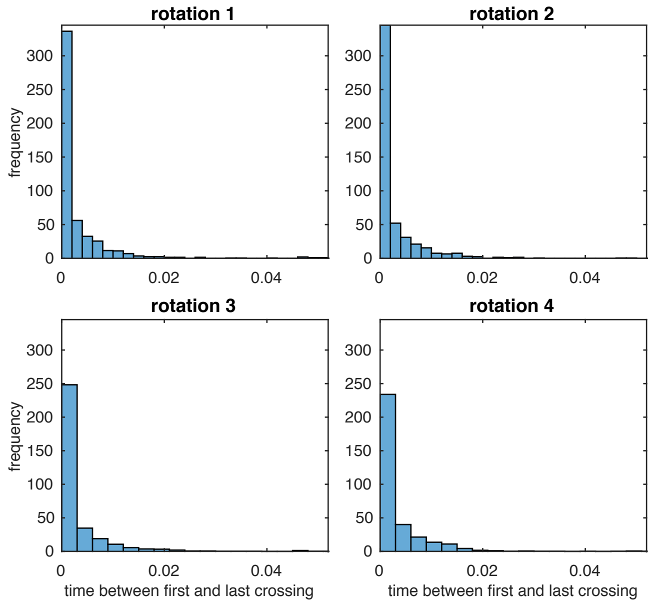

Figure B: Histograms of the empirical distribution of the time between the first and the last crossing from a transversal section in each of the four rotations of the stochastic trajectories of the *Drosophila* circadian clock simulated using the SSA at system size $\Omega = 300$.

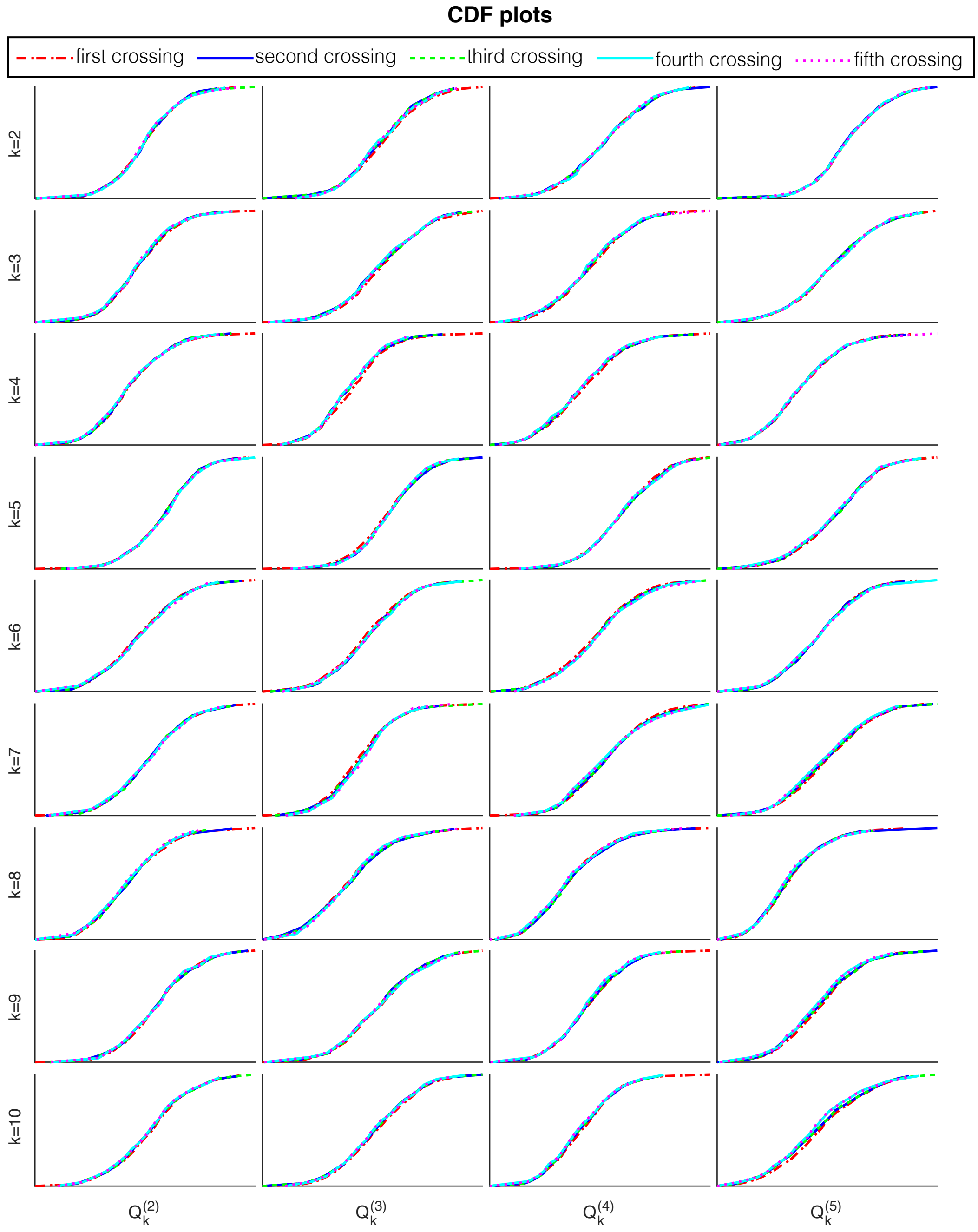

Figure C: Empirical CDF plots of the distribution of the points, $Q_k^{(r)}$, on the transversal section at the first, second, ..., fifth crossings (see legend) in each of the four rotations, $r = 1, 2, 3, 4$ and for transversal coordinates $k = 2, 3, \ldots, 10$ of the stochastic trajectories of the *Drosophila* circadian clock simulated using the SSA at system size $\Omega = 300$.

It is also worth clarifying here that for the pcLNA transversal distributions, as we discuss in Section S6 the distributions of the points in all crossings is considered and then approximated by the distribution of the crossing point at a specific time.

13 Negative populations

For the tau-leap, integration of CLE and pcLNA approximation algorithms considered here, the probability of generating negative populations is non-zero. To make the comparison fair to the SSA exact algorithm, we slightly modify these approximation algorithms to ensure that no negative species populations are derived. We use the same simple approach for all approximation algorithms to ensure the comparison between the approximations is fair. This is simply to replace any steps that result in negative populations by an SSA simulation run for the same time interval and then continue with the approximation algorithm. This increases CPU times as SSA is slower than the simulation algorithms, but also improves precision by introducing some bias towards SSA results. However, from our experience, the effect of this change in our comparisons is minimal in terms of precision and substantially affects CPU times only in rare cases. In particular, for large Ω $(= 1000, 3000)$ the SSA simulation is very rarely employed. For pcLNA, we also take advantage of the property, which also applies to standard LNA, of being able to analytically compute the distribution at the next time-point which is MVN and, before using the SSA step to replace negative populations, we sample from a truncated MVN distribution. For the latter we use a Matlab program[1] that is much faster than SSA simulation but in the challenging circumstances of the illustrations used in this article, it often fails to generate samples. In that case, an SSA simulation is used as in the other algorithms.

14 PeTTSy

PeTTSy is freely available at `http://www2.warwick.ac.uk/fac/sci/systemsbiology/research/software/`.

In PeTTSy the drift and diffusion matrices, $C(t)$ and $V(t)$, respectively, are computed for a large set of time-points. We next provide the description of the computation method for the drift matrix $C(s,t)$.

The deterministic solution is divided into a number of blocks, $tb_i, tb_{i+1} \ldots, tb_N$. By default, $N = 70$ blocks. The model Jacobian $J(t)$ is integrated over each of these time blocks in both directions to give forward and reverse solutions for each time block. Forward solution, $C(s,t)$ where s is tb_i, t runs from tb_i to tb_{i+1} is a solution of

$$\frac{dC(s,t)}{dt} = J(t) \cdot C(s,t)$$

[1]http://uk.mathworks.com /matlabcentral/fileexchange/34402-trunca ted-multivariate-normal

with initial condition $C(s, s) = I$ and I the identity matrix. Reverse solution, $C(s, t)$, where t is tb_i, s runs from tb_i to tb_{i-1} is a solution of the adjoint equation

$$\frac{dC(s,t)^T}{ds} = -J(s)^T \cdot C(s,t)^T$$

with initial condition $C(t, t) = I$. This is equivalent to solving,

$$\frac{dC(t-u,t)^T}{du} = J(t-u)^T \cdot C(t-u,t)^T \tag{S14.1}$$

for $t = tb_i$, $i = 1, 2, \ldots, N$, and where u runs from 0 to $tb_i - tb_{i-1}$. The results of this is that the forward and backward solutions allow us to construct any solution, $C(a, b)$, where if $a < tb_i$ and $b > tbi$, we can write the following

$$C(a,b) = C(tb_i, b) \cdot C(a, tb_i)$$

where $C(a, tbi)$ comes from the reverse solutions and $C(tb_i, b)$ comes from the forward solution. However, when a and b are inside the same interval, $[tb_i, tb_{i+1}]$ then

$$C(a,b) = C(tb_i, b) \cdot C(a, tb_i)^{-1}$$

or,

$$C(a,b) = C(tb_{i+1}, b)^{-1} C(a, tb_{i+1})$$

namely we have to invert either the reverse solution or the forward solution in order to calculate $C(a, b)$. PeTTSy provides a condition number that is equal to the minimum of one over the eigenvalue of the forward and backward solutions and if it is too large the matrix may be close to singular and then the inverse inaccurate. This means that forward and reverse solutions were not calculated finely enough, i.e., there were too few time blocks, and this will affect the accuracy of the results. One can check the accuracy of the $C(s, t)$ matrices, after forward and reverse solutions have been calculated, by a plot of the condition numbers. As a guide, if condition numbers are above $1/eps$, where eps is the floating accuracy of Matlab, the inverse is not accurate. The user is then advised to increase the value of N.

In a similar fashion, forward and reverse solutions are computed for the diffusion matrix V, using the equation (S4.3). Alternatively, one may solve the ODE

$$\frac{dV}{dt} = JV + VJ^T + EE^T,$$

where $E = E(s)$ the matrix described in equation (S4.3), with initial condition $V(t_0, t_0) = 0$. The reverse solutions are derived in a similar fashion to above by using the ODE

$$\frac{dV(t-u,t)^T}{du} = J(t-u)V(t-u,t) + V(t-u,t)J(t-u)^T + E(t-u)E(t-u)^T, \qquad \text{(S14.2)}$$

with initial condition $V(t_0, t_0) = 0$, for $t = tb_i$, $i = 1, 2, \ldots, N$, and where u runs from 0 to $tb_i - tb_{i-1}$.

15 The pcLNA Kalman Filter

The pcLNA Kalman Filter algorithm uses the following recursive algorithm for computing the terms in $L\left(\theta; \underline{\hat{X}}\right)$. Note that (μ^*, Σ^*) denote posterior estimates of (μ, Σ) conditional on the observed measurement at the current time.

1. Input $\left(\hat{X}(t_0), \hat{X}(t_1), \ldots, \hat{X}(t_N)\right)$, $\mu(t_0)$, $\Sigma(t_0)$, B and Σ_ϵ.
2. Compute $P\left(\hat{X}(t_0); \theta\right)$ from $MVN(\hat{\mu}(t_0), \hat{\Sigma}(t_0))$.
3. **For iteration** $i = 1, 2, \ldots$
   (a) Derive $(X(t_{i-1})|\hat{X}(t_{i-1})) \sim MVN(\mu^*(t_{i-1}), \Sigma^*(t_{i-1}))$;
   (b) Compute $g(s_{i-1}) = G(\mu^*(t_{i-1}))$ and set $(\kappa(s_{i-1})|\hat{X}(t_{i-1})) \sim MVN(m^*(s_{i-1}), S^*(s_{i-1}))$;
   (c) Derive $(X(t_i)|\hat{X}(t_{i-1})) \sim MVN(\mu(t_i), \Sigma(t_i))$;
   (d) Compute $P\left(\hat{X}(t_i)|\hat{X}(t_{i-1}); \theta\right)$ from $MVN(\hat{\mu}(t_i), \hat{\Sigma}(t_i))$.

In the for loop $\mu^*(t_{i-1}) = \mu(t_{i-1}) + \Sigma(t_{i-1})B^T\hat{\Sigma}(t_{i-1})^{-1}\hat{\epsilon}(t_{i-1})$, $\Sigma^*(t_{i-1}) = \Sigma(t_{i-1}) - \Sigma(t_{i-1})B^T\hat{\Sigma}(t_{i-1})^{-1}B\Sigma(t_{i-1})$, $m^*(s_{i-1}) = \Omega^{1/2}(\mu^*(t_{i-1}) - g(s_{i-1}))$, with $\hat{\epsilon}(t_i) = \hat{X}(t_i) - \hat{\mu}(t_i)$ and

$$S^*(s_{i-1}) = E_2(s_{i-1})\left(S^*_{22}(s_{i-1}) - \frac{S^*_{21}(s_{i-1})S^*_{12}(s_{i-1})}{S^*_{11}(s_{i-1})}\right)E_2(s_{i-1})^T$$

where the matrix $E_2(t) = [e_2(t) \ldots e_n(t)]$ has columns orthonormal vectors which are also orthogonal to the unit tangent vector $e_1(t) = F(x(t))/\|F(x(t))\|$ and

$$S^*_{11}(t) = e_1(t)^T S^*(t) e_1(t), \quad S^*_{12}(t) = e_1(t)^T S^*(t) E_2(t),$$

$$S^*_{21}(t) = S^*_{12}(t)^T, \quad S^*_{22}(t) = E_2(t)^T S^*(t) E_2(t).$$

The measurement equation (Eq. 12 in **I**) is used to derive the predictive probabilities $P(\hat{X}(t_0); \theta)$ and $P(\hat{X}(t_i)|\hat{X}(t_{i-1}); \theta)$ in steps 2 and 3(d), respectively. The posterior distribution $(X(t)|\hat{X}(t))$ in step 3(a) is derived by Bayes rule, while the posterior distribution of the corrected noise process $(\kappa(s)|\hat{X}(t))$ in step 3(b) is derived by restricting the poste-

rior distribution of the noise process on the transversal section $\mathcal{S}_{g(s)}$ normal to $e_1(s)$ using the Schur complement as described earlier. The cartesian coordinate system is used here. If this correction is omitted, step 3(b) of the above algorithm is replaced by the standard LNA step, $(\xi(t_{i-1})|\hat{X}(t_{i-1})) \sim MVN(\hat{m}(t_{i-1}), \hat{S}(t_{i-1}))$ where $\hat{m}(t_{i-1}) = \Omega^{1/2}\hat{\mu}(t_{i-1}) - g(t_{i-1})$, $\hat{S}(t_{i-1}) = \Omega\hat{\Sigma}(t_{i-1})$. The LNA Ansantz (Eq. S4.1) and transition (Eq. S4.2) are used to derive the prior distribution $(X(t_i)|\hat{X}(t_{i-1}))$ in step 3(c).

16 Details related to various computations

All computations for the illustrations in **I** and SI including:

1. SSA, tau-leap, integration of diffusion and pcLNA simulation algorithms described in Sections "pcLNA Simulation algorithm" and "Comparisons to other simulation algorithms" (see also the note in S1 Sect. 13),
2. the computation of Fisher Information and principal control coefficients for the sensitivity discussed in the Section "Sensitivity analysis for stochastic systems" and S1 Sect. 11

are done using the PeTTSy software which is discussed in S1 Sect. 14 and it is freely available at `http://www2.warwick.ac.uk/fac/sci/systemsbiology/research/software/`.

The phase correction that involves identifying the normal mapping $G(X) = x \in \gamma$ of a given state $X = X(t)$ of the trajectory is performed by Newton's optimisation minimising the square of the function

$$F(x(s)) \cdot (X - g(x(s))), \quad s > 0.$$

Note that the minimum is achieved when $s > 0$ is such that $X - g(x(s))$ is normal to the tangent vector $F(x(s))$. Multiple repetitions of the above optimisation are performed, using different initial conditions $s > 0$, to ensure that all local minima, $s' = s_1, s_2, \ldots,$ are derived and then, as explained in S1 Sect. 1, $G(X(t)) = x(s)$ with $s = \min_i |s_i - t|$ the closest time to t. Note that other methods of optimisation could be equally applicable.

FIM computation We use the results in [5] to derive the necessary derivatives for the computation of the FIM. For the readers' convenience some of the derivations in chapter V in [5] are reproduced here. Let

$$\dot{x} = F(t, x, \theta), \quad x(t_0) = x_0$$

be the initial value problem with parameter vector θ which has solution $x = g(t, t_0, x_0, \theta)$. The Jacobian matrix $J(t) = J(t, x, \theta)$ at $x = g(t, t_0, x_0, \theta)$ has entries

$$\left(\frac{\partial F}{\partial x}\right)_{k,k'} = \frac{\partial F_k}{\partial x_{k'}}, \quad k, k' = 1, 2, \ldots, n$$

and the Hessian matrix $H(t) = H(t, x, \theta)$ at $x = g(t, t_0, x_0, \theta)$ has entries

$$\left(\frac{\partial^2 F}{\partial x^2}\right) = (H(F_1), \ldots, H(F_n))', \qquad (H(F_k))_{ss'} = \left(\frac{\partial^2 F_k}{\partial x_s \partial x_{s'}}\right).$$

Define

$$w_k = \frac{\partial g}{\partial x_{0,k}}, \qquad v_j = \frac{\partial g}{\partial \theta_j} \quad k = 1, 2, \ldots, n, \; j = 1, 2, \ldots, m.$$

Then, w_k is the solution of

$$\dot{x} = J(t)x, \quad x(t_0) = e_k$$

and e_k a vector with all zero entries except the kth entry which is equal to 1.

Note that the principal fundamental matrix C is the solution of the latter initial value problem. The matrix $C(t_0, t)$ has columns $w_k(t)$, $k = 1, 2, \ldots, n$. Furthermore, v_j is the solution of

$$\dot{x} = J(t)x + h_j(t), \quad x(t_0) = 0, \tag{S16.1}$$

where $h_j(t) = \partial F / \partial \theta_j$.

The $mn \times n$ matrix $(dC/d\theta_1 \ldots dC/d\theta_m)^T$ is the solution of

$$\dot{Z} = \begin{bmatrix} \text{Diag}\left(w_1{}^T\right) H v_1 & \ldots & \text{Diag}\left(w_n{}^T\right) H v_1 \\ \vdots & & \vdots \\ \text{Diag}\left(w_1{}^T\right) H v_m & \ldots & \text{Diag}\left(w_n{}^T\right) H v_m \end{bmatrix} + \text{Diag}(J) Z + \begin{bmatrix} \frac{\partial J}{\partial \theta_1} w_1 & \ldots & \frac{\partial J}{\partial \theta_1} w_n \\ \vdots & & \vdots \\ \frac{\partial J}{\partial \theta_m} w_1 & \ldots & \frac{\partial J}{\partial \theta_m} w_n, \end{bmatrix} \tag{S16.2}$$

where Diag(J) is the $mn \times mn$ block matrix with main diagonal block entries the $n \times n$ matrix J. The $mn \times n$ matrix $(dV/d\theta_1, \ldots, dV/d\theta_m)^T$ has blocks $dV/d\theta_j$, $j = 1, 2, \ldots, m$ which are the solutions of

$$\dot{Z}_j = J Z_j + Z_j J^T + V\left(H^T \text{Diag}\left(v_j\right) + \frac{\partial J^T}{\partial \theta_j}\right) + \left(\text{Diag}\left(v_j{}^T\right) H + \frac{\partial J}{\partial \theta_j}\right) V + B \frac{dR}{d\theta_j} B^T. \tag{S16.3}$$

Here B is the stoichiometry matrix of the system and $dR/d\theta_j$ the diagonal matrix with main

diagonal entries the derivatives of the reaction rates

$$\frac{du_i(x)}{d\theta_j} = \frac{\partial u_i(x)}{\partial x}^T \frac{\partial x}{\partial \theta_j} + \frac{\partial u_i(x)}{\partial \theta_j}, \quad \text{at } x = g(t, t_0, x_0, \theta).$$

In Section "Fisher Information" of **I** we discuss properties of the FIM of the distribution

$$\left(Q_{x_0}, Q_{x_1} | Q_0 = Q_{x_0}^{\infty}\right) \sim N(\mu, \Sigma)$$

where $Q_{x_0}^{\infty}$ the fixed point pcLNA distribution at $x_0 = g(t_0)$. The parameters of this distribution are

$$\mu = (x_0, x_1)^T, \quad \Sigma = S/\Omega,$$

and $S = A^{-1}$ with

$$A = \begin{bmatrix} (\check{C}_{01})^T(\check{V}_{01})^{-1}\check{C}_{01} + (S_0^{\infty})^{-1} & -(\check{C}_{01})^T(\check{V}_{01})^{-1} \\ \\ -(\check{V}_{01})^{-1}\check{C}_{01} & (\check{V}_{01})^{-1} \end{bmatrix}.$$

Here

$$\check{V}_{01} = P_1 V_{01}, \quad \check{C}_{01} = P_1 C_{01}, \quad P_1 = I - \frac{V_{01}\dot{x}_1\dot{x}_1^T}{\dot{x}_1^T V_{01}\dot{x}_1}, \quad C_{01} = C(t_0, t_1),\ V_{01} = V(t_0, t_1) \quad \text{(S16.4)}$$

and S_0^{∞} the solution of the contraction equation

$$\text{vec}(S_0^{\infty}) = (I - \check{C} \otimes \check{C})^{-1}\text{vec}(\check{V})$$

where

$$\check{C} = \check{C}_{10}\check{C}_{01}, \quad \check{V} = \check{C}_{10}\check{V}_{01}\check{C}_{10}^T + \check{V}_{10}.$$

The matrices $\check{V}_{01}$ and $\check{C}_{01}$ are derived in (S16.4) and

$$\check{V}_{10} = P_0 V_{10}, \quad \check{C}_{10} = P_0 C_{10}, \quad P_0 = I - \frac{V_{10}\dot{x}_0\dot{x}_0^T}{\dot{x}_0^T V_{10}\dot{x}_0}, \quad C_{10} = C(t_1, \tau + t_0),\ V_{10} = V(t_1, \tau + t_0)$$

and τ the period of the system. Thus,

$$\frac{d\mu}{d\theta_j} = \left(\frac{dx_0}{d\theta_j}, \frac{dx_1}{d\theta_j}\right)^T,$$

and

$$\frac{d\Sigma}{d\theta_j} = \Omega \frac{dA^{-1}}{d\theta_j} = \Omega A^{-1} \frac{dA}{d\theta_j} A^{-1}, \quad \frac{d\Sigma^{-1}}{d\theta_j} = \Omega \frac{dA}{d\theta_j}.$$

The derivative $dA/d\theta_j$ is a block matrix with blocks

$$\begin{aligned}
\frac{dA_{(1,1)}}{d\theta_j} &= \frac{d\check{C}_{01}}{d\theta_j}^T (\check{V}_{01})^{-1}\check{C}_{01} - \check{C}_{01}^T \check{V}_{01}^{-1} \frac{d\check{V}_{01}}{d\theta_j} \check{V}_{01}^{-1}\check{C}_{01} + \check{C}_{01}^T \check{V}_{01}^{-1} \frac{d\check{C}_{01}}{d\theta_j} - S_0^{\infty -1} \frac{dS_0^{\infty}}{d\theta_j} S_0^{\infty -1}, \\
\frac{dA_{(1,2)}}{d\theta_j} &= \check{C}_{01}^T \check{V}_{01}^{-1} \frac{d\check{V}_{01}}{d\theta_j} \check{V}_{01}^{-1} - \frac{d\check{C}_{01}}{d\theta_j}^T \check{V}_{01}^{-1}, \\
\frac{dA_{(2,1)}}{d\theta_j} &= \check{V}_{01}^{-1} \frac{d\check{V}_{01}}{d\theta_j} \check{V}_{01}^{-1}\check{C}_{01} - \check{V}_{01}^{-1} \frac{d\check{C}_{01}}{d\theta_j}, \\
\frac{dA_{(2,2)}}{d\theta_j} &= -\check{V}_{01}^{-1} \frac{d\check{V}_{01}}{d\theta_j} \check{V}_{01}^{-1}.
\end{aligned}$$

The necessary derivatives for computing the entries

$$I_{(jj')}(\theta) = \Omega \left(\frac{dx_0}{d\theta_j}^T, \frac{dx_1}{d\theta_j}^T \right) A \left(\frac{dx_0^T}{d\theta_j}, \frac{dx_1^T}{d\theta_{j'}} \right)^T + \frac{1}{2} tr \left(\frac{dA}{d\theta_j} A^{-1} \frac{dA}{d\theta_{j'}} A^{-1} \right)$$

of the FIM are derived as described above. In particular, $v_j = dx_i/d\theta_j$ is the solution of (S16.1) and the derivative $d\dot{x}_i/d\theta_j$ used to derive $dP_i/d\theta_j$ is equal to $J(t_i)v_j + g_j(t_i)$. Furthermore, the derivatives $dC/d\theta_j$ and $dV/d\theta_j$ are derived as solutions of the initial value problems in (S16.2) and (S16.3), respectively.

References

[1] Anderson DF & Kurtz TG. Continuous Time Markov Chain Models for Chemical Reaction Networks. In: Koeppl H, Setti G, di Bernardo M, Densmore D, editors. Design and Analysis of Biomolecular Circuits: Engineering Approaches to Systems and Synthetic Biology. New York: Springer.; 2011.

[2] Freidlin, MI, Wentzell, AD, & Szücs, J. . Random Perturbations of Dynamical Systems. Berlin: Springer Berlin; 2014.

[3] Greene JM, Kim JS The calculation of Lyapunov spectra. Physica D: Nonlinear Phenomena. 1987 Aug;24(1-3):213–225.

[4] Guckenheimer J. Isochrons and phaseless sets. Journal of Mathematical Biology. 1975 Sep;1(3):259–273.

[5] Hartman P. Ordinary differential equations. New York: John Wiley & Sons, Inc. London and Sydney: Wiley.; 1964.

[6] Hirsch MW, Pugh CC & Shub M. Invariant manifolds. Springer-Verlag; 1977.

[7] Horn, RA & Johnson CR. Matrix analysis. Cambridge University Press. Cambridge, New York; 1991.

[8] Kurtz TG. Approximation of Population Processes. Regional Conference Series in Applied Mathematics vol. 36. Society for Industrial and Applied Mathematics. 1981.

[9] Van Kampen, NG. Stochastic processes in physics and chemistry. Amsterdam: North Holland; 1997.
