# Supplementary material for: Long-time analytic approximation of large stochastic oscillators: Simulation, analysis and inference
Source: PLoS Comput Biol. 2017 Jul 24;13(7):e1005676. doi: 10.1371/journal.pcbi.1005676 (PMC5555717; doi:10.1371/journal.pcbi.1005676)
Supplement: S2 Appendix — In this note we give details about the Drosophila circadian clock and use this system to illustrate further the accuracy of distributions and simulations discussed in the main paper. (PDF) [file pcbi.1005676.s002.pdf]

Supplementary information S2 for the paper Long-time analytic approximation of large stochastic oscillators: simulation, analysis and inference

Giorgos Minas[1,2] & David A Rand[1,2]

[1]Zeeman Institute for Systems Biology & Infectious Disease Epidemiology Research & [2]Mathematics Institute,

University of Warwick, Coventry CV4 7AL, UK

July 14, 2017

Abstract

We refer to the paper "Long-time analytic approximation of large stochastic oscillators: simulation, analysis and inference" by **I**. In this note we give details about the *Drosophila* circadian clock system and use it to illustrate further the accuracy of distributions and simulations discussed in **I**.

Contents

1 *D*rosophila circadian clock system **1**

2 Exact simulations **5**

3 Exact transversal distributions: normality **6**

4 pcLNA and exact transversal distributions **7**

5 Less frequent correction **9**

6 Comparison between different simulation algorithms **10**

7 Light entrained *D*rosophila Circadian Clock **11**

1 *Drosophila* circadian clock system

A schematic representation of the *Drosophila* circadian clock system, as provided in [1], is displayed in Figure A. The oscillations are driven by the negative feedback exerted on the *per* and *tim* genes by the complex formed from PER and TIM proteins following phosphorylation. The *per* and *tim* mRNA, M_P and M_T, respectively, are transported into the cytosol where they are degraded and translated into protein (P_0 and T_0). These proteins are multiply phosphorylated (PER: $P_0 \to P_1 \to P_2$; TIM: $T_0 \to T_1 \to T_2$) and these modifications can be reversed by a phosphatase. The fully phosphorylated form of the proteins is targeted for degradation and forms a complex, C, which is transported into the nucleus in a reversible manner where the nuclear form of the PER–TIM complex, C_N, represses the transcription of *per* and *tim* genes.

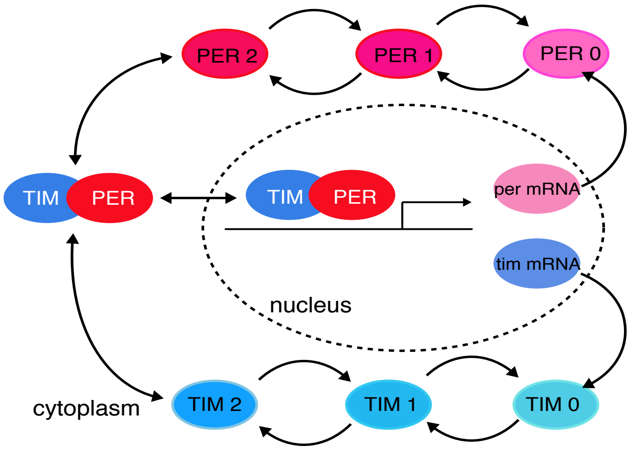

Figure A: Schematic representation of *Drosophila* circadian clock system [1]

The variables of the model along with the initial conditions (in nanomolar concentrations) used in our implementation are provided in Table A.

variable	description	initial condition
M_P	PER mRNA	3.0975
P_0	PER protein 0	1.2547
P_1	PER protein 1	1.2302
P_2	PER protein 2	1.7997
M_T	TIM mRNA	3.0975
T_0	TIM protein 0	1.2346
T_1	TIM protein 1	1.0577
T_2	TIM protein 2	0.3593
C	PER-TIM cytosolic complex	0.6230
C_N	PER-TIM nuclear complex	0.8178

Table A: The variables of *Drosophila* circadian clock system and the initial conditions (in nanomolar concentrations) used to derive their ODE solution.

The parameter values used to derive the ODE solution of the system are provided in Table B.

parameter	description	value	measurement unit
v_{sP}	M_P transcription	1.10	nMh^{-1}
v_{sT}	M_T transcription	1.00	nMh^{-1}
v_{mP}	M_P degradation	1.00	nMh^{-1}
v_{mT}	M_T degradation	0.70	nMh^{-1}
v_{dP}	P_2 degradation	2.20	nMh^{-1}
k_{sP}	M_P translation	0.90	h^{-1}
k_{sT}	M_T translation	0.90	h^{-1}
k_1	$C \rightarrow C_N$	0.80	h^{-1}
k_2	$C_N \rightarrow C$	0.20	h^{-1}
k_3	$P_2 + T_2 \rightarrow C$	1.20	h^{-1}
k_4	$C \rightarrow P_2 + T_2$	0.60	h^{-1}
K_{mP}	M_P enzymatic degradation	0.20	h^{-1}
K_{mT}	M_T enzymatic degradation	0.20	h^{-1}
K_{IP}	M_P Hill coefficient	1.00	h^{-1}
K_{IT}	M_T Hill coefficient	1.00	h^{-1}
K_{dP}	P_2 enzymatic degradation	0.20	h^{-1}
K_{dT}	T_2 enzymatic degradation	0.20	h^{-1}
k_d	linear degradation	0.01	h^{-1}
k_{dC}	C degradation	0.01	h^{-1}
k_{dN}	C_N degradation	0.01	h^{-1}
v_{dT}	T_2 degradation	3.00	nMh^{-1}
K_{1P}	$P_0 \rightarrow P_1$ enzymatic	2.00	h^{-1}
K_{1T}	$T_0 \rightarrow T_1$ enzymatic	2.00	h^{-1}
K_{2P}	$P_1 \rightarrow P_0$ enzymatic	2.00	h^{-1}
K_{2T}	$T_1 \rightarrow T_0$ enzymatic	2.00	h^{-1}
K_{3P}	$P_1 \rightarrow P_2$ enzymatic	2.00	h^{-1}
K_{3T}	$T_1 \rightarrow T_2$ enzymatic	2.00	h^{-1}
K_{4P}	$P_2 \rightarrow P_1$ enzymatic	2.00	h^{-1}
K_{4T}	$T_2 \rightarrow T_1$ enzymatic	2.00	h^{-1}
V_{1P}	$P_0 \rightarrow P_1$	8.00	nMh^{-1}
V_{1T}	$T_0 \rightarrow T_1$	8.00	nMh^{-1}
V_{2P}	$P_1 \rightarrow P_0$	1.00	nMh^{-1}
V_{2T}	$T_1 \rightarrow T_0$	1.00	nMh^{-1}
V_{3P}	$P_1 \rightarrow P_2$	8.00	nMh^{-1}
V_{3T}	$T_1 \rightarrow T_2$	8.00	nMh^{-1}
V_{4P}	$P_2 \rightarrow P_1$	1.00	nMh^{-1}
V_{4T}	$T_2 \rightarrow T_1$	1.00	nMh^{-1}
n	Hill power	4.00	NA

Table B: The parameters of *Drosophila* circadian clock system and the values used to derive their ODE solution.

The ODE system for the *Drosophila* circadian clock is

$$
\begin{aligned}
\dot{M}_P &= v_{sP}\frac{K_{IP}^n}{K_{IP}^n + C_N^n} - v_{mP}\frac{M_P}{K_{mP} + M_P} - k_d M_P \\
\dot{P}_0 &= k_{sP} M_P - V_{1P}\frac{P_0}{K_{1P} + P_0} + V_{2P}\frac{P_1}{K_{2P} + P_1} - k_d P_0 \\
\dot{P}_1 &= V_{1P}\frac{P_0}{K_{1P} + P_0} - V_{2P}\frac{P_1}{K_{2P} + P_1} - V_{3P}\frac{P_1}{K_{3P} + P_1} + V_{4P}\frac{P_2}{K_{4P} + P_2} - k_d P_1 \\
\dot{P}_2 &= V_{3P}\frac{P_1}{K_{3P} + P_1} - V_{4P}\frac{P_2}{K_{4P} + P_2} - k_3 P_2 T_2 + k_4 C - v_{dP}\frac{P_2}{K_{dP} + P_2} - k_d P_2 \\
\dot{M}_T &= v_{sT}\frac{K_{IT}^n}{K_{IT}^n + C_N^n} - v_{mT}\frac{M_T}{K_{mT} + M_T} - k_d M_T \\
\dot{T}_0 &= k_{sT} M_T - V_{1T}\frac{T_0}{K_{1T} + T_0} + V_{2T}\frac{T_1}{K_{2T} + T_1} - k_d T_0 \\
\dot{T}_1 &= V_{1T}\frac{T_0}{K_{1T} + T_0} - V_{2T}\frac{T_1}{K_{2T} + T_1} - V_{3T}\frac{T_1}{K_{3T} + T_1} + V_{4T}\frac{T_2}{K_{4T} + T_2} - k_d T_1 \\
\dot{T}_2 &= V_{3T}\frac{T_1}{K_{3T} + T_1} - V_{4T}\frac{T_2}{K_{4T} + T_2} - k_3 P_2 T_2 + k_4 C - v_{dT}\frac{T_2}{K_{dT} + T_2} - k_d T_2 \\
\dot{C} &= k_3 P_2 T_2 - k_4 C - k_1 C + k_2 C_N - k_{dC} C \\
\dot{C}_N &= k_1 C - k_2 C_N - k_{dN} C_N.
\end{aligned}
$$

The SSA trajectories are derived using the rates provided in Table 1 of [1].

In the simulations of the *Drosophila* circadian clock provided in the next section and in **I**, we consider five values of the system size $\Omega = 200$, 300, 500, 1000 and 3000. In Table C, we provide the approximate ranges of the molecular numbers for each species observed in our simulations. Notice the increase in the maximum number of molecules with increasing Ω, but also the decrease in the length of the range in terms of concentrations, $X(t) = Y(t)/\Omega$, that reflects the lower levels of stochasticity for increasing Ω.

variable	$\Omega = 200$	$\Omega = 300$	$\Omega = 500$	$\Omega = 1000$	$\Omega = 3000$
M_P	$(0, 800)$	$(0, 1200)$	$(0, 1900)$	$(0, 3500)$	$(40, 10000)$
P_0	$(0, 500)$	$(0, 600)$	$(0, 900)$	$(0, 1600)$	$(10, 4500)$
P_1	$(0, 500)$	$(0, 700)$	$(0, 1000)$	$(0, 1700)$	$(10, 4700)$
P_2	$(0, 1400)$	$(0, 1700)$	$(0, 2500)$	$(0, 4300)$	$(30, 10600)$
M_T	$(0, 800)$	$(0, 1200)$	$(0, 1900)$	$(0, 3500)$	$(40, 10000)$
T_0	$(0, 400)$	$(0, 600)$	$(0, 900)$	$(0, 1600)$	$(10, 4000)$
T_1	$(0, 400)$	$(0, 500)$	$(0, 800)$	$(0, 1400)$	$(10, 3900)$
T_2	$(0, 200)$	$(0, 300)$	$(0, 400)$	$(0, 600)$	$(10, 1400)$
C	$(6, 400)$	$(10, 500)$	$(30, 800)$	$(75, 1400)$	$(280, 3700)$
C_N	$(60, 700)$	$(100, 900)$	$(200, 1400)$	$(400, 2600)$	$(1300, 7000)$

Table C: The approximate ranges of the molecular numbers, $Y(t)$, of each of the species of *Drosophila* circadian clock system in simulations derived using SSA for various system sizes Ω.

For more details considering the *Drosophila* circadian clock system see [1].

2 Exact simulations

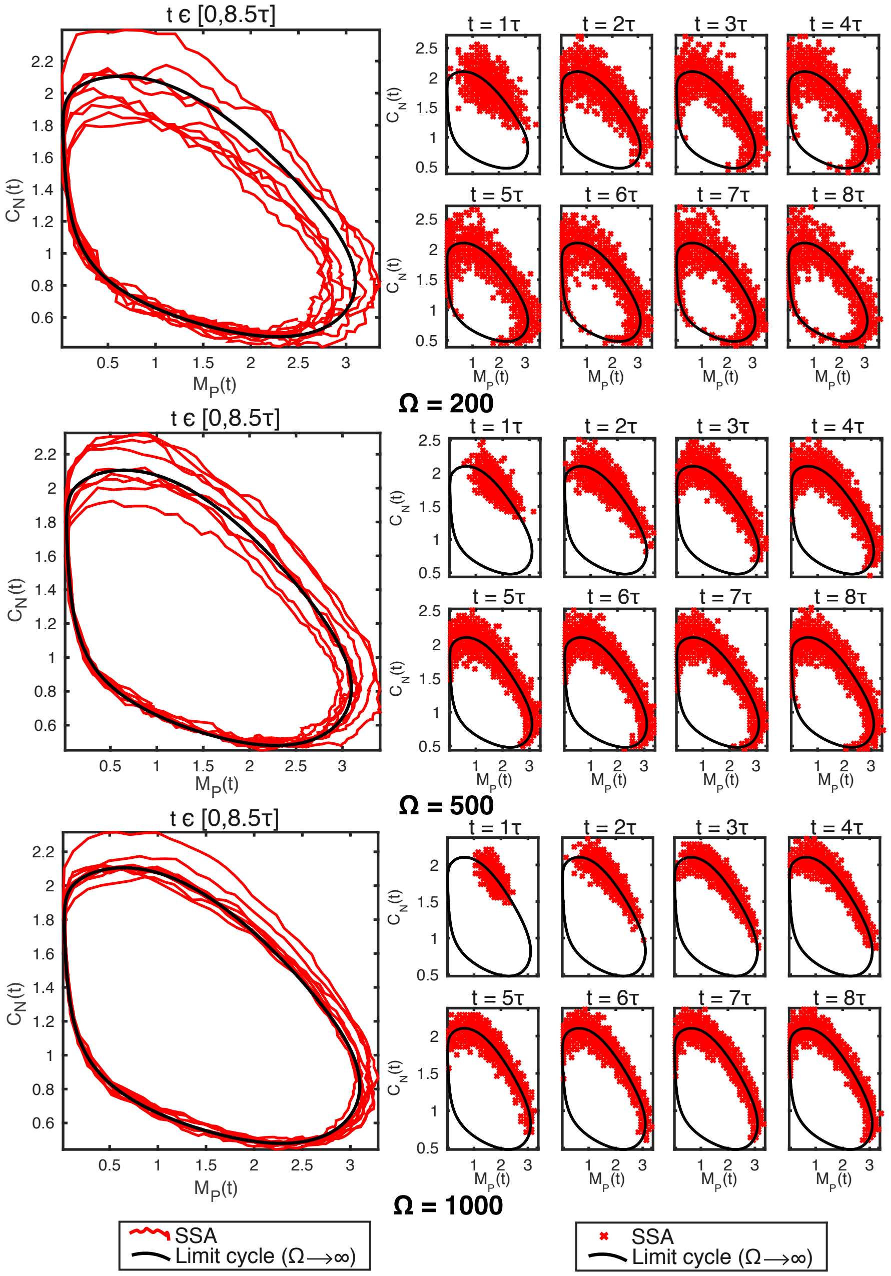

Figure B: Exact stochastic simulation of the *Drosophila* circadian clock system in three different system sizes, $\Omega = 200$ (top panel), $\Omega = 500$ (middle panel), $\Omega = 1000$ (bottom panel). A stochastic trajectory obtained by running the SSA over the time-interval $t \in [0, 8.5\tau]$ is displayed on the left panels and SSA samples ($R = 3000$) at times $t = \tau, 2\tau, \ldots, 8\tau$ are displayed on the right panels. Two (out of 10) of the species are displayed (*per* mRNA M_P (x-axis) and nuclear PER-TIM complex C_N (y-axis)). The black solid curve is the large volume, $\Omega \to \infty$, limit cycle solution.

3 Exact transversal distributions: normality

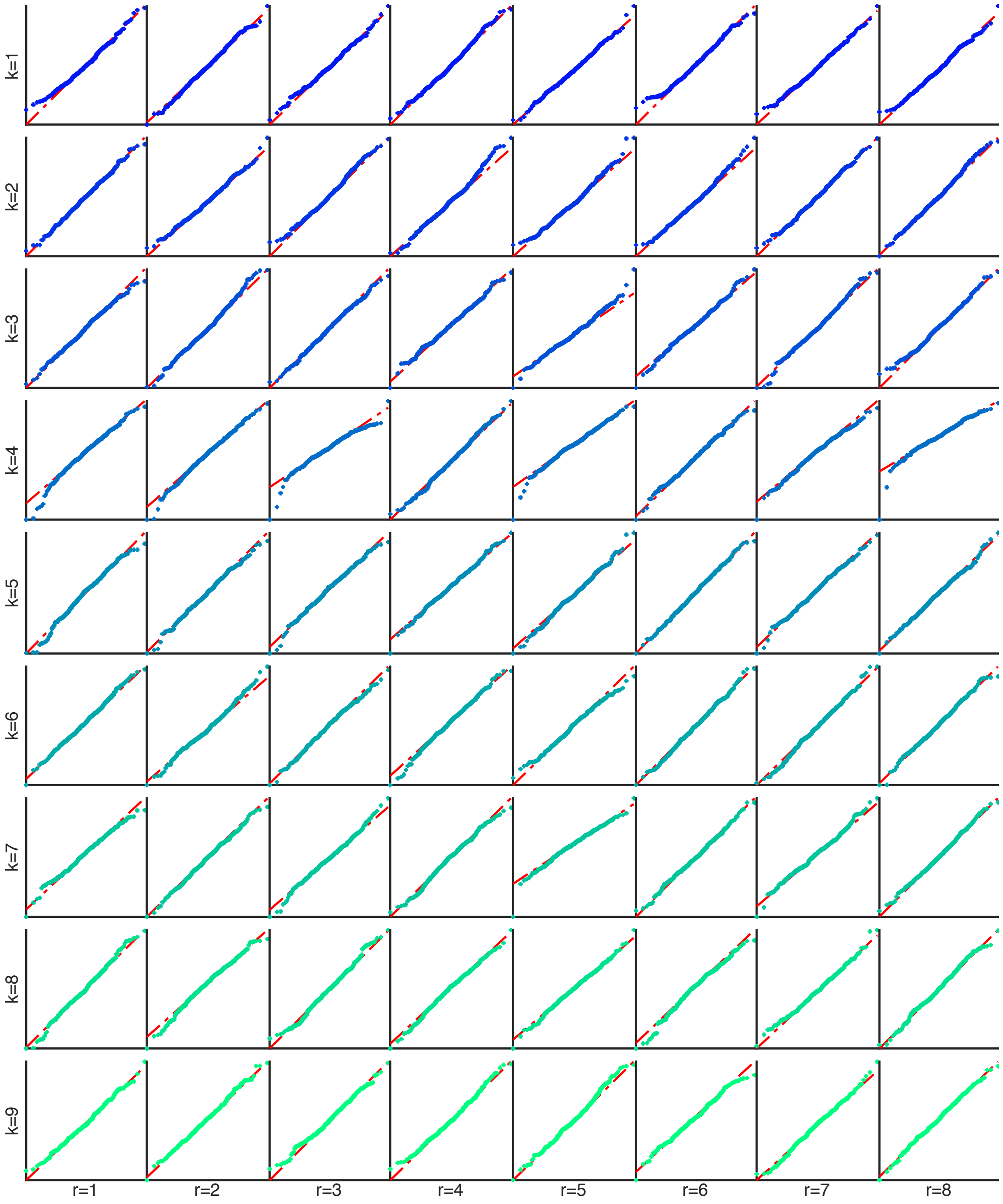

Figure C: Quantile-Quantile (Q-Q) plots of the distribution of the first intersection in the r-th pass, $Q_k^{(r)}$, in transversal coordinates $k = 2, 3, \ldots, 10$ for $r = 1, 2, \ldots, 8$.

4 pcLNA and exact transversal distributions

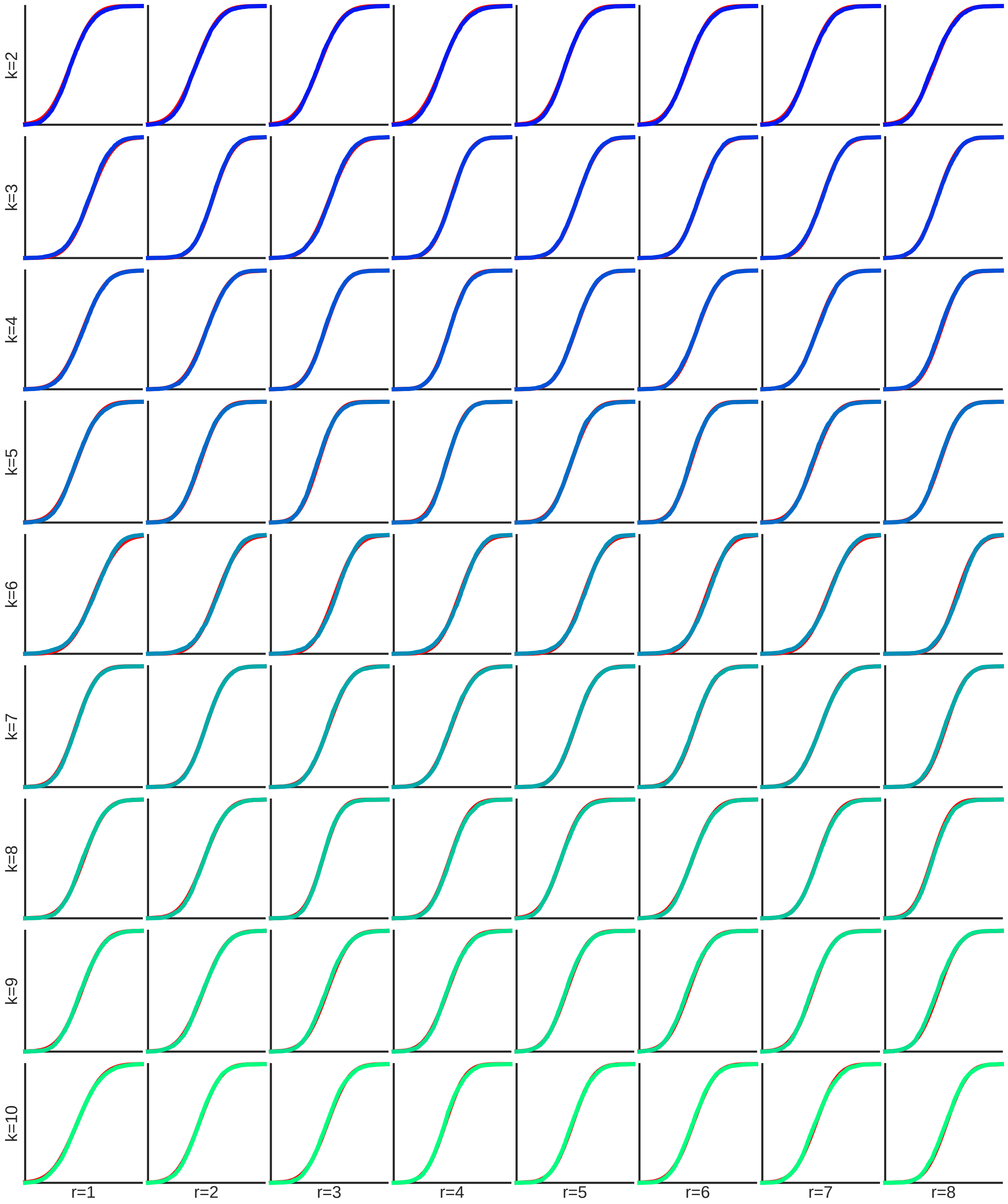

Figure D: CDF plots of the transversal distributions $Q_k^{(r)}$ under the pcLNA (red line) and the SSA (empirical CDF, crosses) for transversal coordinates $k = 2, 3, \ldots, 10$ and round $r = 1, 2, \ldots, 8$.

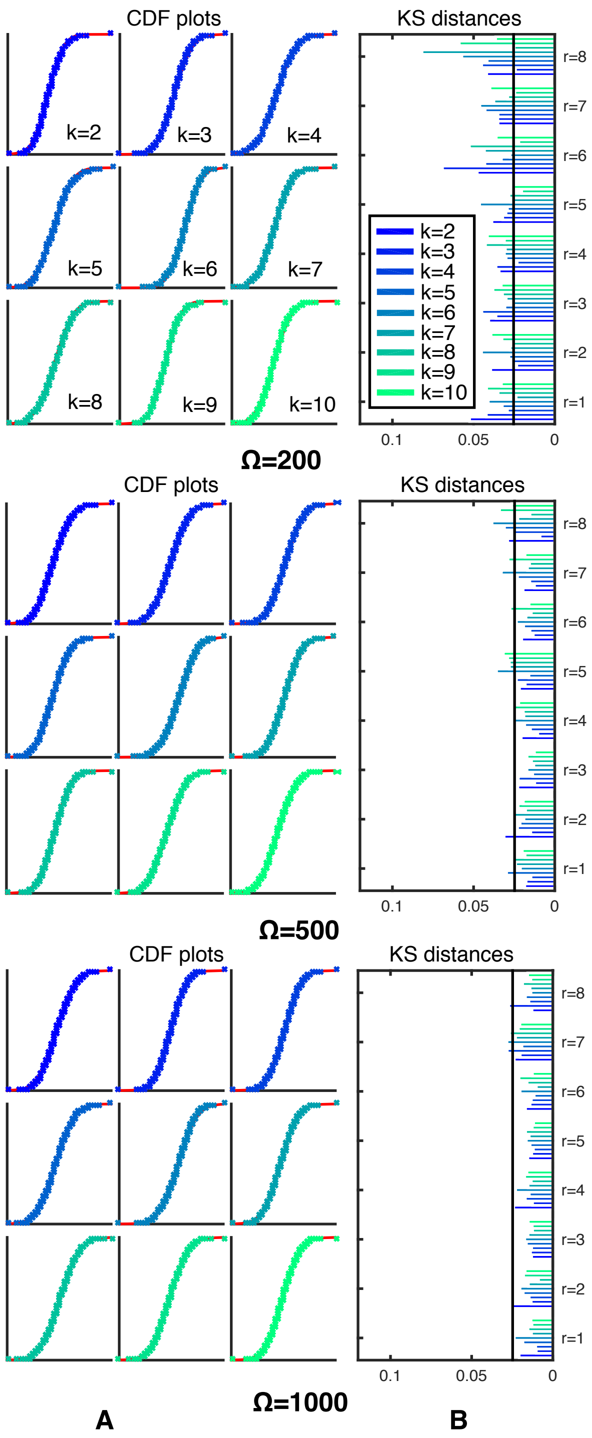

Figure E: Comparison of pcLNA and exact transversal distributions in systems sizes $\Omega = 200$ (top panel), $\Omega = 500$ (middle panel), $\Omega = 1000$ (bottom panel).(A) CDF plots under the pcLNA (red line) and the SSA (empirical CDF, crosses) of the first intersection in the r-th pass, $Q_k^{(r)}$, in transversal coordinates $k = 2, 3, \ldots, 10$ for $r = 4$. (B) KS distances between the $Q_k^{(r)}$ distributions under pcLNA and SSA, $r = 1, 2, \ldots, 8$, $k = 2, 3, \ldots, 10$.

5 Less frequent correction

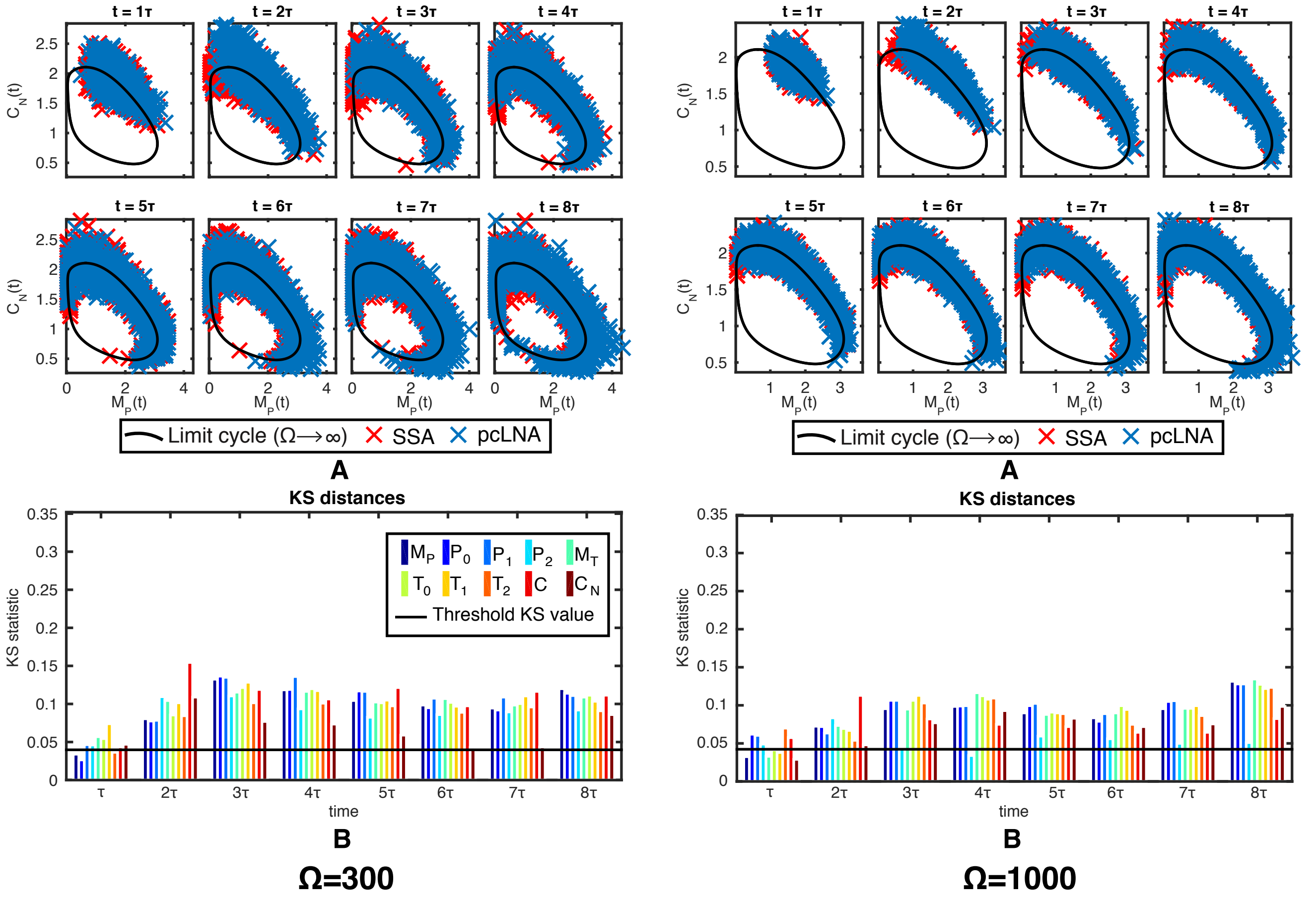

Figure F: Comparison between pcLNA and SSA simulations for correction frequency $24h$. This is 3 times less frequent than the correction used in the simulations presented in Fig. 9 of **I** and the figures in the next section. Panels (A) contain the samples produced by the pcLNA and the exact simulation (SSA) at time-points, $t = 1\tau, 2\tau, \ldots, 8\tau$, for $\Omega = 300$ (left panel) and $\Omega = 1000$ (right panel), and panels (B) the KS distances between the empirical distributions of pcLNA and the SSA for each system variable (colored bars) for two system sizes $\Omega = 300$ (left panel) and $\Omega = 1000$ (right panel).

Less frequent phase correction in pcLNA algorithm has substantial impact on pcLNA simulations. However, the precision of the approximation seems to stabilise after the third round with KS distances much smaller compared to standard LNA. The median CPU times under this correction frequency are $0.45 secs$ for $\Omega = 300$ and $0.22 secs$ for $\Omega = 1000$, compared to $0.45 secs$ and $0.42 secs$, respectively, for correction frequency $6h$. The reason for the absence of speed improvement for $\Omega = 300$ is because at this system size and with less corrections negative populations appear more frequently and therefore SSA simulation are used more frequently, as explained in Sec. 13 in **S**1, and therefore any improvements due to applying correction less frequently are counterbalanced. For $\Omega = 1000$, where negative populations are much less likely the speed improvement, is almost twofold.

6 Comparison between different simulation algorithms

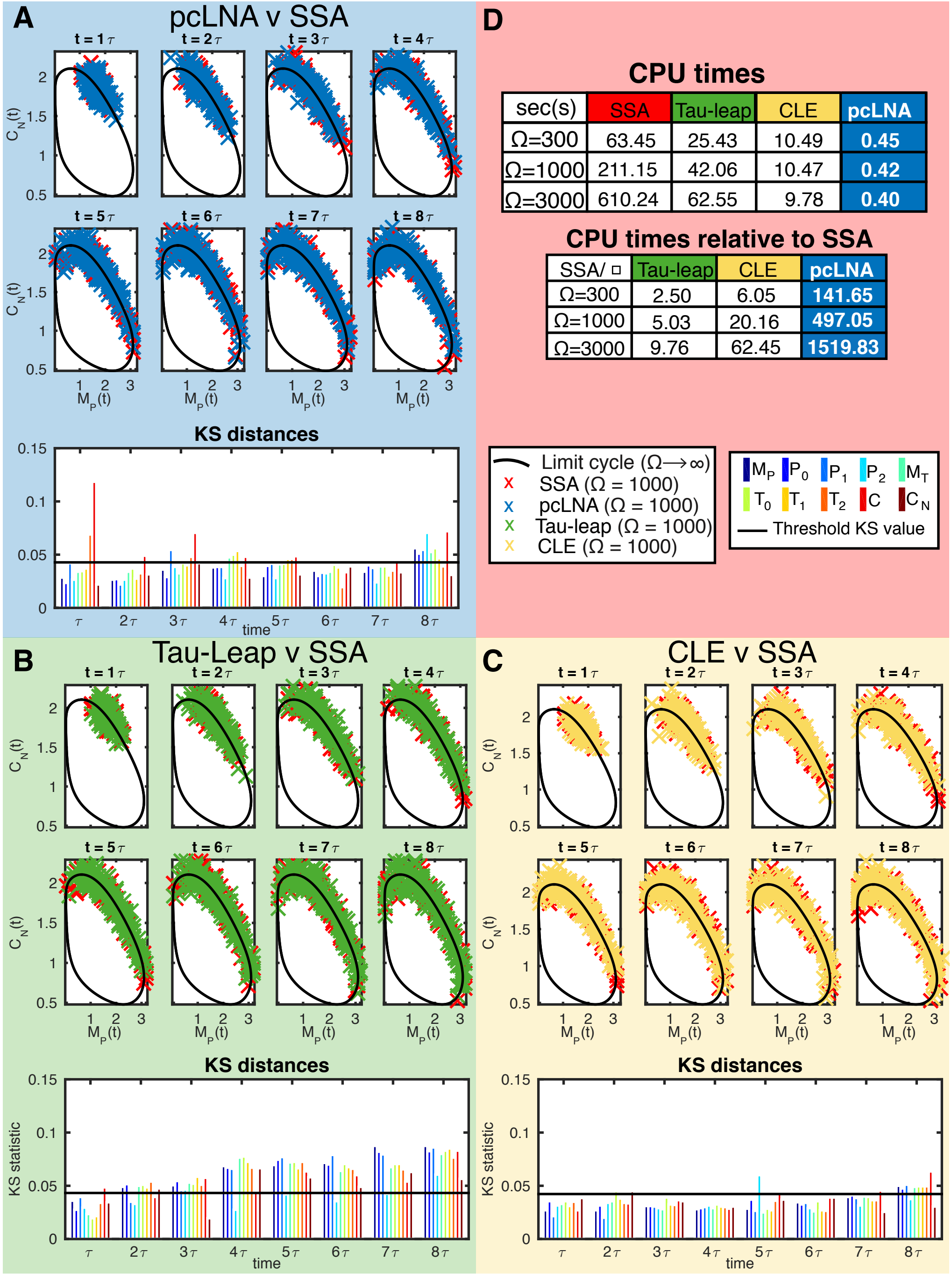

Figure G: Comparison between pcLNA, tau-leap and CLE simulation algorithms for the *Drosophila* circadian clock. This figure has the same form as Fig. 9 of **I**, but (A), (B) and (C) panels contain results for $\Omega = 1000$. As in Fig. 9 of **I**, the parameter values $\epsilon = 0.002$ and $\Delta t = 0.002$ for the tau-leap and the CLE approximation are the largest values to achieve precision similar to the pcLNA simulation, and the results displayed here are simulated using these values.

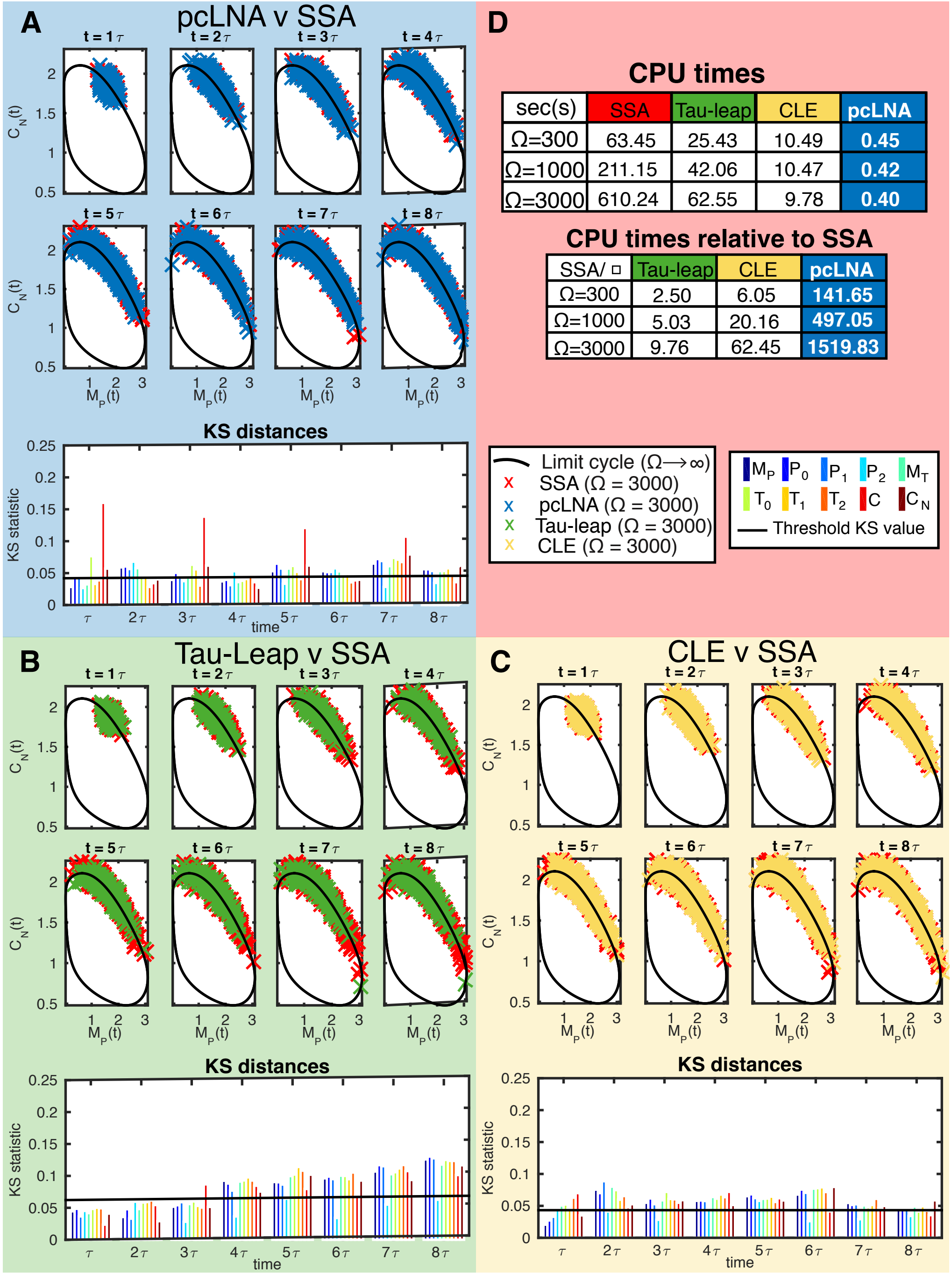

Figure H: Comparison between pcLNA, tau-leap and CLE simulation algorithms for the *Drosophila* circadian clock. This figure has the same form as Fig. 9 of **I**, but (A), (B) and (C) panels contain results for $\Omega = 3000$. As in Fig. 9 of **I**, the parameter values $\epsilon = 0.002$ and $\Delta t = 0.002$ for the tau-leap and the CLE approximation are the largest values to achieve precision similar to the pcLNA simulation, and the results displayed here are simulated using these values.

7 Light entrained *D*rosophila Circadian Clock

The Light entrained *Drosophila* circadian clock system first proposed in [2] is exactly the same with the *Drosophila* circadian clock without entrainment, apart from the degradation rate, v_{dT}, of the phosphorylated TIM protein, T_2, which instead of being equal to 3.4, it is equal to 4 during the day-time (6-18h) and equal to 2 during night-time (18h-6h).

We first compare the LNA distributions at fixed times with the empirical distributions derived using SSA simulations. As we can see in Fig. I, despite that the empirical distributions of the exact stochastic model do not spread along the curved limit cycle as much as the corresponding distributions for the system without entrainment (see Fig. 3 of **I**) and that the spread of the distributions in the light entrained system appears to stabilise even after the second cycle, the standard LNA distributions still do not approximate well these distributions.

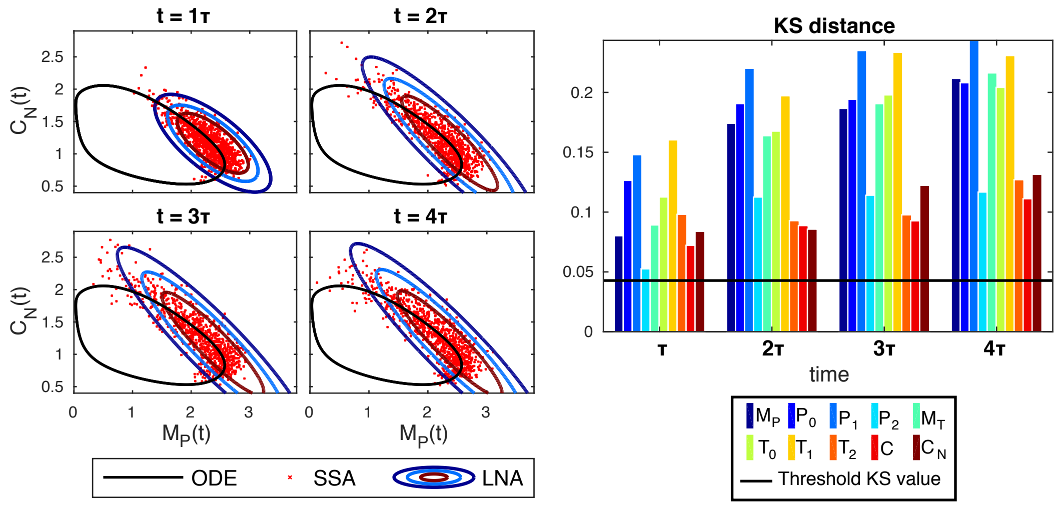

Figure I: Comparison between LNA and exact simulations in the light entrained *Drosophila* circadian clock. (a) Samples (in nanomolar concentrations) obtained from the SSA simulation algorithm (red crosses) and $0.01, 0.05, 0.40$ contours of the LNA probability density (black ellipsoids) at fixed times, $t = \tau, 2\tau, 3\tau, 4\tau$ (τ: minimal period). The limit cycle ODE solution is also displayed (black solid line). (b) KS distance between the empirical distribution of SSA samples and the LNA distribution of each species (different colors, see legend) at the fixed times. The threshold level is also displayed (black solid line). The system size is $\Omega = 300$.

On contrary, as we can see in Fig. J, the pcLNA distributions appear to approximate well the empirical transversal distributions derived under the SSA.

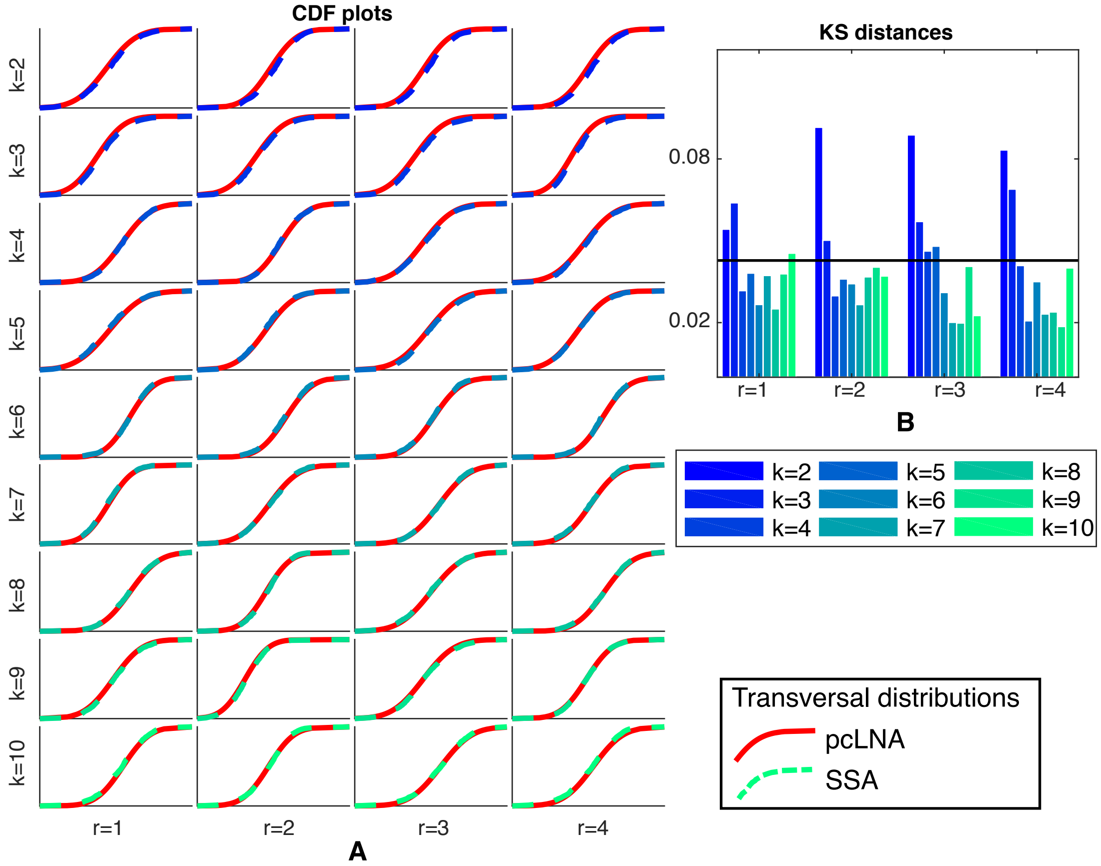

Figure J: Comparison of pcLNA and exact empirical transversal distributions of the light entrained *Drosophila* circadian clock. (A) CDF plots of the transversal distributions $Q_k^{(r)}$ under the pcLNA (red line) and the SSA (empirical CDF, colored dashed line, see legend) in transversal coordinates $k = 2, 3, \ldots, 10$ and round $r = 1, 2, 3, 4$. (B) KS distances between the $Q_k^{(r)}$ distributions under the pcLNA and the SSA, $k = 2, 3, \ldots, 10$, $r = 1, 2, 3, 4$. The system size is $\Omega = 300$.

References

[1] Gonze D, Halloy J, Leloup JC, Goldbeter A. Stochastic model for circadian rhythms: effect of molecular noise on periodic and chaotic behaviour. Comptes Rendus Biologies 2003 Apr;326(2):189–203.

[2] Leloup JC & Goldbeter A. A Model for Circadian Rhythms in Drosophila Incorporating the Formation of a Complex between the PER and TIM Proteins. Journal of Biological Rhythms 1998 Feb; 13(1): 70-87.
