# Supplementary material for: Long-time analytic approximation of large stochastic oscillators: Simulation, analysis and inference
Source: PLoS Comput Biol. 2017 Jul 24;13(7):e1005676. doi: 10.1371/journal.pcbi.1005676 (PMC5555717; doi:10.1371/journal.pcbi.1005676)
Supplement: S3 Appendix — In this note we give details about the deterministic and stochastic models of the Brusselator and NF-κB systems and use them to illustrate further the results described in the main paper. (PDF) [file pcbi.1005676.s003.pdf]

Supplementary information S3 for the paper Long-time analytic approximation of large stochastic oscillators: simulation, analysis and inference

Giorgos Minas[1,2] & David A Rand[1,2]

[1] Zeeman Institute for Systems Biology & Infectious Disease Epidemiology Research & [2]Mathematics Institute,

University of Warwick, Coventry CV4 7AL, UK

July 14, 2017

Contents

1 Brusselator system **1**

1.1 Fixed point system 2

2 NF-κB system **3**

2.1 The NF-κB response to TNFα activation 9

Abstract

We refer to the paper "Long-time analytic approximation of large stochastic oscillators: simulation, analysis and inference" by **I**. In this note we give further illustrations for the Brusselator and the NF-κB system.

1 Brusselator system

The ODE system of the Brusselator is

$$\begin{aligned}\dot{A} &= 1 - A(1 + b - cAB),\\ \dot{B} &= A(b - cAB).\end{aligned}$$

For the limit cycle ODE solution, we use the initial conditions $A(t_0) = 0.75$, $B(t_0) = 2.00$, and the parameter values $b = 2.20$, $c = 1.00$. We use the SSA to exactly simulate the system

and produce $R = 3000$ samples of stochastic trajectories (see Figure A) for a time-length of $8.5 \times \tau$, where $\tau \approx 6.37$ is the period of the periodic solution of the ODE. The rates of the reactions used for the SSA of the Brusselator are provided in Table A.

reaction	rate
$\emptyset \xrightarrow{1} A$	Ω
$A \xrightarrow{1} \emptyset$	A
$A \xrightarrow{b} B$	bA
$B \xrightarrow{c} A$	cA^2B/Ω^2

Table A: Reactions of the Brusselator system and their rates

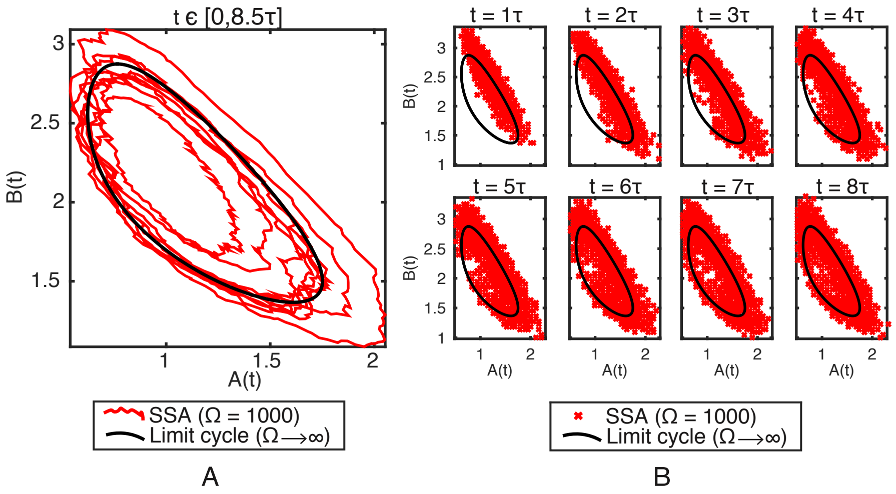

Figure A: Exact stochastic simulation of the Brusselator system. (A) A stochastic trajectory of $X(t) = Y(t)/\Omega$ obtained by running the SSA over the time-interval $t \in [0, 8.5\tau]$ and (B) SSA samples ($R = 3000$) at times $t = \tau, 2\tau, \ldots, 8\tau$. The volume size is $\Omega = 1000$. The black solid curve is the large volume, $\Omega \to \infty$, limit cycle solution.

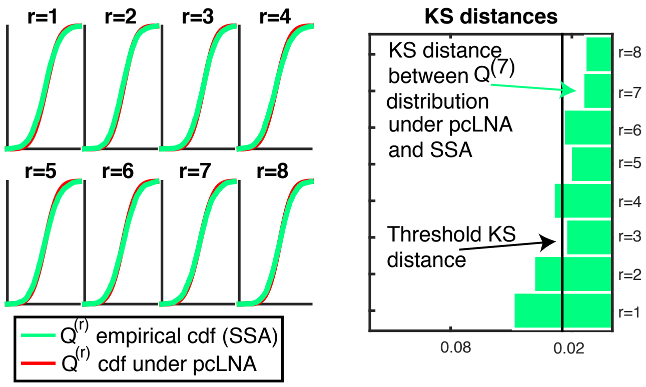

Figure B: Comparison of pcLNA and exact empirical transversal distributions. (A) CDF plots of the (one-dim) transversal distributions $Q^{(r)}$ under the pcLNA (red line) and the SSA (empirical CDF, crosses) in round $r = 1, 2, \ldots, 8$. (B) KS distances between the $Q_k^{(r)}$ distributions under pcLNA and SSA, $r = 1, 2, \ldots, 8$.

1.1 Fixed point system

For comparisons, we also consider a set of parameter values that give a limiting system ($\Omega \to \infty$) with an equilibrium fixed point instead of a limit cycle. Here, the initial conditions are $A(t_0) = 1.156$, $B(t_0) = 1.461$, and the parameter values $b = 0.25$, $c = 0.25$, which give an equilibrium point $A_{eq} = B_{eq} = 1$ with Jacobian matrix of the system (referred as linearisation matrix in **I**) with both eigenvalues equal to -0.5.

We use the SSA to exactly simulate this system and produce $R = 2000$ samples of stochastic trajectories (see Fig. CA) for a time-length of 700, which is somewhat larger than 100τ, where τ the period of the limit cycle ODE solution considered above. In Fig. CB we provide the empirical CDF plots of the SSA at time $637 \approx 100\tau$ and compare them with the LNA distributions at the same time.

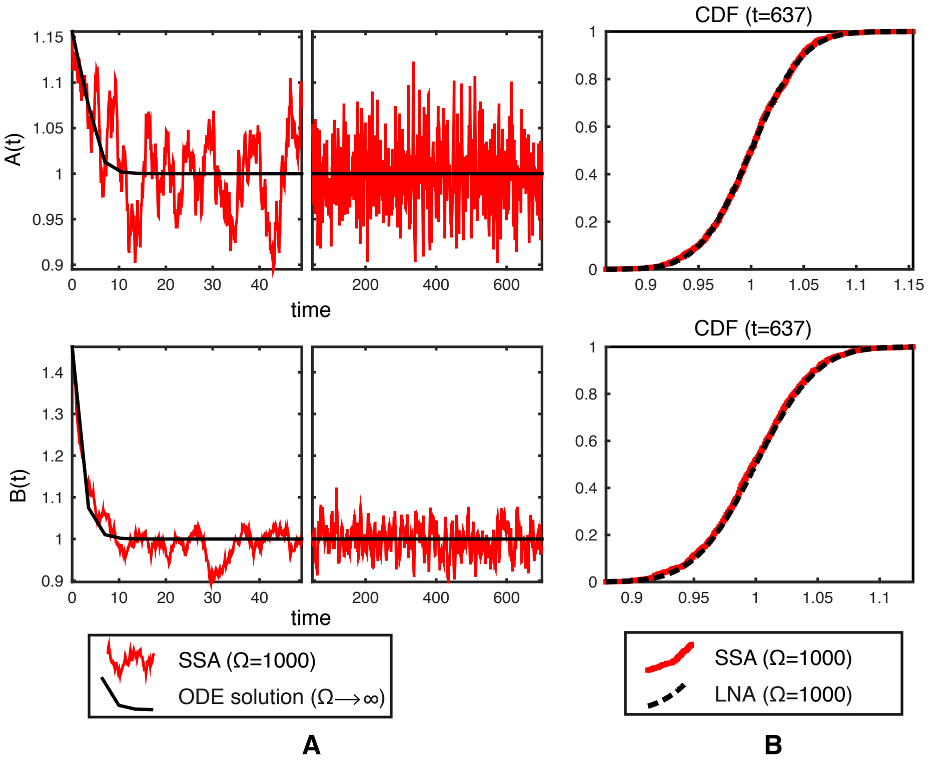

Figure C: Exact stochastic simulation and comparison to the LNA for the fixed point Brusselator system. The parameter values are $b = c = 0.25$ and the system size $\Omega = 1000$. (A) A stochastic trajectory of $X(t) = Y(t)/\Omega$ for the two variables A (top panel) and B (bottom panel) of the Brusselator system obtained by running the SSA over the time-interval $t \in [0, 700]$ and its large volume, $\Omega \to \infty$, solution. (B) Empirical CDF plots of the SSA samples ($R = 2000$) and CDF plots of the LNA at time $t = 637$ for variables A (top panel) and B (bottom panel).

2 NF-κB system

The NF-κB system model used in this SI describes the oscillatory response of the system following stimulation by tumor necrosis factor alpha (TNFα). With no stimulation the system

remains in a stable equilbrium state. Following TNFα stimulation that is constant in time the system responds by a transient pulse followed by relaxation to a stable limit cycle.

We first consider the case where the system starts in the stable limit cycle solution, and in the next section consider the biologically interesting situation where the signal is received while the system is inactive and the initial oscillation is transitory. The species of the system and the initial conditions (in micromolar concentrations) used to derive the limit cycle deterministic solution are provided in Table B.

	name	description	initial value
1	N_c	Free Cytoplasmic NFkB	0.0056
2	I_c	Free Cytoplasmic IkBa	0.0298
3	NI_c	Cytoplasmic NFkB-IkBa	0.0723
4	N_n	Free nuclear NFkB	0.0011
5	I_n	Free nuclear IkBa	0.0075
6	NI_n	Nuclear NFkB-IkBa	0.0013
7	I_m	IkBa transcription	0.0004
8	K_n	Kinase IKKn	0.0020
9	K_a	Kinase IKKa	0.0024
10	A_m	A20 transcription	0.0001
11	A	A20	0.0076

Table B: The variables of NF-κB system and the initial conditions (in concentrations) used to derive the limit cycle ODE solution.

The system model considered here is a slight modification of the system model in [1]. In particular, some variables are omitted because the sum of the concentrations of all forms of NF-κB and IKK are both fixed and the level of phosphorylated IkBa is an end state that plays no further role in the model. Therefore, the redundant species IKKi, phosphorylated IkBα and phosphorylated NFκB-IκBα are removed to avoid rank-deficiencies in the covariance matrices of the LNA.

We also write the system in a form where concentrations are all written in terms of the same volume i.e. the total cell volume. The original system in [1] is written in cytoplasmic concentrations for all species except, N_n, I_n and NI_n that are written in nuclear concentrations and are set to be 3.3 larger than cytoplasmic concentrations. The nuclear and cyctoplasmic concentrations can easily be recovered from our concentration if one knows the ratio of nuclear to cytoplasmic volume.

We used the same parameter values as in [1] to derive the ODE solution. These are provided in Table C.

parameter	description	value	measurement unit
k_v	C:N ratio	3.300000	–
k_p	IKKn production	1.3030e-04	s^{-1}
k_a	Activation caused by TNFa	0.003	s^{-1}
k_i	Spontaneous IKK activation	0.002	s^{-1}
k_{a1a}	NFkB-IkBa association	0.5	$\mu M^{-1}s^{-1}$
k_{d1a}	NFkB-IkBa dissociation	0.0005	s^{-1}
k_{c1a}	Catalysis of IKK-IkBa dimer	0.037	s^{-1}
k_{c2a}	Catalysis of IKK-IkBa-NFkB trimer	0.185	s^{-1}
k_{t1a}	degradation of IkBa (IKK dependent from dimer)	0.025	s^{-1}
k_{t2a}	degradation of IkBa (IKK dependent from trimer)	0.025	s^{-1}
c_{4a}	Free IkBa degradation	0.00019	s^{-1}
c_{5a}	NFkB complexed IkBa degradation	0.000022	s^{-1}
k_{i1}	NFkB nuclear import	0.003	s^{-1}
k_{e1}	NFkB nuclear export	6.0000e-05	s^{-1}
k_{e2a}	NFkB-IkBa nuclear export	0.012	s^{-1}
k_{i3a}	IkBa nuclear import	0.001	s^{-1}
k_{e3a}	IkBa nuclear export	0.0005	s^{-1}
h	Order of hill function	2	–
k	Hill constant	0.0430	–
c_{1a}	IkBa mRNA synthesis	1.4200e-07	$\mu M^{-1}s^{-1}$
c_{2a}	IkBa translation rate	0.5	s^{-1}
c_{3a}	IkBa mRNA degradation	0.00048	s^{-1}
c_1	IkBa mRNA synthesis	1.4200e-07	$\mu M^{-1}s^{-1}$
c_2	A20 mRNA translation	0.5	s^{-1}
c_3	A20 mRNA degradation	0.00048	s^{-1}
c_4	A20 degradation	0.0045	s^{-1}
k_{bA20}	Half-max A20 inhibition concentration	0.0018	–
TNFα	Tumor necrosis factor alpha level	100	ng/ml
TNF-κB	total NF-κB concentration	0.08	μM
$TIKK$	total IKK concentration	0.08	μM

Table C: The parameters of NF-κB system and the values used to derive their ODE solution.

The ODE system for the NF-κB system considered in this SI is:

$$\dot{N}_c = k_{d1a} NI_c - k_{a1a} N_c I_c - k_{i1} N_c + c_{5a} NI_c + k_v k_{e1} N_n + k_{t2a} \times (TNFKB - N_c - NI_c - N_n + NI_n)$$

$$\dot{I}_c = k_{d1a} NI_c - k_{a1a} N_c I_c - k_{i3a} I_c + k_v k_{e3a} I_n - c_{4a} I_c + c_{2a} I_m - k_{c1a} K_a I_c$$

$$\dot{NI}_c = k_{a1a} N_c I_c - k_{d1a} NI_c + k_v k_{e2a} NI_n - c_{5a} NI_c - k_{c2a} K_a NI_c$$

$$\dot{N}_n = k_{d1a} NI_n - k_v k_{a1a} N_n I_n + k_{i1} N_c - k_v k_{e1} N_n$$

$$\dot{I}_n = k_{d1a} NI_n - k_v k_{a1a} N_n I_n + k_{i3a} I_c - k_v k_{e3a} I_n - c_{4a} I_n$$

$$\dot{NI}_n = k_v k_{a1a} NI_n - k_{d1a} NI_n - k_v k_{e2a} NI_n$$

$$\dot{I}_m = c_{1a}(N_n^h / (N_n^h + (k/k_v)^h)) - c_{3a} I_m$$

$$\dot{K}_n = k_p (TIKK - K_n - K_a)(k_{bA20} / (k_{bA20} + A \times TNF\alpha)) - k_a TNF\alpha K_n$$

$$\dot{K}_a = k_a TNF\alpha K_n - k_i K_a$$

$$\dot{A}_m = c_1 (N_n^h / (N_n^h + (k/k_v)^h)) - c_3 A_m$$

$$\dot{A} = c_2 A_m - c_4 A$$

The reaction rates used for the SSA are provided in Table D. The values of the parameters are the same as in Table C.

reaction	rate
$N_c + I_c \xrightarrow{k_{a1a}} NI_c$	$k_{a1a} \times I_c \times N_c/\Omega$
$NI_c \xrightarrow{k_{d1a}} N_c + I_c$	$k_{d1a} \times NI_c$
$N_n + I_n \xrightarrow{k_{a1a}} NI_n$	$k_v k_{a1a} \times I_n \times N_n/\Omega$
$NI_n \xrightarrow{k_{d1a}} N_n + I_n$	$k_{d1an} \times NI_n$
$K_a + I_c \xrightarrow{k_{c1a}} K_a + I_p$	$k_{c1a} \times K_a \times I_c/\Omega$
$K_a + NI_c \xrightarrow{k_{c2a}} K_a + NI_p$	$k_{c2a} \times K_a \times NI_c/\Omega$
$NI_p \xrightarrow{k_{t2a}} N_c$	$k_{t2a} \times NI_p$
$N_c \xrightarrow{k_{i1}} N_n$	$k_{i1} \times N_c$
$N_n \xrightarrow{k_{e1}} N_c$	$k_{e1} \times k_v \times N_n$
$NI_n \xrightarrow{k_{e2a}} NI_c$	$k_{e2a} \times k_v \times NI_n$
$I_c \xrightarrow{k_{i3a}} I_n$	$k_{i3a} \times I_c$
$I_n \xrightarrow{k_{e3a}} I_c$	$k_{e3a} \times k_v \times I_c$
$\varnothing \xrightarrow{H_I} I_m$	$(c_{1a}\Omega)\frac{N_n^h}{N_n^h+(k\Omega/k_v)^h}$
$I_m \xrightarrow{c_{2a}} I_m + I_c$	$c_{2a} \times I_m$
$I_m \xrightarrow{c_{3a}} \varnothing$	$c_{3a} \times I_m$
$I_c \xrightarrow{c_{4a}} \varnothing$	$c_{4a} \times I_c$
$I_n \xrightarrow{c_{4a}} \varnothing$	$c_{4a} \times I_n$
$NI_c \xrightarrow{c_{5a}} N_c$	$c_{5a} \times NI_c$
$\varnothing \xrightarrow{H_A} A_m$	$(c_1\Omega)\frac{N_n^h}{N_n^h+(k\Omega/k_v)^h}$
$A_m \xrightarrow{c_2} A_m + A$	$c_2 \times A_m$
v $A_m \xrightarrow{c_3} \varnothing$	$c_3 \times A_m$
$A \xrightarrow{c_4} \varnothing$	$c_4 \times A$
$K_i \xrightarrow{M_A} K_n$	$k_p(TIKK - K_n - K_a)\frac{k_{bA20}\times\Omega}{(k_{bA20}\Omega)+A\times TNF\alpha}$
$K_n \xrightarrow{TNF\alpha\times k_a} K_a$	$TNF\alpha \times k_a \times K_n$
$K_a \xrightarrow{k_i} K_i$	$k_i \times K_a$

Table D: Reactions of the NF-κB system and their rates

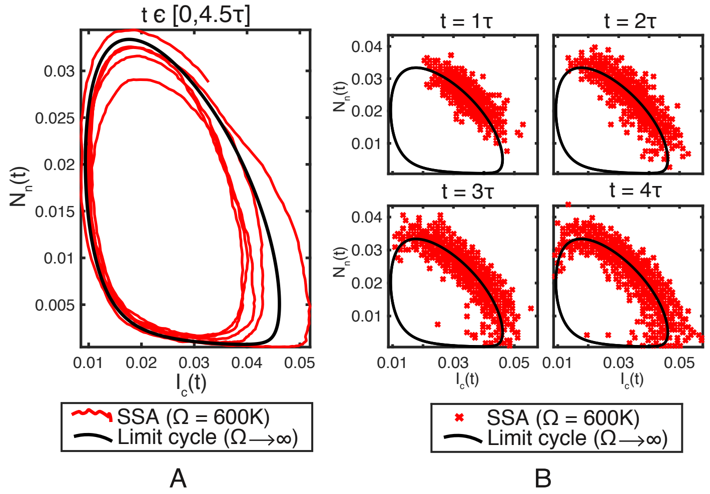

Figure D: Exact stochastic simulation of the NF-κB system. (A) A stochastic trajectory (in concentrations) obtained by running the SSA over the time-interval $t \in [0, 4.5\tau]$ and (B) SSA samples ($R = 3000$) at times $t = \tau, 2\tau, 3\tau, 4\tau$. Two (out of 11) of the species are displayed (IκBα I_C (x-axis) and nuclear NF-κB N_n (y-axis)). The volume size is $\Omega = 0.6M$. This is substantially smaller than the system size used in [1] ($\Omega = 1.25M$) to provide higher noise levels. The black solid curve is the large volume, $\Omega \to \infty$, limit cycle solution.

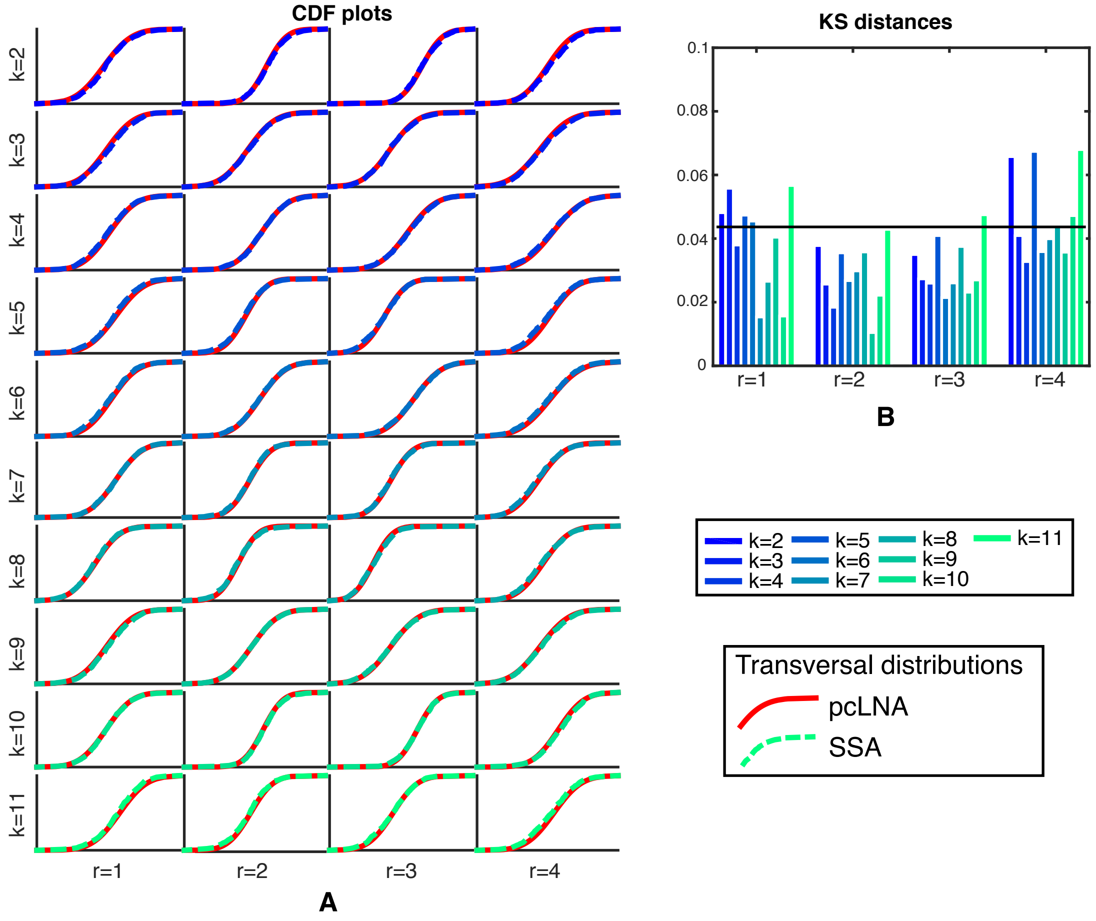

Figure E: Comparison of pcLNA and exact transversal distributions. (A) CDF plots of the transversal distributions $Q_k^{(r)}$ under the pcLNA (red line) and the SSA (empirical CDF, colored dashed line, see legend) in transversal coordinates $k = 2, 3, \ldots, 11$ and round $r = 1, 2, 3, 4$. (B) KS distances between the $Q_k^{(r)}$ distributions under pcLNA and SSA, $k = 2, 3, \ldots, 11$, $r = 1, 2, 3, 4$. The system size is $\Omega = 0.6M$.

2.1 The NF-κB response to TNFα activation

Here we study the response of the *NF-κB* system to a continuous TNFα signal received while the system being in the equilibrium fixed point state. This equilibrium state which is given in Table E is therefore the initial conditions used to derive the deterministic solution provided in Fig. F.

	name	initial value
1	N_c	0.0036
2	I_c	0.0160
3	NI_c	0.0719
4	N_n	0.0042
5	I_n	0.0013
6	NI_n	0.0003
7	I_m	0.0000
8	K_n	0.0800
9	K_a	0
10	A_m	0.0000
11	A	0.0014

Table E: The initial conditions (in concentrations) used to derive the ODE solution in Fig 6.

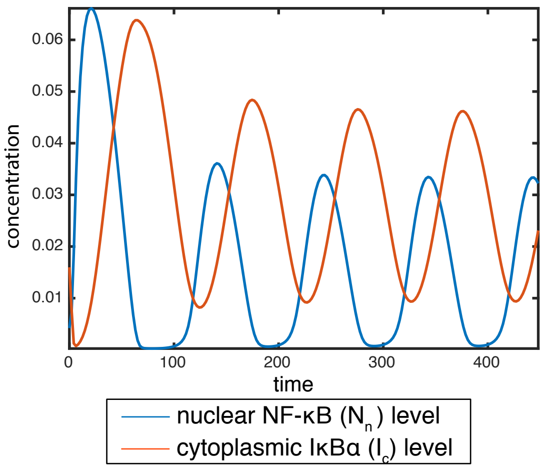

Figure F: The limiting ($\Omega \to \infty$) deterministic solution of the NF-κB system. The initial conditions are provided in Table E. Two species of the system are displayed.

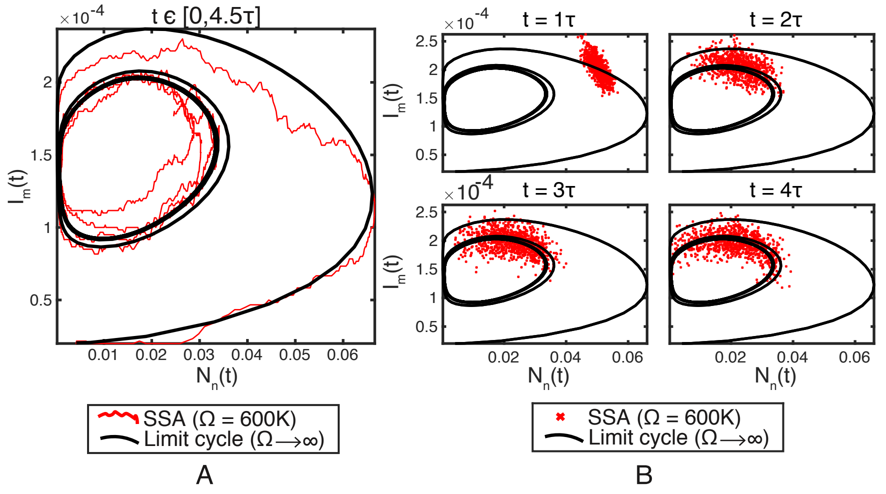

Figure G: Exact stochastic simulation of the NF-κB system. (A) A stochastic trajectory (in concentrations) obtained by running the SSA over the time-interval $t \in [0, 4.5\tau]$ and (B) SSA samples ($R = 3000$) at times $t = \tau, 2\tau, 3\tau, 4\tau$. Two (out of 11) of the species are displayed (nuclear NF-κB (x-axis) and IκBα mRNA (y-axis)). The volume size is $\Omega = 0.6M$. The black solid curve is the large volume, $\Omega \to \infty$, limit cycle solution.

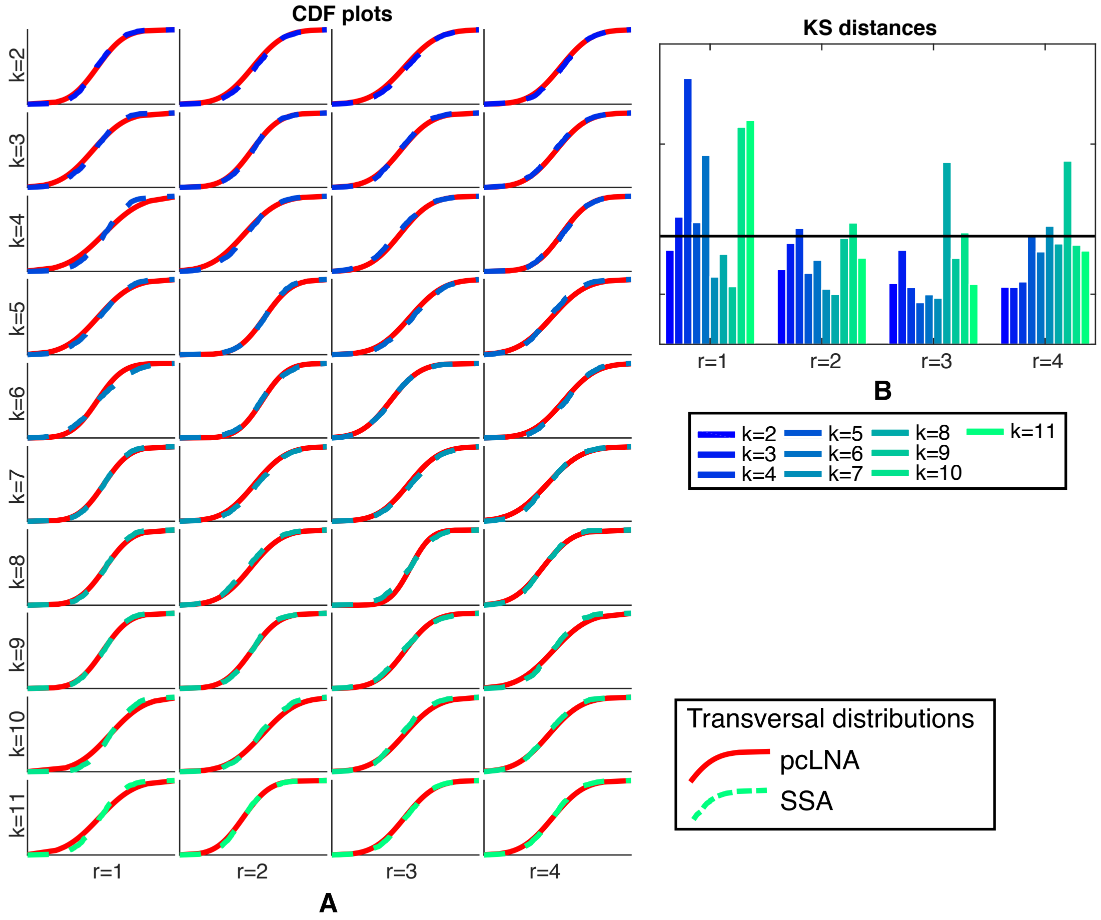

Figure H: Comparison of pcLNA and exact transversal distributions. (A) CDF plots of the transversal distributions $Q_k^{(r)}$ under the pcLNA (red line) and the SSA (empirical CDF, colored dashed line, see legend) in transversal coordinates $k = 2, 3, \ldots, 11$ and round $r = 1, 2, 3, 4$. (B) KS distances between the $Q_k^{(r)}$ distributions under pcLNA and SSA, $k = 2, 3, \ldots, 11$, $r = 1, 2, 3, 4$. The system size is $\Omega = 0.6M$.

References

[1] Ashall L, Horton CA, Nelson DE, Paszek P, Harper CV, Sillitoe K, Ryan S, Spiller DG, Unitt JF, Broomhead DS, Kell DB, Rand DA, Sée V, White MRH. Pulsatile stimulation determines timing and specificity of NF-kappa B-dependent transcription. Science. 2009 Apr;324(5924);242–6.
